# SLAM‐Drop‐seq reveals mRNA kinetic rates throughout the cell cycle

**DOI:** 10.15252/msb.202211427

**Published:** 2023-08-28

**Authors:** Haiyue Liu, Roberto Arsiè, Daniel Schwabe, Marcel Schilling, Igor Minia, Jonathan Alles, Anastasiya Boltengagen, Christine Kocks, Martin Falcke, Nir Friedman, Markus Landthaler, Nikolaus Rajewsky

**Affiliations:** ^1^ Berlin Institute for Medical Systems Biology Max Delbrück Center for Molecular Medicine in the Helmholtz Association Berlin Germany; ^2^ Mathematical Cell Physiology Max Delbrück Center for Molecular Medicine in the Helmholtz Association Berlin Germany; ^3^ Lübeck Interdisciplinary Platform for Genome Analytics (LIGA) University of Lübeck Lübeck Germany; ^4^ Department of Physics Humboldt University Berlin Berlin Germany; ^5^ The Rachel and Selim Benin School of Computer Science and Engineering The Hebrew University of Jerusalem Jerusalem Israel; ^6^ The Lautenberg Center for Immunology and Cancer Research, Institute of Medical Research Israel‐Canada (IMRIC), Faculty of Medicine The Hebrew University of Jerusalem Jerusalem Israel; ^7^ The Center for Computational Medicine, Faculty of Medicine The Hebrew University of Jerusalem Jerusalem Israel; ^8^ Institut für Biologie Humboldt Universität zu Berlin Berlin Germany; ^9^ Charité ‐ Universitätsmedizin Berlin Corporate Member of Freie Universität Berlin and Humboldt‐Universität zu Berlin Berlin Germany; ^10^ German Center for Cardiovascular Research (DZHK) Berlin Germany; ^11^ NeuroCure Cluster of Excellence Berlin Germany; ^12^ German Cancer Consortium (DKTK) Berlin Germany; ^13^ National Center for Tumor Diseases (NCT) Berlin Germany

**Keywords:** cell cycle, mRNA kinetics, single cells, temporal regulation, transcription and degradation, Methods & Resources, RNA Biology

## Abstract

RNA abundance is tightly regulated in eukaryotic cells by modulating the kinetic rates of RNA production, processing, and degradation. To date, little is known about time‐dependent kinetic rates during dynamic processes. Here, we present SLAM‐Drop‐seq, a method that combines RNA metabolic labeling and alkylation of modified nucleotides in methanol‐fixed cells with droplet‐based sequencing to detect newly synthesized and preexisting mRNAs in single cells. As a first application, we sequenced 7280 HEK293 cells and calculated gene‐specific kinetic rates during the cell cycle using the novel package Eskrate. Of the 377 robust‐cycling genes that we identified, only a minor fraction is regulated solely by either dynamic transcription or degradation (6 and 4%, respectively). By contrast, the vast majority (89%) exhibit dynamically regulated transcription and degradation rates during the cell cycle. Our study thus shows that temporally regulated mRNA degradation is fundamental for the correct expression of a majority of cycling genes. SLAM‐Drop‐seq, combined with Eskrate, is a powerful approach to understanding the underlying mRNA kinetics of single‐cell gene expression dynamics in continuous biological processes.

## Graphical abstract

A novel approach (SLAM‐Drop‐seq) is coupled to a robust computational analysis method (Eskrate) to discriminate new and preexisting transcripts in single cells and to calculate their time‐dependent kinetic rates of synthesis and decay during the cell cycle.

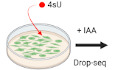

## Introduction

Progression through the cell cycle requires tight control of transcriptional (Dynlacht, 1997) and posttranscriptional (Blackinton & Keene, 2014) regulation to prevent deregulation that trigger cell death or lead to abnormal growth (Schafer, 1998). Many of the genes involved in coordinating this complex process are fine‐tuned at specific stages during the cell cycle, and their dysregulation can lead to various diseases, most notably cancer (Hanahan & Weinberg, 2011; Rubin *et al*, 2020; Matthews *et al*, 2022). While the dynamics of gene expression have been extensively characterized in the cell cycle (Iyer *et al*, 1999; Cho *et al*, 2001; Whitfield *et al*, 2002; Liu *et al*, 2017), little is known how the underlying processes of mRNA transcription, splicing, and degradation influence gene expression and how their associated kinetic rates change depending on the cell cycle time.

Common approaches to studying RNA kinetic rates rely on 4‐thiouridine (4sU) incorporation into nascent transcripts, followed by biochemical enrichment of labeled RNAs (Dölken *et al*, 2008; Miller *et al*, 2011; Rabani *et al*, 2011). To circumvent the main disadvantages associated with these approaches (e.g., high amount of starting material required, unspecific enrichment), SLAM‐seq was developed (Herzog *et al*, 2017): instead of depending on enrichment steps, iodoacetamide (IAA) is used to alkylate the 4sU base in labeled RNAs. This alkylation results in diagnostic thymine‐to‐cytosine (T‐>C) transitions in a reverse‐transcription dependent manner, which are detected and quantified with computational analysis after RNA sequencing. Although instructive, this and similar (Riml *et al*, 2017; Schofield *et al*, 2018) protocols assume RNA kinetic rates to be constant and average gene expressions for millions of cells, flattening the heterogeneity of single cells. In recent years, scRNA‐seq has become the method of choice to analyze the complexity and heterogeneity of cell populations (Hashimshony *et al*, 2012; Macosko *et al*, 2015; Alles *et al*, 2017; Pepe‐Mooney *et al*, 2019). Based on scRNA‐data, gene‐specific RNA synthesis and degradation rates in single cells have been calculated using precursor and mature mRNA counts (Manno *et al*, 2018), but the mRNA decay rate was assumed constant across the cell population. More recently, experimentally more complex single‐cell methods have been established to investigate gene‐specific mRNA kinetic rates based on SLAM‐seq (scSLAM‐seq (Erhard *et al*, 2019), NASC‐seq (Hendriks *et al*, 2019), sci‐fate (Cao *et al*, 2020), scNT‐seq (Qiu *et al*, 2020)). These approaches were described in more detail in a recent review by Erhard *et al* (2022). While these methods all profile whole and newly synthesized transcriptomes, there are important differences: SMART‐seq‐based methods (i.e., scSLAM‐seq, NASC‐seq) normally capture deep coverage of transcripts in each cell, but assay only hundreds of cells due to labor and cost. Sci‐fate relies on single‐cell combinatorial indexing to obtain a high number of single cells. When many cells in heterogeneous populations need to be analyzed, droplet‐based approaches such as scNT‐seq are more advantageous, recovering thousands of cells per experiment with low cost and easy setup. Although all these approaches have been applied to investigate gene‐specific mRNA kinetic rates in different systems, they only address discrete states of cell activation, differentiation, or infection. For biological processes that are continuous and dynamic, it is necessary to investigate the kinetic rates in a continuous time‐resolved manner. For example, in the case of the cell cycle progression, it would be desirable to measure the RNA kinetic rates throughout the cell cycle as a function of time.

To overcome the limitations of current methods for analyzing time‐dependent RNA kinetic rates, we developed SLAM‐Drop‐seq, an approach that employs 4sU metabolic RNA labeling, chemical conversion of labeled transcripts with IAA *in situ* inside fixed cells, followed by droplet‐based scRNA‐seq. The fixation step permeabilizes cell membranes and therefore allows the IAA‐induced alkylation of 4sU residues to take place within intact cells. Compared to scNT‐seq, which also applies droplet‐based sequencing (Qiu *et al*, 2020), this is a novel step that reduces the hands‐on time and complexity of the experiment. Using SLAM‐Drop‐seq, we quantified newly synthesized and preexisting mRNA transcripts in individual cells. Based on a time‐dependent mRNA kinetic rate model, we developed the R package Eskrate to estimate the time‐resolved mRNA kinetic rates. As a first application, we explored the dynamics of RNA kinetic rates along the cell cycle from a population of unsynchronized HEK293 cells. We assigned each cell to a specific time point of the cell cycle using Revelio (Schwabe *et al*, 2020), a recently developed approach to reconstructing the temporal continuity of the cell cycle from single‐cell transcriptomic data of unperturbed cells. We profiled the time‐dependent gene expression transcriptome‐wide and identified 377 robustly cycling genes. The underlying rates of mRNA synthesis and degradation along the cell cycle were then calculated using Eskrate. We found that temporally regulated RNA degradation is considerably involved in cycling gene expression regulation in the cell cycle, and the gene expression of the majority of the analyzed cycling genes is the result of a close interplay between RNA production and decay.

## Results

### Experimental procedure and data

**Figure 1:**
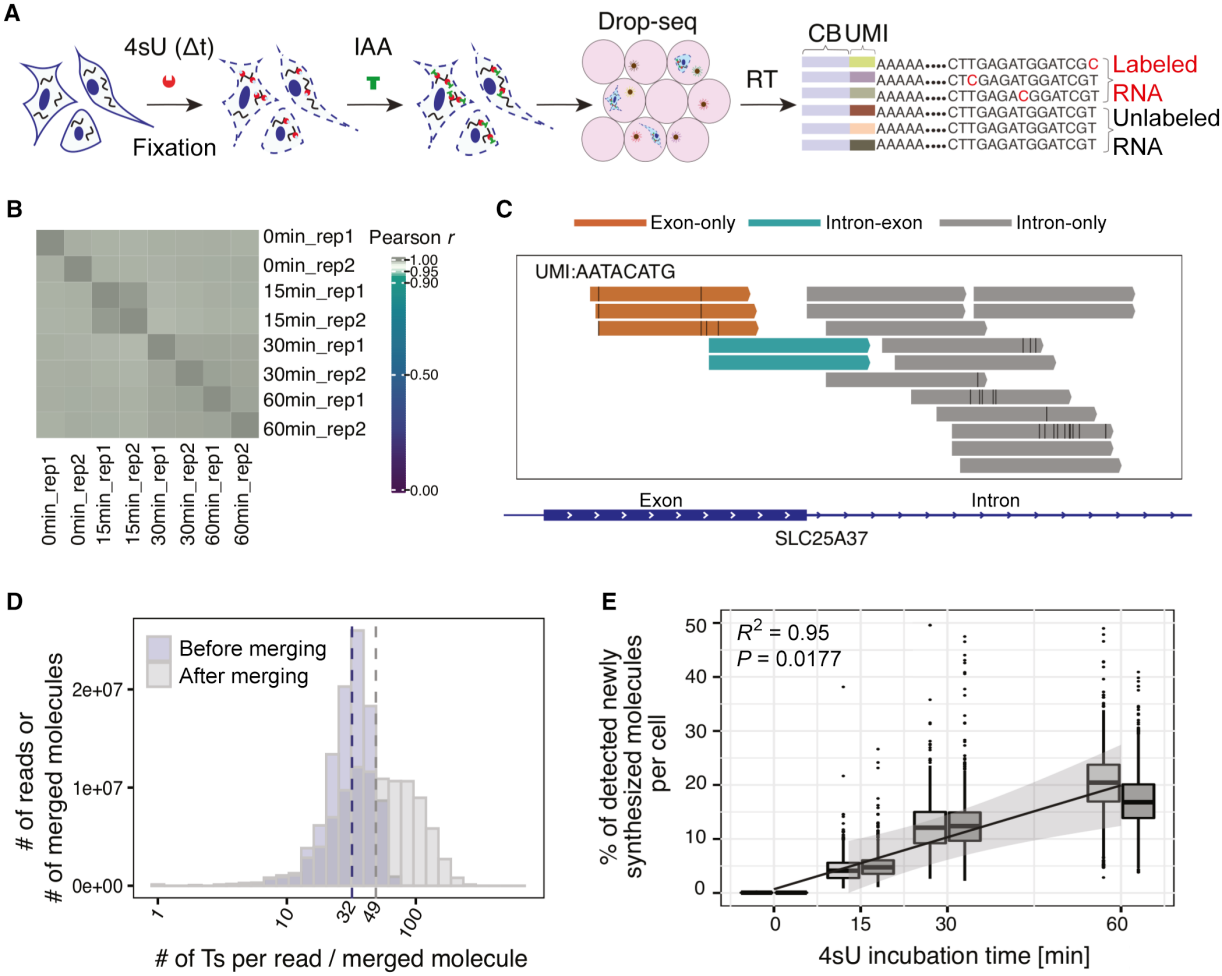
Newly synthesized and preexisting mRNAs in single cells are accurately detected with SLAM‐Drop‐seq by merging reads from the same UMI Schematic illustration of SLAM‐Drop‐seq experimental workflow. Briefly, cells were incubated with 4‐thiouridine (4sU) and fixed. 4sU residues in newly synthesized RNAs were alkylated by iodoacetamide (IAA) *in situ* in fixed cells. This resulted in T‐>C transitions during the reverse transcription step, after encapsulation of single cells in oil droplets. scRNA‐seq libraries were prepared after droplet lysis and the T‐>C conversions in barcoded reads were detected by computational analysis. CB = Cell barcode, UMI = Unique Molecular Identifier.Pseudo‐bulk gene expression levels are highly correlated between samples. Gene expression counts were summed across single cells in each sample and converted to counts per million (CPM). The log2(CPM + 1) values were used for the correlation analysis.Different parts of the same transcript are captured and sequenced in SLAM‐Drop‐seq data. Example Genome browser track shows reads from one single UMI in one cell are mapped to both intronic and exonic regions of the *SLC25A37* gene.The accuracy of identification of labeled transcripts increases after merging reads originated from the same transcript (UMI) as the observed number of thymidines (Ts) in each molecule increases. Data shown are from the 15 min 4sU labeled samples.The molecules detected as newly synthesized increase proportionally to the 4sU labeling time. The regression was performed with the cross‐replicate means per labeling time. The gray bands around the regression line display the 95% confidence intervals. The central band indicates the median value, while the lower and upper hinges of the boxes correspond to the 25^th^ and 75^th^ quantiles. The upper whisker extends from the hinge to the largest value no further than 1.5 * IQR from the hinge (where IQR is the inter‐quartile range). The lower whisker extends from the hinge to the smallest value at most 1.5 * IQR of the hinge. There are two biological replicates for each 4sU incubation time, which are denoted by the side‐by‐side box plots. The number of cells in each boxplot is shown in Appendix Fig S2A.

SLAM‐Drop‐seq combines 4‐thiouridine (4sU) metabolic labeling and iodoacetamide (IAA) alkylation inside fixed cells with droplet‐based scRNA‐seq. We optimized the conditions to alkylate 4sU‐labeled RNAs in methanol‐fixed cell suspensions to preserve high‐integrity RNAs (Appendix Figs S1A and B). For our SLAM‐Drop‐seq experiment, we incubated HEK293 cells with 4sU for different durations (0, 15, 30, 60 min of incubation, two biological replicates each). We alkylated fixed cells and sequenced their mRNA transcriptomes via a Drop‐seq protocol. The accumulation of T‐>C transitions was detected in sequence reads marked with cellular and molecular barcodes by computational analysis to quantify newly transcribed and preexisting transcripts of each gene in each single cell (Fig 1A).

We obtained a total of 7,280 single cells that expressed at least 200 genes (Appendix Fig S2A). Although the sequencing depths were different between sequencing batches (batch 1 contained all of the 0, 30 and 60 min 4sU samples, while batch 2 contained the 15 min 4sU samples only; Appendix Fig S2B), we detected a median of more than 6,400 transcripts (UMIs) and 2,800 genes per cell for all labeling conditions (Appendix Fig S2C and D). Pseudo‐bulk level gene expression (i.e., gene expression summed up across all single cells in each sample) correlation analysis showed high correlation coefficients among all SLAM‐Drop‐seq samples (Pearson *r* > 0.99; Fig 1B), indicating highly reproducible sequencing results and no apparent changes in gene expression in response to short 4sU incubations.

### Newly synthesized and preexisting RNA molecules are quantified for both precursor and mature mRNAs in single cells

To distinguish newly synthesized transcripts from preexisting ones, the 4sU labeling status for each transcript had to be determined. The identification of 4sU labeled RNA molecules relies on the identification of T‐>C transitions. However, single‐nucleotide polymorphisms (SNPs) and sequencing errors can confound the quantification of labeled RNAs. To remove SNPs, we mapped the sequenced reads to a HEK293 cell line‐specific reference genome, which we created based on public HEK293 genomic sequencing data (see Materials and Methods). After this, we still observed 3.1% transcripts containing T‐>C transitions in the no‐4sU labeled control (i.e., 0 min 4sU) samples (Appendix Fig S3A). We hypothesized that these transitions were mainly due to the unfiltered SNPs and sequencing errors. We further filtered SNP positions identified from the no‐4sU control samples (see Materials and Methods). Then the true T‐>C transitions were distinguished from sequencing errors using a Bayesian model (see Materials and Methods). The resulting T‐>C conversions detected in the no‐4sU labeled control samples dropped to 0.3% (Appendix Fig S3A).

The labeling status (labeled or unlabeled) of a read enabled us to distinguish between newly synthesized and preexisting transcripts. This classification is central to our analysis, but it is highly dependent on the number of 4sU nucleotides incorporated per molecule. In our experiments, the median number of thymines (Ts) per read (median length: 138 nucleotides) is around 32 (Appendix Fig S3B). Thus, it is possible that reads originated from newly synthesized transcripts are devoid of T‐>C conversions because it is known that even with long labeling times, at most one in 40 uridines is expected to be substituted by 4sU (Herzog *et al*, 2017; Jürges *et al*, 2018). To minimize the chance of such false negatives, we took advantage of unique molecular identifiers (UMIs) introduced by the SLAM‐Drop‐seq protocol to computationally merge the reads that mapped to the same transcript. Since reads associated with the same UMI can cover different parts of the same transcript due to random fragmentation and PCR amplification, merging reads from the same UMI recovers the full coverage of the sequencing data (Fig 1C). The *in silico* merged “molecules,” as we refer to them, generally contained sequence information for longer parts of the fragments (median length: 190 nucleotides; Appendix Fig S3B) and higher number of Ts (median: 49; Fig 1D), which increased the expected number of T‐>C transitions in the newly synthesized RNA molecules. To identify the newly synthesized transcripts lacking detectable T‐>C conversions by chance (increased likelihood with a lower number of Ts), we calculated the posterior probability of a molecule to be newly synthesized based on its T‐>C conversions and the number of Ts observed using a Bayesian model. To obtain the prior parameter to the model, we performed an extra experiment in which HEK293 cells were incubated with 4sU for 24 h to label all newly synthesized transcripts. With the assumption of a constant 4sU incorporation rate, we observed the number of T‐>C conversions in molecules with a given number of Ts following the expected Poisson distribution (Appendix Fig S3C). We applied the Bayesian model to calculate the probability for a given molecule to be newly synthesized based on its number of observed Ts and T‐>C conversions (Appendix Fig S3D, also see Materials and Methods). After these quantifications, we observed a linear increase of the newly synthesized RNA fractions with increasing time of 4sU incubation (Fig 1E), further arguing for the reliability of SLAM‐Drop‐seq, regarding the accurate quantification of newly synthesized and preexisting RNAs.

To be able to infer gene‐specific RNA kinetic rates of transcription, processing, and degradation, we not only distinguished between newly synthesized (i.e., labeled) and preexisting (i.e., unlabeled) transcripts but also classified each molecule as a precursor (i.e., unspliced) or mature (i.e., spliced) RNA. The transcripts were identified as unspliced or spliced based on the presence or absence of intron coverage (Appendix Fig S4A, see Materials and Methods). The fractions of unspliced molecules are independent of 4sU incubation times (Appendix Fig S4B), meaning that metabolic labeling does not perturb mRNA processing. Thus, SLAM‐Drop‐seq can distinguish and measure the products of mRNA synthesis and processing in parallel and in a reliable manner.

### Reconstruction of the cell cycle and modeling of cell cycle time‐dependent RNA kinetic rates

**Figure 2:**
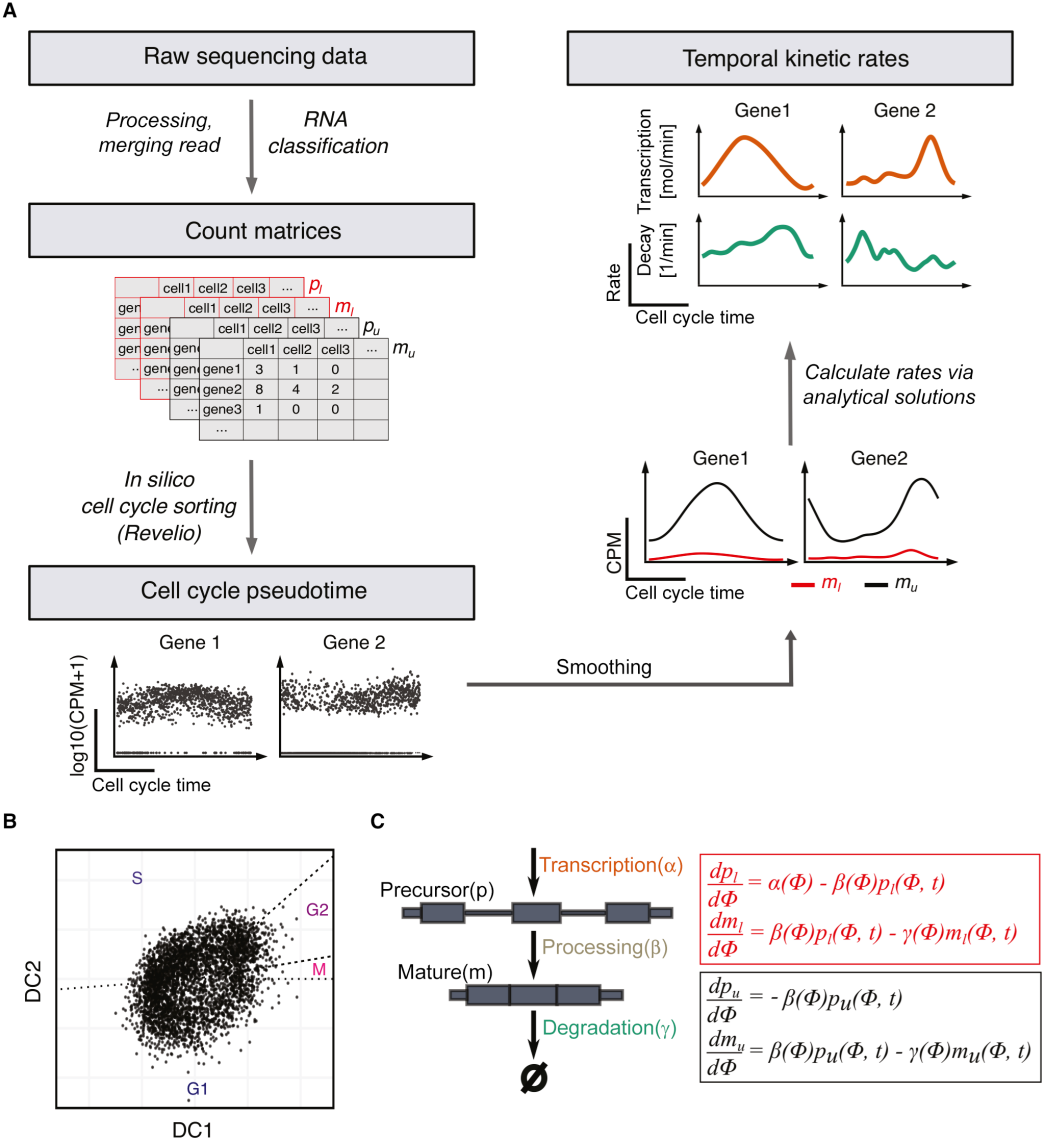
SLAM‐Drop‐seq of unsynchronized HEK293 cells reveals temporally regulated RNA kinetic rates throughout the cell cycle The main steps of the data processing pipeline and new algorithm Eskrate (Estimate time‐dependent RNA kinetic rates) for estimating time‐dependent RNA kinetic rates from SLAM‐Drop‐seq data. Step 1. Raw sequencing data is processed and reads from the same UMI (transcript) are merged. Different RNA types are classified (i.e., labeled precursor $p_{l},$labeled mature $m_{l}$, unlabeled precursors $\;p_{u}$ and unlabeled mature $m_{u}$) and gene expression counts are obtained for each RNA type. Step 2. After low quality cells are filtered, we use Revelio (Schwabe *et al*, 2020) to sort cells in cell cycle times. Single cell gene expression is transformed to gene expression over cell cycle time. Step 3. Time‐dependent RNA kinetic rates are calculated using Eskrate via the simplified analytical solutions of the kinetic rate model (see Materials and Methods).Based on gene expression, individual HEK293 cells were positioned along a circular trajectory representing the cell cycle using Revelio (Schwabe *et al*, 2020). DC = dynamic component. Shown are cells from the batch 1 experiments (0, 30 and 60 min 4sU samples).The mathematical model of RNA kinetic rates. The model consists of two mRNA maturation states (precursor and mature) and includes three kinetic rates: transcription *α*, processing *β* and degradation *γ*. Since maturation status and labeling status are independent events, we obtain four RNA types from the SLAM‐Drop‐seq experiment: $p_{l},\;m_{l},p_{u},$and $m_{u}$. We model the dynamics of RNA abundances for each RNA type over the cell cycle time by ODEs involving the kinetic rates. RNA abundances are measured at given cell cycle time Φand 4sU labeling time *t*.

From SLAM‐Drop‐seq data, we obtained gene expression count matrices for four types of mRNAs (i.e., “labeled precursor,” “labeled mature,” “unlabeled precursor,” and “unlabeled mature”). For the calculation of RNA kinetic rates in single cells, we developed Eskrate, an R package, integrating the single‐cell‐level measurements of newly synthesized and preexisting precursor RNAs as well as newly synthesized and preexisting mature RNAs to infer gene‐specific transcription, processing, and degradation rates as a function of time. The inputs to Eskrate are gene expression profiles of single cells of the aforementioned four types of RNA molecules measured by SLAM‐Drop‐seq and the biological times (e.g., cell cycle time) for every single cell (Fig 2A). We focus on the cell cycle as the first biological process to study the temporal dynamics of RNA kinetic rates, since gene expression is highly variable during the cell cycle and the underlying kinetic rates defining the expression of cycling genes are still not fully studied in unperturbed cells.

**Figure EV1:**
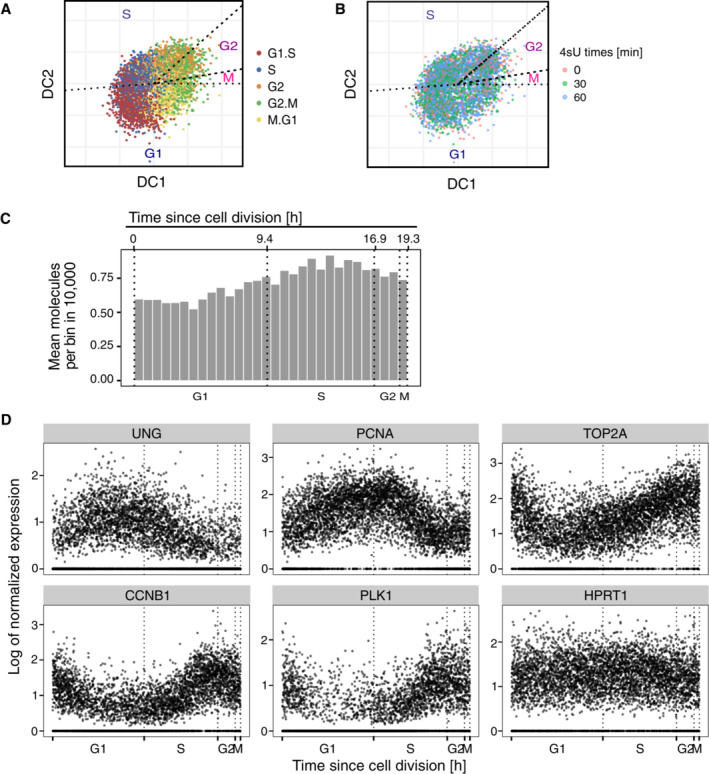
Asynchronous cells are sorted to a continuous cell cycle time using gene expression of single cells The two‐dimensional representation of the cells after dimensional reduction using Revelio (also see Fig 2A). Cells are pooled from batch 1 (i.e., 0, 30 and 60 min 4sU samples). Colors correspond to the cell cycle phase identities assigned using known cell cycle marker genes.Cells from different 4sU labeling times are randomly distributed within the cell clouds on the DC plot.The number of mean molecules per bin gradually increases along the cell cycle progression. Neighboring cells were grouped into 30 bins and the mean number of UMIs was used for the bar plots. The cells are the same ones as shown in (A) and (B).Representative gene expression profiles along the cell cycle (G1/S markers: UNG, PCNA; G2/M markers: CDK1, TOP2A, PKL1; House‐keeping gene: HPRT1). The cells are the same ones as shown in (D). Gene expression levels were normalized by dividing the raw counts by the total RNA contents per cell and then scaled with 2 × 10^4^.

To analyze kinetic rates along the cell cycle, the individual cells first have to be classified according to cell cycle stages. To this end, we used Revelio, a principal component analysis (PCA)‐based method (Schwabe *et al*, 2020), to reconstruct the cell cycle *in silico* from gene expression data of unsynchronized single cells. After filtering cells that showed more than 5% mitochondrial content, low expression of cycling genes, high expression of stress‐related genes, or high contents of ribosomal protein‐coding genes (see Materials and Methods), we analyzed the remaining cells using Revelio and obtained a two‐dimensional representation placing each cell along a circular trajectory corresponding to their progression through the cell cycle (Figs 2B and EV1A and B). Analogously to Schwabe *et al* (2020) we utilized known cell cycle phase durations (Cheng & Solomon, 2008) to assign cell cycle phase boundaries and ordered the cells along the cell cycle process based on their angle in the two‐dimensional space (Fig 2B). As expected, the mean captured RNA molecules per cell increased along the cell cycle progression (Fig EV1C). As an additional control, we compared the cell cycle time‐dependent expression profiles recovered by Revelio between well‐known cell cycle markers and housekeeping genes. While housekeeping genes such as HPRT1 show a constant expression profile along the cell cycle, the expression of well‐known cell cycle marker genes is upregulated at specific cell cycle phases that recapitulate their known expression patterns (Whitfield *et al*, 2002; Fig EV1D).

Based on the obtained cell cycle time‐resolved expression profiles of all four mRNA types, we implemented Eskrate by extending an RNA kinetic rate model commonly used for steady‐state analyses (Zeisel *et al*, 2011; Manno *et al*, 2018) to a system of four ordinary differential equations (ODEs) describing RNA abundance changes over the cell cycle time for the described four types of RNAs (Fig 2C). Importantly, we solved the time‐dependent versions of these equations such that all kinetic rates are (continuous) functions of the cell cycle time. In this way, we obtained equations directly: linking the time‐dependent abundances of the four RNA types to the time‐dependent rates of transcription, splicing, and degradation (see Materials and Methods). Due to the fact that we captured a low amount of precursor mRNAs (Appendix Fig S4B), we simplified the analytical solutions using approximations assuming that RNA processing (within minutes; Alpert *et al*, 2017) is much faster than RNA degradation (within hours; Murakawa *et al*, 2015; Schofield *et al*, 2018). We implemented Eskrate with simplified analytical solutions in which the time‐dependent transcription and degradation rates were driven by time courses of labeled and unlabeled mature RNAs (see Materials and Methods).

While the derived analytic solutions enable us to calculate kinetic rates from the experimental data, one drawback is that the ground truth is unknown. Therefore, as a proof‐of‐concept, we generated synthetic data assuming that both transcription and degradation rates are cell cycle time‐dependent (Appendix Fig S5A–C, also see Materials and Methods). We show that given a ground truth for kinetic rates, which generate certain expression profiles, the calculated kinetic rates accurately recapitulate the ground truths in all labeling cases using the full model (Appendix Fig S5D, see Materials and Methods). Using the simplified model, the kinetic rates are generally close to the ground truths as well. However, the rates calculated from longer 4sU labeling times are more precise (Appendix Fig S5D). Since we can only approximate the kinetic rates from the experimental data using the simplified model, in the following analyses, we show the results of kinetic rates in the cell cycle calculated from the 60 min 4sU labeled samples. Since it is theoretically possible that the same RNA levels result from different kinetic rate combinations, we additionally generated the same gene expression profile of mature RNA considering all possible kinetic regulation modes (i.e., constant transcription rate; constant degradation rate; and dynamic transcription and degradation rates). The calculated RNA kinetic rates recapitulate the simulated input transcription and degradation rates in all cases (Appendix Fig S6A). Therefore, both the full model and the simplified model are capable of distinguishing between gene expression patterns driven by changes in transcription compared to those driven by changes in degradation, even if both modes of regulation ultimately lead to the exact same time course of mature mRNA expression.

We calculate the transcription and degradation rates from the observed gene expression data (i.e., observation). Reversely, we can calculate the predicted gene expression (i.e., prediction) from the calculated kinetic rates (see Materials and Methods). The difference between the observation and the prediction can therefore be used as a measurement of the accuracy of the calculated rates. Using synthetic data, we show when the changes are small in transcription and degradation rates, the prediction changes linearly (or near linearly; Appendix Fig S6B and C). This analysis suggests that when errors are small in the calculated kinetic rates, the prediction should also contain small errors. In other words, the prediction should be close to the observation, if the calculated rates are close to the truth. Thus, we define genes as “well‐predicted” for which an estimation of transcription and degradation rates is meaningful if the difference between prediction and observation is small (see Materials and Methods).

### RNA transcription and degradation rates are dynamically regulated in the cell cycle

As explained above, we analyzed the kinetic rates in the cell cycle using the simplified model from the 60 min 4sU samples. Since single‐cell transcriptomic data are noisy, smoothed expression profiles along the cell cycle are used for the estimations (see Materials and Methods). To investigate the regulation of temporal RNA kinetic rates during the cell cycle, we only focused on genes whose expression oscillates along the cell cycle (i.e., cycling genes). The reliability of the smoothed profile for each gene is largely dependent on its dropout rate (Qiu, 2020). To avoid unreliable estimates from shallow gene expression data (Appendix Fig S7A), we down‐sampled the gene expression matrix and defined thresholds of dropout rates for cycling genes to consider the smoothed profiles reliable (Appendix Fig S7B–D, also see Materials and Methods). Applying these thresholds, we defined a core set of 399 cycling genes with confidence in their smoothed profiles.

**Figure 3:**
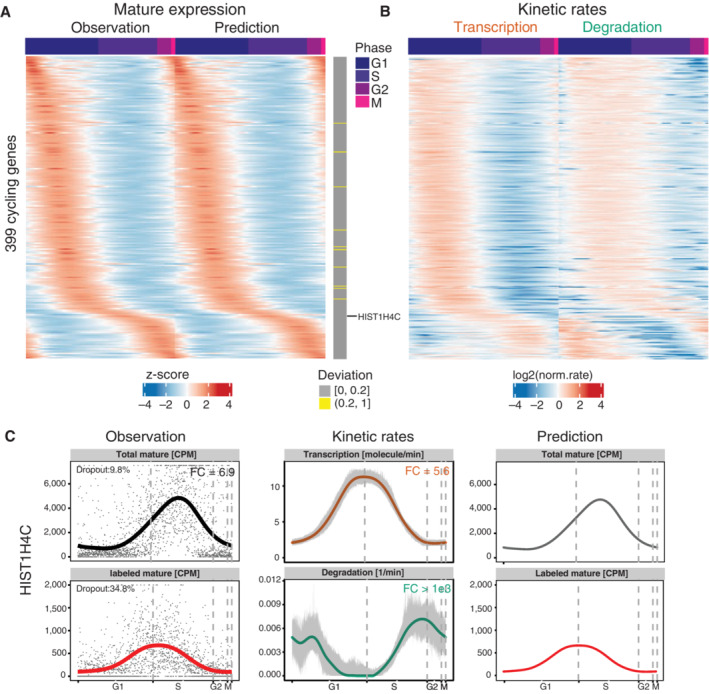
Gene expression and kinetic rates for cycling genes are highly dynamic along the cell cycle Gene expression of mature mRNAs for 399 cycling genes as quantified from sequencing data (i.e., observation) and as predicted (i.e., prediction) from calculated transcription and degradation rates along the cell cycle. The gray vertical bars on the right show the mean relative deviations between observation and prediction (see Materials and Methods). 389 out of those genes were identified as well‐predicted (deviation ≤ 0.2). Genes on rows were ordered by the peak time of the observation. Expression values were centered and scaled across cells for each gene.The calculated transcription and degradation rates for the genes shown in (A) along the cell cycle. The rates were normalized to their mean per gene and log2 transformed (transcription rates [molecules/min], degradation rates [1/min]).Profiles of the observed gene expression, the calculated kinetic rates and the corresponding predicted expression of *HIST1H4C* gene. Left panel: Observed gene expression levels (i.e., points) and their smoothed profiles (i.e., lines). CPM, counts per million. Total mature RNA is the sum of labeled and unlabeled mature RNAs. Middle panel: Kinetic rates calculated from the smoothed gene expression profiles are shown by the solid lines. The gray bands show the 90% confidence intervals (i.e., the 5–95% quantiles) of the rates calculated from bootstrapping (resampling of cells 100 times with replacement). Right panel: The predicted expression profiles of labeled and total mature RNAs that derived from the calculated rates.

The expression profiles of these 399 cycling genes peak at specific stages of the cell cycle (Fig 3A). We identified 97% of them to be well‐predicted by comparing gene‐wise the predictions to the observations (Fig 3A). The corresponding calculated transcription and degradation rates for cycling genes presented dynamic patterns as well along the cell cycle (Fig 3B), indicating temporal regulation of both processes during cell cycle progression. Noticeably, the peak times of transcription and degradation are less ordered with respect to the peak times in gene expression (Fig 3A and B), indicating that distinct regulation patterns for transcription and degradation can yield similar mRNA expression profiles.

As an example, of a dynamically regulated gene, we show the detailed profiles for the observed gene expression and the calculated kinetic rates along the cell cycle for the HIST1H4C gene (Fig 3C). It is a well‐known S‐phase enriched gene and its observed gene expression peaked exactly during the S phase in our data. Its transcription rate peaked in the S phase, while its degradation rate showed a sharp increase preceding the M phase. These results are highly consistent with the known kinetic regulation of replication‐dependent histone genes: in fact, it was previously reported that the upregulation of gene expression is mainly dependent on increased transcription, whereas the G2 phase associated decrease in detection is caused by decreased RNA stability (Harris *et al*, 1991).

**Figure EV2:**
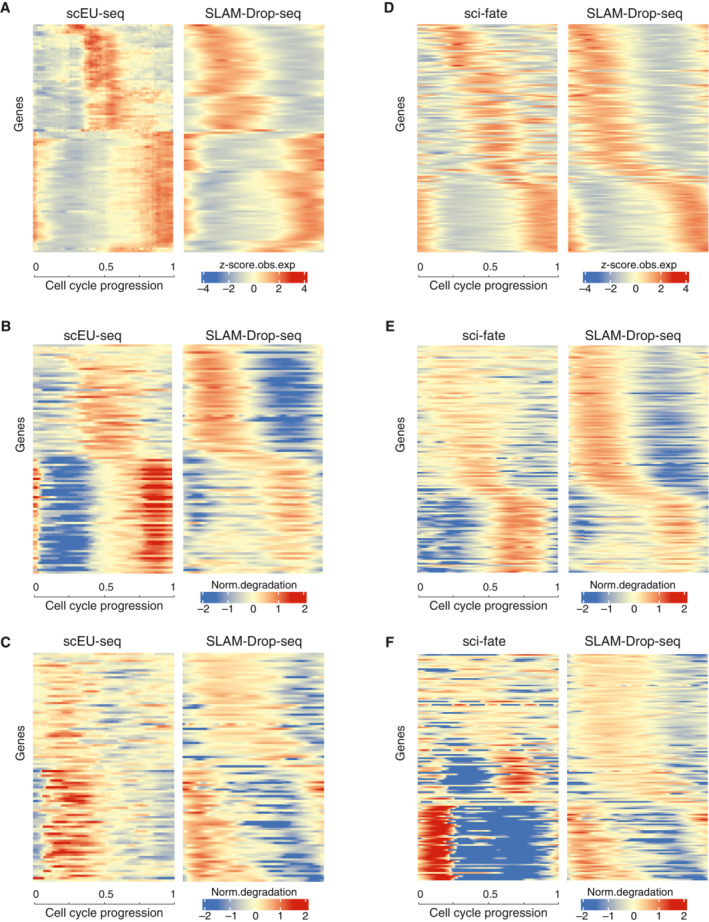
Time‐dependent RNA expression, synthesis and degradation rates comparison between SLAM‐Drop‐seq and scEU‐seq or sci‐fate Heatmaps of normalized z‐score for observed total mature RNA expression (obs. exp.) for 98 cell cycle variable genes shared in both scEU‐seq and SLAM‐Drop‐seq datasets. The scEU‐seq gene expression values of RPE1‐FUCCI cells are taken from sci‐fate paper Supplementary Table S1 (Battich *et al*, 2020). Adjacent SLAM‐Drop‐seq cells were grouped to match the 301 bins of the scEU‐seq data. Genes on the rows are ordered based on the positions of the maximum values of mean expression levels of the two datasets along the cell cycle. Binned cells (301) on the columns are ordered based on the assigned cell cycle pseudotimes.Heatmap representations of the transcription rates of genes shown in (A). scEU‐seq transcription rates are taken from Supplementary Table 1 (Battich *et al*, 2020). The SLAM‐Drop‐seq transcription rates were normalized to the mean and log transformed as the scEU‐seq data. Genes and cells are ordered in the same way as in (A).The normalized degradation rates are shown for the same genes and cells as shown in (A) and (B). scEU‐seq degradation rates are taken from Supplementary Table S1 (Battich *et al*, 2020). The SLAM‐Drop‐seq degradation rates were normalized to the mean and log transformed.Heatmaps of normalized z‐score for observed total mature RNA expression profiles for 140 overlapping cell cycle variable genes shared in both sci‐fate and SLAM‐Drop‐seq datasets. Raw sci‐fate sequencing data of A549 cells (Cao *et al*, 2020) were processed by following the sci‐fate method (see details in Materials and Methods). Genes on the rows are ordered based on the maximum values of the mean across both data sets. Expression values for adjacent cells along the cell cycle are averaged to have the same number of columns between the two datasets.Heatmap representations of normalized transcription rates (normalized to the mean and then log transformed) of genes shown in (D). Genes and cells are in the same orders as in D.Heatmap representations of normalized degradation rates (normalized to the mean and then log transformed) of genes shown in (D). Genes and cells are in the same orders as in (D).

To evaluate the robustness of our method and estimations, we compared the cell cycle time‐dependent profiles of RNA expression, synthesis, and degradation rates from our data with those of another cell line (RPE1‐FUCCI), obtained by using a different approach (scEU‐seq, Battich *et al*, 2020). The comparison of shared genes between the two datasets revealed a similar overall pattern of peaking times along the cell cycle, but with a relatively consistent time delay (Fig EV2A–C). HEK293 kinetics profiles peaked at an earlier cell cycle time with respect to the one calculated in the scEU‐seq paper. Interestingly, RPE1‐FUCCI cells seem to lack any transcriptional activity at the beginning of the cell cycle. While various reasons could account for these discrepancies, the genetic background, cell of origin, cell source, and ways of cell cycle sorting are likely contributors.

We also tested the reproducibility of our data and the reliability of the developed algorithm Eskrate by calculating the synthesis and degradation rates using the raw sequencing data from the published sci‐fate dataset (Cao *et al*, 2020). Specifically, we used the 4sU‐labeled and unstimulated A549 cell line sample to estimate the RNA kinetic rates. When compared to the 140 shared genes with HEK293 results, the two datasets exhibited a high degree of similarity (Fig EV2D–F). This finding not only confirms the reliability of the Eskrate algorithm by demonstrating its potential to analyze other datasets but also highlights the dynamic regulation of RNA kinetic rates in cell cycle variable genes across different human cell lines.

Furthermore, we averaged the calculated degradation rates and compared them to independent studies on RNA half‐lives in HEK293 and K562 cells calculated from constant degradation estimates (Murakawa *et al*, 2015; Schofield *et al*, 2018). We found that our estimates correlated with published data (Spearman correlation coefficients were 0.60 and 0.46, respectively; Appendix Fig S8A and B).

### Cycling genes exhibit different modes of kinetic regulation

**Figure 4:**
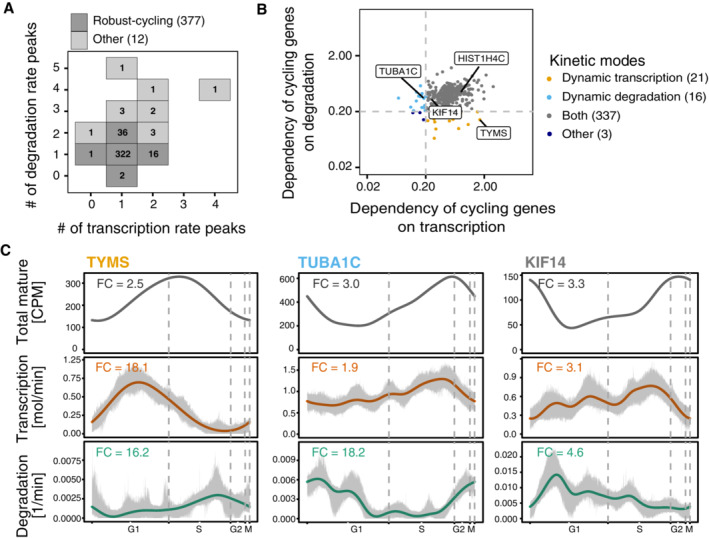
Cycling gene expression can result from various kinetic modes that often involve dynamically regulated RNA decay Identification of the robust‐cycling genes for kinetic rate regulation investigation. 377 out of the 389 well‐predicted cycling genes were defined as robust‐cycling genes by excluding those showing multiple transcription or degradation peaks.Cycling genes exhibit diverse kinetic modes. The kinetic modes were defined based on the dependency of dynamic transcription and dynamic degradation. The dependency on transcription (or degradation) is represented by the mean relative deviation between prediction from constant transcription (or degradation) and prediction from dynamic model (see Materials and Methods). ‘Both’ means genes are regulated by both dynamic transcription and dynamic degradation. Genes that were not identified into dynamic transcription, dynamic degradation or both were assigned into the “other” group. The labeled genes represent example cases in each mode.Profiles of the predicted expression, transcription and degradation rates along the cell cycle for example genes, which are labeled in (B) (dynamic transcription: TYMS; dynamic degradation: TUBA1C; dynamic transcription and dynamic degradation: KIF14). Gray areas around rates' profiles indicate the 90% confidence intervals calculated from bootstrapping (resampling of cells 100 times with replacement).

We wondered how frequent different modes of kinetic regulation are in human cycling genes and which kinetic parameters (transcription and/or degradation rate) drive the dynamic changes of gene expression. To this end, we filtered the well‐predicted cycling genes to exclude those that exhibit overly “wiggly” transcription or degradation profiles and obtained a set of 377 genes, which we called “robust‐cycling genes” (Fig 4A). We classified these genes according to their dependency on transcription and degradation dynamics by determining the predicted gene expression changes when kinetic rates are set constant. We hypothesized that forcing a constant transcription rate should lead to large deviations of the resulting expression profile from the observed one for genes temporally regulated by dynamic transcription. Analogously, for genes with their temporal expression patterns dominantly regulated by dynamic turnover, a constant degradation rate should cause large deviation in its predicted expression. Using this approach, we assigned the robust‐cycling genes to the following three major regulatory classes: (i) dynamic transcription, (ii) dynamic degradation; (iii) dynamic transcription and degradation (Fig 4B; Dataset EV1).

We found that the majority of the robust‐cycling genes (337 out of 377) depend on dynamic changes in both rates, implicating posttranscriptional regulation as an important regulatory mechanism for mRNA expression and oscillating gene expression during the cell cycle in HEK293 cells. Surprisingly, we only found 21 genes that achieve their cycling mRNA levels by dynamic transcription alone and 16 by dynamic changes in degradation rates (Fig 4B). As expected, when looking at the kinetic profiles without fixed parameter, genes classified as “dynamic transcription regulated” showed relatively constant degradation rate profiles throughout the cell cycle (e.g., TYMS in Fig 4C), while genes classified as “dynamic degradation regulated” exhibited roughly constant transcription rate profiles (e.g., TUBA1C in Fig 4C).

Our results show that expression of the majority of the cycling mRNAs is regulated at the level of transcription as well as degradation (Fig 4B). Examples for this mode of regulation are HIST1H4C (Fig 3C) and KIF14 mRNAs. KIF14 encodes a member of the kinesin‐3 superfamily of microtubule motor proteins, which is involved in chromosome segregation, mitotic spindle formation, and cytokinesis during the cell cycle (Carleton *et al*, 2006). KIF14 mRNA levels went up before G2 (Fig 4C), mostly due to increased transcription (Fig 4C). Toward the end of mitosis, the KIF14 transcript numbers quickly diminished due to a concomitant decrease in transcription and increase in degradation (Fig 4C).

To investigate how gene expression changes are defined by changes of transcription and degradation rates, we compared the peak times of both parameters for the 337 robust‐cycling genes. The lag times between the two rates come from a broad distribution (Appendix Fig S9A), indicating highly variable and gene‐specific coordination of mRNA kinetic rates. For example, mRNA expression profiles of SYNE2 and CLSPN genes peak during the G1 phase (Appendix Fig S9B). While their transcription rates are similar, their degradation rates have very different peak patterns. Overall, our findings demonstrate that our approach enables a description of transcription and degradation rates during the cell cycle with high temporal resolution.

## Discussion

In this study, we introduced SLAM‐Drop‐seq to distinguish newly synthesized and preexisting polyadenylated RNAs in single cells. We developed the R package Eskrate that enabled us to study temporal changes of RNA kinetic rates during the cell cycle based on SLAM‐Drop‐seq data. We inferred RNA kinetic rates in terms of cell cycle time from unsynchronized HEK293 cells and identified 377 robust‐cycling genes with confident rate estimates. We found the majority of cycling genes to be temporally regulated by changes in not only synthesis but also degradation rates. The temporal interplay between transcription and decay to achieve dynamic gene expression along the cell cycle revealed different modes of mRNA kinetic rates.

Previous studies on mRNA expression regulation throughout the cell cycle largely focused on transcriptional regulation (Cho *et al*, 2001; Liu *et al*, 2017). The underlying assumption of transcriptional control as a main regulatory mechanism is the basis of many methods to estimate RNA kinetic rates (Skinner *et al*, 2016). Here, we show that only a small fraction of cycling genes seems to be temporally regulated by transcription alone. Therefore, at least for cell cycle regulation in HEK293 cells, our data suggest that the generalization provided by our approach (time‐dependent rates with biophysical units) is not only of theoretical interest but also key in revealing the underlying biological processes: we found the majority of cycling genes to rely on some level of time‐dependent degradation in addition to their transcriptional regulation to achieve final expression patterns. In addition, we also identified 16 robust‐cycling genes with near‐constant transcription over the entire cell cycle that solely relied on dynamic degradation rates to modulate their expression (Fig 4B). A recent study showed that alternative polyadenylation site usage is highly regulated in cell‐cycle‐related genes at the single cell level, suggesting that posttranscriptional regulation is important for the regulation of gene expression during cell cycle progression (Wang *et al*, 2022). The switch of polyA sites of several cell cycle genes could potentially influence their turnover rate. Our study thus underscores the diverse temporal regulation types of transcription and degradation for defining gene expression in cell cycle progression. While the temporal changes of transcription and degradation rates have been previously revealed during the cell cycle (Eser *et al*, 2014; Battich *et al*, 2020), we infer these rates with a greater time resolution.

While this study was conducted, several approaches to measuring RNA half lives in single cells were published (Erhard *et al*, 2019; Hendriks *et al*, 2019; Battich *et al*, 2020; Cao *et al*, 2020; Qiu *et al*, 2020, 2022). NASC‐seq and scSLAM‐seq combined metabolic RNA labeling with the Smart‐seq2 approach (Picelli *et al*, 2013). While these methods have the advantage of full‐length coverage of RNA transcripts compared to Drop‐Seq (3′ end sequencing), the cell throughput is an order of magnitude smaller, capturing only a few hundred single cells (Erhard *et al*, 2019; Hendriks *et al*, 2019). scEU‐seq performed pull‐down to enrich for metabolically labeled RNAs, which is laborious, low‐throughput, and prone to introduce biases. Sci‐fate gained high sequencing throughput by using combinatorial indexing strategies (Cao *et al*, 2020). scNT‐seq adapted RNA metabolic labeling to droplet‐based sequencing approach, which effectively increases the throughput at a low cost (Qiu *et al*, 2020). Regarding the estimation of RNA kinetic rates, most of these studies (Erhard *et al*, 2019; Hendriks *et al*, 2019; Cao *et al*, 2020; Qiu *et al*, 2020) fit the degradation rates under a steady‐state assumption. Battich *et al* (2020) estimated the synthesis and degradation rates in the cell cycle with lower pseudotime resolution by pooling cells at close cell cycle times (Battich *et al*, 2020). One advanced computation tool was proposed by Qiu *et al* (2022), by which dynamical models and machine learning were used to predict dynamic cell transitions (Qiu *et al*, 2022). Compared to these studies, SLAM‐Drop‐seq obtains a high number of single cells with less labor at a lower cost. The 4sU labeled nucleotides are chemically converted *in situ* in fixed cells, which makes the method applicable for primary cells and complex tissues. Moreover, the kinetic model and algorithm we implemented take all kinetic rates as a function of time, meaning we are able to predict the absolute number of transcripts that are produced during a certain (cell cycle) time.

We are aware that the Drop‐seq‐based sequencing method has a 3′ bias, and this can lead to potential underestimation of unspliced RNA molecules (i.e., precursors). Nonetheless, due to the simplification of the mRNA kinetic model, the precursor mRNAs were not used for the kinetic rates calculation. Thus the 3′ bias of the sequencing should not influence the kinetic rates shown in this manuscript. However, full‐length scRNA‐seq methods might be better options to apply the full kinetic rate model if the number of single cells could scale to similar levels to Drop‐seq. Alternatively, when the accuracy of base calling for long‐read sequencing will match that of short read sequencing, their incorporation into the workflow will be a more reliable alternative.

Due to the low number of precursor RNA captured in our dataset (Appendix Fig S4B), we decided to adjust our model to increase the robustness of the transcription and degradation rates at the cost of not being able to investigate mRNA processing (i.e., splicing) rates for our application of SLAM‐Drop‐seq to cycling HEK293 cells. However, with deeper sequencing data, our mathematical framework should be capable of correctly estimating time‐dependent splicing rates as well.

## Materials and Methods

### Reagents and Tools table

**Table:** 

Reagent/Resource	Reference/ Source	Identifier/Catalog number
**Experimental models**		
HEK293 Flp‐In T‐Rex cells	Thermo Fisher Scientific	Cat # R78007
**Oligonucleotides**		
Read1CustSeqB	Macosko *et al* (2015)	GCCTGTCCGCGGAAGCAGTGGTATCAACGCAGAGTAC
TSO	Macosko *et al* (2015)	AAGCAGTGGTATCAACGCAGAGTGAATrGrGrG
SMART PCR oligo	Picelli *et al* (2014)	AAGCAGTGGTATCAACGCAGAGT
New‐P5‐SMART PCR hybrid oligo	Macosko *et al* (2015)	AATGATACGGCGACCACCGAGATCTACACGCCTGTCCGCGGAAGCAGTGGTATCAACGCAGAGT*A*C
**Chemicals, enzymes and other reagents**		
Dulbecco's modified Eagle medium	Gibco	Cat # 41965039
AMPure XP beads	Beckman Coulter	Cat # A63881
Bovine serum albumin (BSA)	Sigma‐Aldrich	Cat # A8806
Dithiothreitol (DTT)	Sigma‐Aldrich	Cat # D9779
DPBS	Gibco	Cat # 14190250
ECL detection reagent	GE Healthcare	Cat # RPN2209
Exonuclease I	New England Biolabs	Cat # M0293
Fetal bovine serum	Gibco	Cat # 10270106
Ficoll PM 400	Sigma‐Aldrich	Cat # F4375
l‐Glutamine	Gibco	Cat # 25030081
Iodoacetamide	Sigma‐Aldrich	Cat # I6125‐5G
Maxima H‐ RT enzyme	Thermo Fisher	Cat # EP0753
Methanol z. A. (min. 99.8%)	Th. Geyer	Cat # 11646935
MTSEA‐XX‐biotin	Biotium	Cat # 90066
Nylon membrane	Amersham Hybond‐N+	Cat # RPN203B
N‐Lauroylsarcosine (Sarkosyl)	Sigma‐Aldrich	Cat # L7414
Phase Lock Gel Heavy tubes	QuantaBio	Cat # 2302830
Phenol/Chloroform/Isoamylalkohol	Carl Roth	Cat # A156.1
QX200 Droplet Generation Oil	Bio‐Rad	Cat #1864006
SDS 20%	Carl Roth	Cat # 1057.1
Streptavidin‐HRP	Pierce	Cat # 21130
Superase•In RNAse Inhibitor	Thermo Fisher	Cat # AM2694
4‐Thio uridine	ChemGem	Cat # RP‐2304
Trizol	Thermo Fisher	Cat # 15‐596‐018
TrypLE express enzyme	Gibco	Cat # 12605036
**Software**		
bcl2fastq v2.20.0		
STAR v2.6.0a	Dobin *et al* (2013)	
bcftools v1.9	Li (2011)	
Drop‐seq tools v2.2.0	Macosko *et al* (2015)	
Revelio	Schwabe *et al* (2020)	
GATK toolkit	McKenna *et al* (2010)	
Seurat	Macosko *et al* (2015)	
DropletUtils	Griffiths *et al* (2018) and Lun *et al* (2019)	
FastQC v0.11.5		
Samtools v1.6		
Bedtools v2.30.0		
Pysam v 0.15.4		
VarScan v2.3.9		
sam2tsv.jar		
Python 2.7.18 & python 3.9.0		
R ‘mgcv’ packages	Wood (2017)	
R 3.6.0		
**Other**		
Nadia instrument	Dolomite Bio	
TC20 cell counter	Bio‐Rad	
Bio‐Dot apparatus	Bio‐Rad	
Direct‐zol RNA Miniprep	Zymo Research	
QuBit 2.0 Fluorometer	Thermo Fisher	
UV Stratalinker Ultraviolet Crosslinker	Stratagene	
Imager AI680	GE Amersham	
2100 Bioanalyzer Instrument	Agilent	
Nextera XT DNA Library Preparation Kit	Illumina	
Nextseq 500/550 HO v2 kit	Illumina	
Illumina Nextseq 500	Illumina	
Illumina NovaSeq 6000	Illumina	

### Methods and Protocols

#### Cell culture

Human HEK293 Flp‐In T‐Rex cells were cultured at 37°C with 5% CO_2_ and were grown in Dulbecco's Modified Eagle Medium supplemented with 10% fetal bovine serum and 2 mM l‐Glutamine. TrypLE Express Enzyme was used to detach cells and split them 2–3 times per week. The cell line was tested for mycoplasma contamination before experiments.

#### SLAM‐Drop‐seq protocol

- Split cells the day before the experiment in order to reach ~ 60/70% confluence the day after (exponential growth phase).
- Incubate cells with 300 μM 4sU for the desired amount of time.
- Wash cells without detaching from the plate with warm DPBS and dissociate them with TrypLE Express Enzyme for 1–2 min.
- Using cold DPBS, first wash cells in cold DPBS and then resuspend them at a final concentration of 1–2 million cells/ml (or lower).
  CRITICAL STEPS: be as quick as possible in this and previous steps, since transcription happening during this time will introduce unwanted signal to the results.
- Fix cells by gently adding drop by drop four volumes of cold methanol, with mild shaking of the dissociated single cells.
- Leave samples in fixation buffer (80% methanol, 20% DPBS) for at least 20 min at −20°C.
- Add IAA to final concentration of 10 mM to alkylate 4sU residues in cell suspensions. Same volume of fixation buffer was added to control samples.
- Alkylation reaction is performed overnight (16–18 h) in the dark with mild rotation at room temperature.
- Using the fixation buffer, wash cell samples once (300 *g*, 2′, 4°C) and resuspend them. Storage at −80°C is possible.
  SUGGESTED STEP: split the samples to control if the alkylation was efficient. Refer to the next section “Biotinylation blocking assay” to perform it.
- Rehydrate samples in rehydration buffer (DPBS, 0.01% BSA, 1:100 Superase · In RNAse Inhibitor).
- Quench the remaining IAA by adding 100 mM DTT (from freshly prepared 1 M DTT, dissolved in rehydration buffer) for 5 min at room temperature.
- Wash samples once in rehydration buffer and resuspend them in 400 μl DPBS ‐ BSA 0.01% + 1 U/μl Superase · In RNAse Inhibitor.
- Pass cell suspensions through a 40 μm cell strainer and count single cells (we used a TC20 automated cell counter).
- Proceed immediately to the chosen approach for single‐cell RNA library preparation.

N.B.: when working with 4sU and IAA reduce to the minimum light exposure, because both molecules are unstable and light‐sensitive.

#### Biotinylation blocking assay protocol

- After the overnight alkylation step, spin down a fraction of the samples (at least 50,000 cells) and dissolve it in TRIzol to check for alkylation efficiency.
- Extract the RNA following the TRIzol Reagent manual or using Direct‐zol purification columns.
- Biotinylate the purified RNA (same amount between samples) with MTSEA‐XX‐Biotin in biotinylation buffer (20 mM Tris–HCl pH 7.5, 1 mM EDTA, in ddH2O, 100 μl final volume) for more than 1 h in the dark at room temperature.
- Remove excess of MTSEA‐XX‐Biotin with Phenol/Chloroform (for RNA extraction) by using Phase Lock Gel Heavy tubes.
- Blot the samples to a nylon membrane (~ 100 μl) using a dot‐blot apparatus.
- Cross‐link the RNA to the membrane with 2,400 μJ UV_254nm_.
- Block the membrane in blocking solution (DPBS, 10% SDS, 1 mM EDTA) for 20 min at room temperature.
- Incubate the membrane with 1:10,000 dilution of 1 mg/ml streptavidin‐HRP in blocking solution for 10 min at room temperature.
- Wash the membrane six times with a blocking solution containing decreasing concentration of SDS (10, 1, 0.1%, applied twice each) for 10 min.
- Develop the biotin signal with ECL detection reagent and detect the chemiluminescence with an appropriate imager system.

#### Single‐cell library preparation and sequencing

Single cells were encapsulated using a Nadia Instrument and following the protocol from the manufacturer (version 1.8). 75,000 single cells in 250 μl rehydration buffer were encapsulated using 250 μl lysis buffer (6% Ficoll PM‐400, 0.2% Sarkosyl, 20 mM EDTA, 50 mM DTT, 200 mM Tris pH 7.5) and 3 ml QX200 Droplet Generation Oil. After encapsulation, the transcripts captured by barcoded beads were reverse transcribed (Maxima H‐ RT enzyme) and treated with exonuclease I following the instruction manual. The beads were than counted, and PCR amplification was run with SMART PCR primers (4,000 beads/sample, 6 reactions, 14 cycles). The PCR reactions were purified two times with 0.6 volumes of AMPure XP beads, quantified with QuBit, and analyzed on a Bioanalyzer DNA HS chip. 1,000 pg DNA libraries were used for the fragmentation and amplification steps (11 cycles) using the Nextera XT v2 DNA sample preparation kit. The libraries were double‐purified with 0.6 volumes of AMPure XP Beads, quantified, and pooled. The 15 min 4sU‐labeled samples were sequenced on a Illumina Nextseq500 sequencer (library concentration 1.8 pM; Nextseq 500/550 High Output v2 kit (150 cycles) in paired‐end mode; read 1 = 20/21 nt using the custom primer Read1CustSeqB (Macosko *et al*, 2015), read 2 = 133/132 nt). The 0, 30 and 60 min 4sU‐labeled samples were sequenced on a Illumina NovaSeq 6000 device (SP configuration, pair‐end; read 1 = 20 nt using the custom primer Read1CustSeqB (Macosko *et al*, 2015), index 1 (i7) = 8 nt, read 2 = 150 nt).

#### HEK293 cell line–specific reference genome creation

To increase mapping sensitivity and most importantly avoid mistaking single‐nucleotide polymorphisms (SNPs) from the minor allele in HEK293 cells as T‐>C conversions, a cell type‐–specific reference genome was created by amending the hg38 reference genome using SNPs detected from public bulk genome DNA (gDNA) sequencing data (SRA accession number: SRR2123657). To identify the SNPs in the HEK293 gDNA data, we mapped raw reads to the hg38 reference genome using STAR v2.6.0a (Dobin *et al*, 2013). SNPs were called using bcftools v1.9 (Li, 2011) “mpileup” and “call” functions. Then we filtered SNPs by the quality score using bcftools “view” function with parameter “%QUAL>20.” The generated SNP vcf file was then sorted to list chromosomes in lexicographic order. Very importantly, indels were filtered out to avoid changes in the length of the reference. Finally, the hg38 reference genome was corrected from the identified SNPs in HEK293 gDNA data using the “FastaAlternateReferenceMaker” function from the GATK toolkit (McKenna *et al*, 2010).

#### Data processing, mapping, splice status tagging, and mismatches tagging

Raw sequencing data were demultiplexed using bcl2fastq v2.20.0. The sequencing quality was checked using FastQC v0.11.5. Since we applied Drop‐seq for single‐cell RNA sequencing, we followed the Drop‐seq Core Computational Protocol (https://github.com/broadinstitute/Drop-seq) to process the data. Drop‐seq tool v2.2.0 (Macosko *et al*, 2015) was used to tag cell barcodes and molecular barcodes, to trim the 3′ poly(A) tail and potential 5’ SMART adapter sequences, and to filter out barcodes with low quality bases. The reads were then aligned to the HEK293 reference genome that we described above using STAR v2.6.0a (Dobin *et al*, 2013) with optional parameters “‐outFilterMismatchNmax 10 and –outSAMattributes AS NH nM NM MD” in order to write the MD tag, which carried the encoding mismatched and deleted reference bases into the aligned output files. Typically, around 80% of the reads mapped uniquely. Non‐uniquely mapped reads were not used for downstream analysis.

Gene annotation tags were added to the aligned reads by using the Drop‐seq tools v2.2.0 with filtered genome annotation file (GENCODE annotation release 29), which is described below. As we wanted to estimate kinetic rates for well‐annotated genes, we filtered the genome annotation file to keep well‐supported transcripts (transcript support level: 1, 2 and NA) for protein coding genes. For our interest of exploring other gene types, we also added “lincRNA” and “miRNA” from the genome annotation to our filtered gtf file. The Drop‐seq tools was further exploited to identify and correct potential barcode errors. The number of cells (cell barcodes associated with single‐cell transcriptomes) was determined by extracting the number of reads per cell, then plotting the cumulative distribution of reads against the cell barcodes ordered by descending number of reads and selecting the inflection point (‘knee’) of the distribution.

The gene annotation tags that were added by Drop‐seq Tools contained genes on both forward and reverse stands within the genomic location that the read mapped to. To annotate the specific gene name that the read mapped to, we further tagged the aligned reads with gene names stating the unique gene the read mapped to by considering the strands information and the genes that located at the same strand that the read mapped to.

In order to quantify mature and precursor RNAs for the kinetic rates modeling, we annotated the splice status for each uniquely aligned read with a tag stating the specific genomic location that the read mapped to (i.e., intron‐only, intron‐exon, exon‐only, exon‐exon, etc.) by intersecting the aligned read with gene annotation bed files containing intron and exon coordination information using bedtools “intersect” function. The bed files were generated from the gene annotation gtf file and split into intron, exon, and ambiguous bed files. To avoid ambiguous tagging where both intron and exon present at the read mapped locus, we annotated the ambiguous loci from the bed files and filtered them from the intron and exon bed files. The quantification of spliced and unspliced transcripts was described in later sections.

To count the T‐>C conversions, we need to find out T‐>C mismatches between the sequenced reads and the reference genome. We tagged each aligned read with a specific mismatch tag indicating the number of mismatches for all mismatch types using a python script that was adapted from “conversiontag” function in NASC‐seq analysis pipeline (Hendriks *et al*, 2019). This script utilized pysam (https://github.com/pysam-developers/pysam) to call single base mismatches by using the MD tags in conjunction with the CIGAR tags, which carried the mismatches, insertions, and deletions in the reads in the aligned files without requiring access to the entire original reference. Since the reads in SLAM‐Drop‐seq were not‐stranded, both sense and antisense transcripts were sequenced. As the reads were from the first strand of cDNA, T‐>C mismatches seen in reads mapped to the sense strand and A‐>G mismatches seen in reads mapped to the antisense strand were counted as T‐>C conversions. T‐>C conversions for each gene in each cell were counted by summing up the unique T‐>C positions in all reads that uniquely mapped to the gene in each single cell, which was described in the later section.

#### SNPs filtering

To reduce mistakes in T‐>C conversion quantification caused by SNPs, we mapped sequencing reads to the HEK293 specific reference genome as described in the mapping step above. To further get rid of SNPs that possibly presented in different cell passages, we checked the observed mismatches in no‐4sU labeled samples and identified mismatches in positions with high frequency (mismatch rate > 0.5 in position with reads depth ≥ 2) across all cells as SNPs. We then ignored these defined SNP positions when we count T‐>C conversions in all SLAM‐Drop‐seq samples.

#### Reads merging and transcript splice status identification

In SLAM‐Drop‐seq, unique molecular identifiers (UMIs) were used to identify different transcripts. The UMI tag was added to the first stand cDNA, which was PCR amplified and fragmented during library preparation. Since the fragmentation was random, different parts of each transcript with the same UMI were sequenced. To check the T‐>C conversion in each transcript (UMI), we merged all reads with the same UMI in each cell by collapsing the overlapping positions. Meanwhile, we checked each position in the merged sequence where at least one mismatch was observed and marked the read coverage and mismatch number at that position.

To identify the splice status of the sequenced transcript at the time that the 4sU labeling experiments ended, we checked the splice status tags of all reads that with the same UMI and identified the splice status for each transcript with the following logics: (i) If all reads from the transcript were mapped to exonic regions, the transcript was identified as “spliced”; (ii) If there was at least one read from the transcript were mapped to intronic regions, the transcript was identified as “unspliced”; (iii) Transcripts were defined as “ambiguous” if they fit neither of the above criteria, and they were not used for the kinetic estimation.

#### Newly synthesized and preexisting transcript quantification

To quantify the newly synthesized and preexisting transcripts, we need to identify labeling status from the T‐>C conversions observed in each transcript. As described above, reads that mapped to different genomic locations from the same transcript were merged. Thus, the number of T‐>C conversions for each transcript could be counted by summing up all the unique positions with a seen T‐>C conversion over reads that have the same UMI. This required the correct identification of T‐>C conversions in each position. However, the sequencing errors in high‐throughput sequencing could be confounded as T‐>C conversions. To distinguish the real T‐>C conversions from sequencing errors, we applied a Bayesian statistics method modeled as the mixture of two binomial distributions as described in the following formula:

(1) $$p_{c}\left(k_{l};n_{l},\varepsilon_{s},\rho \right)=\rho \cdot \text{Binom}\left(k_{l};n_{l},1-\varepsilon_{s}\right)+\left(1-\rho \right)\cdot \text{Binom}\left(n_{l}-k_{l};n_{l},\varepsilon_{s}\right)$$

where $p_{c}$ was the posterior probability of T‐>C conversion at locus l; $k_{l}$ was the number of reads supporting T‐>C conversions at locus l; $n_{l}$ was the total read coverage at locus l; $\varepsilon_{s}$ was the sequencing error rate in high‐throughput sequencing, which was approximated as 0.1% (Pfeiffer *et al*, 2018); ρ was the T‐>C conversion rate in all reads (prior probability), which was approximated by the ratio of observed number of confident T‐>C conversions (> 50% Ts were converted to Cs at each locus) to the observed number of Ts over all sequenced molecules for each sample. For each sequenced molecule (UMI), the total number of T‐>C conversion was calculated by summing up the posterior probabilities of T‐>C conversions over all positions within the molecule.

Due to inefficient 4sU incorporation, not all newly synthesized RNAs can be labeled with 4sU (Herzog *et al*, 2017). To recover the newly synthesized RNAs, which were not labeled, we applied a Bayesian statistics method with a mixture of Poisson and Binomial distributions. The method was described by the following function and terms:

(2) $$p_{\mathrm{new}}\left(k_{m};n_{m},\rho_{l},\varepsilon_{c},\theta \right)=\theta \cdot \text{Poisson}\left(k_{m};n_{m}\cdot \rho_{l}\right)+\left(1-\theta \right)\cdot \text{Binom}\left(k_{m};n_{m},\varepsilon_{c}\right)$$

where $p_{\mathrm{new}}$ was the posterior probability of molecule to be newly synthesized given the T‐>C conversions we observed in molecule m; $k_{m}$ was the number of T‐>C conversions in molecule m; $n_{m}$ was the total number of Ts in molecule m; $\varepsilon_{c}$ was the conversion error rate (false‐positive rate of T‐>C conversions), which was calculated from the no 4sU labeled cells ($\varepsilon_{c}$ = 0.00018); θ was the newly synthesized molecule fraction (prior probability), which was calculated for each gene in each sample by dividing the number of molecules with T‐>C conversions by the total number molecules observed; $\rho_{l}$ was the T‐>C incorporation rate constant in newly synthesized molecules, which was approximated by the mean T‐>C conversion rate (# of T‐>C divided by the # of Ts) over all precursor molecules in cells that were labeled with 4sU for 24 h. Under constant 4sU incorporation rate assumption, the number of T‐>C conversions in a newly synthesized molecule with a certain number of Ts should be a Poisson event. We did observe that the distribution of the fraction of T‐>C conversions recapitulated the density of Poisson distribution with the parameter $\rho_{l}*\#\mathrm{Ts}$ (Appendix Fig S3C). In the above equation, the Poisson distribution modeled the T‐>C conversion observed in the newly synthesized RNA molecules and the Binomial distribution modeled the T‐>C conversion in the preexisting RNA molecules. For each gene, the number of newly synthesized molecules was calculated by summing up the posterior probabilities $\left(p_{\mathrm{new}}\right)$ over all molecules.

#### Gene expression normalization, smoothing, and correlation analysis

Gene expression was normalized by dividing the raw counts to the total molecules captured in each cell and then multiplied with the scaling factor $10^{6}$ transforming the unit of our reads from absolute counts to counts per million (CPM). To get rid of the unwanted variabilities in single cells for kinetic rates estimation, we smoothed the gene expression profile for each gene along the cell cycle using penalized spline from the Generalized Additive Models (GAM; Wood, 2017) using R ‘mgcv’ packages (parameters used in the model were: bc=‘cc’, k = 20, gamma = 1.4).

To check the gene expression correlation between samples, the normalized gene expression (CPM) was averaged over all cells for each gene and log2 transformed with pseudocount 1. Pair‐wise gene expression correlations between different samples were calculated using the common set of genes across samples.

#### Cell filtering

We discarded cells that contained more than 5% mitochondrial RNA contents and clustered cells using Seurat UMAP algorithm (Macosko *et al*, 2015). Cells from batch 1 (0, 30, and 60 min 4sU samples) and batch 2 samples (15 min 4sU samples) were pooled and clustered separately. To focus the analysis on cells that were cycling, we measured the averaged expression of well‐known cell cycle marker genes and stress‐related genes for each cell cluster. The clusters that showed relatively low expression of cycling genes or relatively high expression of stress‐related genes were filtered out. We also removed cell clusters that showed relatively higher contents of ribosomal protein coding genes.

#### *In silico* cell cycle sorting

The sequenced HEK293 cells were unsynchronized. Thus, it is reasonable to assume that the single cells occupy one cell cycle uniformly. We sorted the filtered cells to a continuous cell cycle progression using the Revelio algorithm (Schwabe *et al*, 2020). It first identified the highly variable genes using similar method that defined in Seurat package (Macosko *et al*, 2015). By default, the variable genes are used for PCA. Here, we used the intersection of variable genes and the default variable genes that provided by Revelio package instead since we found it improved the cell cycle sorting. This algorithm then transformed high‐dimensional scRNA‐seq data of immortalized cell lines into a two‐dimensional circular trajectory with approximately uniform cell density in phase space. Due to the simplicity of the cell cycle signal, we approximated the cell cycle progression by the angular component from this two‐dimensional trajectory. This provided us with an ordering of the cells that follows the cell cycle progression. We then based our analysis on the time courses of individual genes that stemmed from this order of cells. With the help of estimated HEK293 cell cycle phase durations (Cheng & Solomon, 2008), we also estimated rough cell cycle phase boundaries and durations.

#### Time‐dependent RNA kinetic rate model

We develop a theoretical framework for estimating RNA kinetic rate based on a typical experimental setup for metabolic labeling. We first introduce a general model and its solutions for the dynamics of different mRNA types that includes the three rate parameters: transcription rate $\alpha \left(\Phi \right)$ in $\frac{\text{molecules}}{h}$, splicing rate $\beta \left(\Phi \right)$ in $\frac{1}{h}$ and degradation rate $\gamma \left(\Phi \right)$ in $\frac{1}{h}$, all depending on the cell cycle phase Φ. In contrast to RNA velocity (Manno *et al*, 2018), all three rates have units associated to them enabling absolute quantification rather than relative quantification. We also allow all three parameters to vary in time, enabling us to model the cell cycle as an example for a biological process.

We have previously introduced the distinction between precursor mRNA p and mature mRNA m and the fact that they can both be quantified by scRNA‐seq. The additional classification into labeled and unlabeled mRNA results in a total of four different types of mRNA (since both classifications are independent): unlabeled precursors $p_{u}$, unlabeled matures $m_{u}$, labeled precursors $p_{l}$, and labeled matures $m_{l}$. We note that the amounts of unlabeled precursors $p_{u}$ and labeled precursors $p_{l}$ sum up to yield the total amount of precursors p. The same is true for the mature mRNAs m. Therefore, the quantities $p_{u}$ and $p_{l}$ (as well as $m_{u}$ and $m_{l}$) signify a splitting up of the quantity p (and m) into two summands. This yields four linearly independent observables to infer three kinetic rates such that the problem is no longer under‐determined. We now proceed by modeling the concentration levels of the four different mRNA types in a gene‐specific manner across the cell population.

In Schwabe *et al*, 2020, it was observed that the cell cycle of immortalized cell lines is embedded as an approximately circular shape in two dimensions within the normalized gene expression space. For the modeling task at hand, we therefore simplify the cell cycle to a motion on a simple cycle in phase space. All cycles that are topologically equivalent to a circle allow for the definition of a phase variable to uniquely parametrize a position on the cycle. The position of a cell along the cell cycle is then uniquely defined by its cell cycle phase Φ and attains values between 0 and 2π.

The concentration levels of the four mRNA types $p_{u}$, $m_{u}$, $p_{l}$, $m_{l}$ depend on the position the cell attains within the cell cycle and the duration for which 4sU has been incorporated into the system. We hence choose to model these as functions of two variables: the cell cycle phase Φ with $\Phi \in \left[0,2\pi \right]$ and the labeling time t with $t\in {\mathbb{R}}_{0}^{+}$ and such that t=0 denotes the initiation of labeling. This yields the functions

$p_{u}-\text{unlabeled precursors}\;\left(\text{preexisting precursors}\right)$
$m_{u}-\text{unlabeled matures}\;\left(\text{preexisting matures}\right)$
$p_{l}-\text{labeled precursors}\;\left(\text{newly synthesized precursors}\right)$
$m_{l}-\text{labeled matures}\;\left(\text{newly synthesized matures}\right)$

These four variables are density fields on the phase range $\left[0,2\pi \right]$ changing in time and the two parameters Φ and t are independent. The densities given for t=0 can be considered the initial densities. We obtain the trajectory of an individual cell in phase space if we incorporate the time dependence of its position like

(3) $$\tilde{\Phi }\left(t\right)={\tilde{\Phi }}_{0}+\omega \cdot t$$

Here, ${\tilde{\Phi }}_{0}$ is the cell cycle phase the cell was in when labeling was initiated at t=0, and ω is the average velocity with which the cells move along the cell cycle. This is due to the fact that labeling time and cell cycle phase increase simultaneously in a linear way once a particular cell is chosen. For reasons of simplicity, we assume a typical cell cycle to take T=19.33 h (Cheng & Solomon, 2008) and set ω constant by defining $\omega =\frac{2\pi }{T}$. Taking the example of unlabeled precursors $p_{u}$, we can then describe the amount of unlabeled precursor mRNA molecules contained within an individual cell throughout the labeling experiment by moving along a curve $c^{p_{u}}\left(t\right)$ such that $c^{p_{u}}\left(t\right)≔p_{u}\left(\tilde{\Phi }\left(t\right),t\right)=p_{u}\left({\tilde{\Phi }}_{0}+\omega \cdot t,t\right)$ for t>0.

The parametrization $\left(\tilde{\Phi }\left(t\right),t\right)$ turns out to correspond to the characteristics of the partial differential equations (PDEs) of interest. This enables us to translate a system of PDEs to a system of ordinary differential equations (ODEs) in the process of finding solutions.

The total amounts of precursor RNA $p\left(\Phi \right)$ and the total amounts of mature RNA $m\left(\Phi \right)$ are defined by

(4) $$p\left(\Phi \right)≔p_{u}\left(\Phi ,t\right)+p_{l}\left(\Phi ,t\right),$$

(5) $$m\left(\Phi \right)≔m_{u}\left(\Phi ,t\right)+m_{l}\left(\Phi ,t\right).$$

Furthermore, at the initiation of labeling (t=0), there is no newly synthesized RNA yet, thus $p_{l}\left(\Phi ,0\right)=m_{l}\left(\Phi ,0\right)=0$ for all Φ. We conclude $p\left(\Phi \right)=p_{u}\left(\Phi ,0\right)$ and $m\left(\Phi \right)=m_{u}\left(\Phi ,0\right)$ for all Φ.

The functions $p_{u}$, $m_{u}$, $p_{l}$, $m_{l}$ describe distributions of molecules over the entire cell cycle. Without loss of generality and to simplify explanations, we concentrate our description on the unlabeled precursors $p_{u}$. Its distribution of molecules over the cell cycle is variable with respect to time t. Let $t_{1}$ and $t_{2}$ be two distinct time points with $0\leq t_{1}<t_{2}$. Two aspects govern the changes of the distribution from time $t_{1}$ to time $t_{2}$. On the one hand, all cells progress through the cycle. This would mean that the distribution of $p_{u}$ gets rotated along the cycle since the cells transport the density of $p_{u}$ with them. This part of the dynamics is termed advection and is given by $-\omega \frac{\partial p_{u}\left(\Phi ,t\right)}{\partial \Phi }$, which stems from the basic transport equation.

On the other hand, during the time $\Delta t≔t_{2}-t_{1},$ transcriptional changes occur and act on the distribution of $p_{u}$. In the case of unlabeled precursors, there is only splicing of already existing $p_{u}$ molecules occurring since no new unlabeled precursors are created for t≥0. The transcriptional changes during Δt are hence given by $-\beta \left(\Phi \right)\cdot p_{u}\left(\Phi ,t\right)$. This allows us to write down a PDE for the dynamics of $p_{u}$. It has the structure of a general balance equation for concentration changes, which is

(6) $$\begin{array}{c} \left(\text{local concentration changes}\right)=\left(\text{change}\;\mathrm{due}\;\text{to flux gradients}\right)\\ \;+\;\left(\text{change}\;\mathrm{due}\;\text{to local processes}\right). \end{array}$$

We obtain

(7) $$\frac{\partial p_{u}\left(\Phi ,t\right)}{\partial t}=-\omega \frac{\partial p_{u}\left(\Phi ,t\right)}{\partial \Phi }\;+\;\left(-\beta \left(\Phi \right)p_{u}\left(\Phi ,t\right)\right)$$

The initial condition (initial distribution) at time t=0 is given by $p\left(\Phi \right)$, since $p_{u}\left(\Phi ,0\right)=p\left(\Phi \right)$.

For the other mRNA types, we can argue very similarly and have to only adjust the transcriptional changes. The unlabeled matures $m_{u}$ gain the number of unlabeled precursors that is spliced during Δt but lose what is degraded of $m_{u}$. The labeled precursors $p_{l}$ are similar to the unlabeled precursors $p_{u}$ apart from the fact that transcription is a source for newly produced $p_{l}$. The dynamics for the labeled matures $m_{l}$ is analog to the dynamics of $m_{u}$.

We can derive a solution for the PDEs with their initial conditions with the help of the method of characteristics (Evans, 2010). We again demonstrate this only for $p_{u}$. We parametrize the variables Φ and t with a new variable τ and get

(8) $$\frac{dp_{u}\left(\tilde{\Phi }\left(\tau \right),\tilde{t}\left(\tau \right)\right)}{d\tau }=\frac{\partial p_{u}\left(\tilde{\Phi }\left(\tau \right),\tilde{t}\left(\tau \right)\right)}{\partial \Phi }\cdot \frac{d\tilde{\Phi }\left(\tau \right)}{d\tau }+\frac{\partial p_{u}\left(\tilde{\Phi }\left(\tau \right),\tilde{t}\left(\tau \right)\right)}{\mathrm{\partial t}}\cdot \frac{d\tilde{t}\left(\tau \right)}{d\tau }$$

via the chain rule. We now choose $\tilde{t}\left(\tau \right)$ such that

$$\frac{d\tilde{t}\left(\tau \right)}{d\tau }=1\Rightarrow \tilde{t}=\tau +c_{\tilde{t}}\Rightarrow \text{choose}\;c_{\tilde{t}}=0\Rightarrow \tilde{t}\left(\tau \right)=\tau \;\text{and rewrite}\;t=\tau .$$

We conclude that

(9) $$\frac{dp_{u}\left(\tilde{\Phi }\left(t\right),t\right)}{dt}=\frac{\partial p_{u}\left(\tilde{\Phi }\left(t\right),t\right)}{\partial \Phi }\cdot \frac{d\tilde{\Phi }\left(t\right)}{dt}+\frac{\partial p_{u}\left(\tilde{\Phi }\left(t\right),t\right)}{\mathrm{\partial t}}.$$

Now, we choose $\tilde{\Phi }\left(t\right)$ such that

$$\frac{d\tilde{\Phi }\left(t\right)}{dt}=\omega \Rightarrow \tilde{\Phi }\left(t\right)=\mathrm{\omega t}+c_{\tilde{\Phi }}\Rightarrow \text{choose}\;c_{\tilde{\Phi }}={\tilde{\Phi }}_{0}\Rightarrow \tilde{\Phi }\left(t\right)=\mathrm{\omega t}+{\tilde{\Phi }}_{0}.$$

This implies

(10) $$\frac{dp_{u}\left(\tilde{\Phi }\left(t\right),t\right)}{dt}=\omega \cdot \frac{\partial p_{u}\left(\tilde{\Phi }\left(t\right),t\right)}{\partial \Phi }+\frac{\partial p_{u}\left(\tilde{\Phi }\left(t\right),t\right)}{\mathrm{\partial t}}.$$

If we compare Equation (10) to the initial PDE (7), we can conclude:

(11) $$\frac{dp_{u}\left(\tilde{\Phi }\left(t\right),t\right)}{dt}=-\beta \left(\tilde{\Phi }\left(t\right)\right)p_{u}\left(\tilde{\Phi }\left(t\right),t\right).$$

which is an ODE for $p_{u}$ along a parametrization $\left(\tilde{\Phi }\left(t\right),t\right)$, the solution of which describes the trajectory of a cell in phase space. The initial condition becomes

(12) $$p_{u}\left(\tilde{\Phi }\left(0\right),0\right)=p\left({\tilde{\Phi }}_{0}\right).$$

We note that the parametrization for which the ODE in Equation (11) is satisfied coincides with the parametrization required for the trajectory of an individual cell (see Equation (3)). That means that the characteristics of the PDE are precisely the individual trajectories of cells.

We also observe that the time derivative along this characteristic is equal to the transcriptional changes from PDE in Equation (7). This enables us without further derivations to write down the ODEs for all other mRNA types along their characteristic curves. The corresponding ODEs yield

(13) $$\left\{\begin{array}{c} \frac{dp_{u}\left(\tilde{\Phi }\left(t\right),t\right)}{dt}=\;-\;\beta \left(\tilde{\Phi }\left(t\right)\right)\;p_{u}\left(\tilde{\Phi }\left(t\right),t\right),\;\\ \frac{dm_{u}\left(\tilde{\Phi }\left(t\right),t\right)}{dt}=\beta \left(\tilde{\Phi }\left(t\right)\right)\;p_{u}\left(\tilde{\Phi }\left(t\right),t\right)-\gamma \left(\tilde{\Phi }\left(t\right)\right)\;m_{u}\left(\tilde{\Phi }\left(t\right),t\right),\;\\ \frac{dp_{l}\left(\tilde{\Phi }\left(t\right),t\right)}{dt}=\;\alpha \left(\tilde{\Phi }\left(t\right)\right)\;-\beta \left(\tilde{\Phi }\left(t\right)\right)\;p_{l}\left(\tilde{\Phi }\left(t\right),t\right),\;\\ \frac{dm_{l}\left(\tilde{\Phi }\left(t\right),t\right)}{dt}=\beta \left(\tilde{\Phi }\left(t\right)\right)\;p_{l}\left(\tilde{\Phi }\left(t\right),t\right)\;-\gamma \left(\tilde{\Phi }\left(t\right)\right)\;m_{l}\left(\tilde{\Phi }\left(t\right),t\right).\; \end{array}\right.$$

with initial conditions

(14) $$\left\{\begin{array}{c} p_{u}\left(\tilde{\Phi }\left(0\right),0\right)=p\left({\tilde{\Phi }}_{0}\right),\\ m_{u}\left(\tilde{\Phi }\left(0\right),0\right)=m\left({\tilde{\Phi }}_{0}\right),\\ p_{l}\left(\tilde{\Phi }\left(0\right),0\right)=0,\;\\ m_{l}\left(\tilde{\Phi }\left(0\right),0\right)=0.\; \end{array}\right.$$

It can be shown that the following solutions indeed solve the system (13) as well as their corresponding PDE counterparts:

(15) $$\left\{\begin{array}{c} p_{u}\left(\Phi ,t\right)=p\left(\Phi -\mathrm{\omega t}\right)\cdot e^{-\frac{1}{\omega }\int_{\Phi -\mathrm{\omega t}}^{\Phi }\beta \left(\phi \right)\mathrm{d\phi}},\;\\ m_{u}\left(\Phi ,t\right)=p\left(\Phi -\mathrm{\omega t}\right)\cdot \frac{1}{\omega }\int_{\Phi -\mathrm{\omega t}}^{\Phi }\left[\beta \left(\phi \right)\cdot e^{-\frac{1}{\omega }\int_{\phi }^{\Phi }\gamma \left(\bar{\phi }\right)d\bar{\phi }-\frac{1}{\omega }\int_{\Phi -\mathrm{\omega t}}^{\phi }\beta \left(\bar{\phi }\right)d\bar{\phi }}\right]\mathrm{d\phi}\;\\ +m\left(\Phi -\mathrm{\omega t}\right)\cdot e^{-\frac{1}{\omega }\int_{\Phi -\mathrm{\omega t}}^{\Phi }\gamma \left(\phi \right)\mathrm{d\phi}},\;\\ \;p_{l}\left(\Phi ,t\right)=\frac{1}{\omega }\int_{\Phi -\mathrm{\omega t}}^{\Phi }\left[\alpha \left(\phi \right)\cdot e^{-\frac{1}{\omega }\int_{\phi }^{\Phi }\beta \left(\bar{\phi }\right)d\bar{\phi }}\right]\mathrm{d\phi},\;\\ m_{l}\left(\Phi ,t\right)=\frac{1}{\omega^{2}}\int_{\Phi -\mathrm{\omega t}}^{\Phi }\left[\beta \left(\phi \right)\cdot e^{-\frac{1}{\omega }\int_{\phi }^{\Phi }\gamma \left(\bar{\phi }\right)d\bar{\phi }}\cdot \int_{\Phi -\mathrm{\omega t}}^{\phi }\left[\alpha \left(\bar{\phi }\right)\cdot e^{-\frac{1}{\omega }\int_{\bar{\phi }}^{\phi }\beta \left(\bar{\phi }\right)d\bar{\phi }}\right]d\bar{\phi }\right]\mathrm{d\phi}. \end{array}\right.$$

With respect to the total precursor RNA $p\left(\Phi \right)$ and the total mature RNA $m\left(\Phi \right)$, we obtain the system

(16) $$\left\{\begin{array}{c} \frac{\mathrm{dp}\left(\Phi \right)}{d\Phi }=\;\frac{1}{\omega }\alpha \left(\Phi \right)\;-\frac{1}{\omega }\beta \left(\Phi \right)p\left(\Phi \right),\\ \frac{\mathrm{dm}\left(\Phi \right)}{d\Phi }=\frac{1}{\omega }\beta \left(\Phi \right)p\left(\Phi \right)-\frac{1}{\omega }\gamma \left(\Phi \right)m\left(\Phi \right), \end{array}\right.$$

with initial conditions $p\left(\Phi_{0}\right)≕p_{\Phi_{0}}$ and $m\left(\Phi_{0}\right)≕m_{\Phi_{0}}$. It can be shown that a solution for system (16) is given by

(17) $$\left\{\begin{array}{c} p\left(\Phi \right)=p_{\Phi_{0}}\cdot e^{-\frac{1}{\omega }\int_{\Phi_{0}}^{\Phi }\beta \left(\phi \right)\mathrm{d\phi}}\;\\ +\frac{1}{\omega }\int_{\Phi_{0}}^{\Phi }\left[\alpha \left(\phi \right)\cdot e^{-\frac{1}{\omega }\int_{\phi }^{\Phi }\beta \left(\bar{\phi }\right)d\bar{\phi }}\right]\mathrm{d\phi},\;\\ m\left(\Phi \right)=m_{\Phi_{0}}\cdot e^{-\frac{1}{\omega }\int_{\Phi_{0}}^{\Phi }\gamma \left(\phi \right)\mathrm{d\phi}}\;\\ +\frac{1}{\omega }\int_{\Phi_{0}}^{\Phi }\left[\beta \left(\phi \right)\cdot p\left(\phi \right)\cdot e^{-\frac{1}{\omega }\int_{\phi }^{\Phi }\gamma \left(\bar{\phi }\right)d\bar{\phi }}\right]\mathrm{d\phi}. \end{array}\right.$$

#### Solving the inverse problem

The solution formulas from the previous section state how the concentrations of the four mRNA types $p_{u}$, $m_{u}$, $p_{l}$, and $m_{l}$ can be calculated given dynamic rate parameters $\alpha \left(\Phi \right)$, $\beta \left(\Phi \right)$, and $\gamma \left(\Phi \right)$. However, in reality it is rather of interest to estimate the mRNA kinetic rates that caused the observable profiles for the different mRNA types. This is called an inverse problem.

Typical solutions to inverse problems concerning our model and structure of the previous solutions in system (15) would entail double integrals, which we cannot hope to solve analytically and which would be extremely unstable numerically.

We notice that the system of ODEs (13) contains the rate parameters $\alpha \left(\Phi \right)$, $\beta \left(\Phi \right)$, and $\gamma \left(\Phi \right)$ directly without involving integrals. This suggests a potential strategy for obtaining the rates directly from the dynamics. However, the ODEs describe the trajectory of a single cell throughout the labeling experiment. That means the observable data would have to consist of multiple measurements of the same cell at different labeling times. However, scRNA‐seq is not capable of such data.

Rather, we obtain measurements of multiple cells, which roughly cover the range of the cell cycle phase $\left[0,2\pi \right]$ but only for one single labeling time $t^{*}$. Hence, the observable data $p_{u}$, $m_{u}$, $p_{l}$, and $m_{l}$ are functions of the cell cycle phase Φ for fixed labeling times $t^{*}$. The numerical derivatives one can calculate from such observations coincide with the partial derivatives of $p_{u}$, $m_{u}$, $p_{l}$, and $m_{l}$ with respect to cell cycle phase Φ. What remains to be done is to investigate these partial derivatives to find out if they can be utilized to infer rate parameters.

We have calculated these partial derivatives. They are given by

(18) $$\left\{\begin{array}{c} \frac{\partial p_{u}\left(\Phi ,t\right)}{\partial \Phi }=\frac{1}{\omega }\alpha \left(\Phi -\mathrm{\omega t}\right)\frac{p_{u}\left(\Phi ,t\right)}{p\left(\Phi -\mathrm{\omega t}\right)}-\frac{1}{\omega }\beta \left(\Phi \right)p_{u}\left(\Phi ,t\right),\;\\ \frac{\partial m_{u}\left(\Phi ,t\right)}{\partial \Phi }=\frac{1}{\omega^{2}}\alpha \left(\Phi -\mathrm{\omega t}\right)\cdot \int_{\Phi -\mathrm{\omega t}}^{\Phi }\left[\beta \left(\phi \right)e^{-\frac{1}{\omega }\int_{\phi }^{\Phi }\gamma \left(\bar{\phi }\right)d\bar{\phi }-\frac{1}{\omega }\int_{\Phi -\mathrm{\omega t}}^{\phi }\beta \left(\bar{\phi }\right)d\bar{\phi }}\right]\mathrm{d\phi}\;\\ +\frac{1}{\omega }\beta \left(\Phi \right)p_{u}\left(\Phi ,t\right)-\frac{1}{\omega }\gamma \left(\Phi \right)m_{u}\left(\Phi ,t\right),\;\\ \frac{\partial p_{l}\left(\Phi ,t\right)}{\partial \Phi }=\frac{1}{\omega }\alpha \left(\Phi \right)-\frac{1}{\omega }\alpha \left(\Phi -\mathrm{\omega t}\right)\frac{p\left(\Phi \right)}{p\left(\Phi -\mathrm{\omega t}\right)}+\frac{1}{\omega }\alpha \left(\Phi -\mathrm{\omega t}\right)\frac{p_{l}\left(\Phi ,t\right)}{p\left(\Phi -\mathrm{\omega t}\right)}\;\\ -\frac{1}{\omega }\beta \left(\Phi \right)p_{l}\left(\Phi ,t\right),\;\\ \frac{\partial m_{l}\left(\Phi ,t\right)}{\partial \Phi }=-\frac{1}{\omega^{2}}\alpha \left(\Phi -\mathrm{\omega t}\right)\cdot \int_{\Phi -\mathrm{\omega t}}^{\Phi }\left[\beta \left(\phi \right)e^{-\frac{1}{\omega }\int_{\phi }^{\Phi }\gamma \left(\bar{\phi }\right)d\bar{\phi }-\frac{1}{\omega }\int_{\Phi -\mathrm{\omega t}}^{\phi }\beta \left(\bar{\phi }\right)d\bar{\phi }}\right]\mathrm{d\phi}\;\\ +\frac{1}{\omega }\beta \left(\Phi \right)p_{l}\left(\Phi ,t\right)-\frac{1}{\omega }\gamma \left(\Phi \right)m_{l}\left(\Phi ,t\right).\; \end{array}\right.$$

Additionally, the concentrations of the total precursor mRNAs p and mature mRNAs m are given by the sum of unlabeled and labeled amounts. The ODEs they obey are:

(19) $$\left\{\begin{array}{c} \frac{\mathrm{dp}\left(\Phi \right)}{d\Phi }=\;\frac{1}{\omega }\alpha \left(\Phi \right)\;-\frac{1}{\omega }\beta \left(\Phi \right)p\left(\Phi \right),\\ \frac{\mathrm{dm}\left(\Phi \right)}{d\Phi }=\frac{1}{\omega }\beta \left(\Phi \right)p\left(\Phi \right)-\frac{1}{\omega }\gamma \left(\Phi \right)m\left(\Phi \right). \end{array}\right.$$

Due to the fact that $p_{u}+p_{l}=p$ and $m_{u}+m_{l}=m$, we still only obtain four linearly independent equations.

Nevertheless, the six equations in (18) and (19) serve as a basis for inferring the rate parameters. The presence of an integral in the dynamics for $m_{u}$ and $m_{l}$ makes these equations unsuitable for obtaining unique solutions of $\alpha \left(\Phi \right)$, $\beta \left(\Phi \right)$, and $\gamma \left(\Phi \right)$. This leaves dynamics of m as the only one involving the dynamics of mature mRNA molecules. Out of the remaining three equations involving precursors, we choose two to form the following system from which we intend to obtain the dynamic rates $\alpha \left(\Phi \right)$, $\beta \left(\Phi \right)$ and $\gamma \left(\Phi \right)$:

(20) $$\left\{\begin{array}{c} \frac{\partial p_{u}\left(\Phi ,t\right)}{\partial \Phi }=\frac{1}{\omega }\alpha \left(\Phi -\mathrm{\omega t}\right)\frac{p_{u}\left(\Phi ,t\right)}{p\left(\Phi -\mathrm{\omega t}\right)}-\frac{1}{\omega }\beta \left(\Phi \right)p_{u}\left(\Phi ,t\right),\\ \frac{\mathrm{dp}\left(\Phi \right)}{d\Phi }=\frac{1}{\omega }\alpha \left(\Phi \right)-\frac{1}{\omega }\beta \left(\Phi \right)p\left(\Phi \right),\;\\ \frac{\mathrm{dm}\left(\Phi \right)}{d\Phi }=\frac{1}{\omega }\beta \left(\Phi \right)p\left(\Phi \right)-\frac{1}{\omega }\gamma \left(\Phi \right)m\left(\Phi \right).\; \end{array}\right.$$

The quantities $p\left(\Phi \right)$, $m\left(\Phi \right)$, and $p_{u}\left(\Phi ,t^{*}\right)$ for fixed labeling time $t^{*}$ are all measured within the labeling experiment. Since they cover the entire range of $\Phi \in \left[0,2\pi \right]$, we also know $p\left(\Phi -\omega t^{*}\right)$. The derivatives with respect to cell cycle phase can be numerically inferred in theory. Hence, we obtain a linear system (20) consisting of three equations with the three unknowns $\alpha \left(\Phi \right)$, $\beta \left(\Phi \right),$ and $\gamma \left(\Phi \right)$. This system has a unique solution, which is the analytical solution to the inverse problem.

In practice, current noise levels in scRNA‐seq experiments cannot be neglected. Our solution to the inverse problem can for now only be seen as a theoretical solution since noise levels in fact would have a huge impact on numerical derivation, making this approach currently infeasible. Due to the presence of the term $\alpha \left(\Phi -\mathrm{\omega t}\right)$, the above system should be solved by discretization. We would discretize over the entire interval $\left[0,2\pi \right]$ in order to solve the coupled system for all discretized time points at once. However, the equations in system (20) suggest a very weak coupling between the discretized variables, resulting in a very sparse parameter matrix. Such weak coupling of the parameter matrix is not capable of solving the issue of high noise terms in numerical derivation. Therefore, we suggest to reconsider this direct approach again once noise levels in scRNA‐seq have been substantially reduced making numerical derivation viable. For now, we choose a different approach for estimating the parameters of the mRNA kinetic rates.

#### General rate estimation for the full model

Instead of solving the inverse problem directly, we now consider the solutions from system (15) in order to infer the rate parameters $\alpha \left(\Phi \right)$, $\beta \left(\Phi \right),$ and $\gamma \left(\Phi \right)$. These analytic solutions involve integrals over the rate parameters, which we want to now remove from the equations. We consider that in case we have access to data with relatively short labeling times $0<t\ll T=\frac{2\pi }{\omega }$ (much shorter than the duration of the entire cell cycle), the cell cycle phase‐dependent changes of $\alpha \left(\Phi \right)$, $\beta \left(\Phi \right)$, and $\gamma \left(\Phi \right)$ in the interval $\left[\Phi -\mathrm{\omega t},\Phi \right]$ can be neglected. Then with the help of the mean value theorem, we conclude that approximations of the form

(21) $$\int_{\Phi -\mathrm{\omega t}}^{\Phi }f\left(y\right)\mathrm{dy}\;\approx \;\mathrm{\omega t}\cdot f\left(\Phi \right)$$

hold true for $\alpha \left(\Phi \right)$, $\beta \left(\Phi \right)$, $\gamma \left(\Phi \right)$. The solutions for $p_{u}$, $m_{u}$, $p_{l}$, and $m_{l}$ from equations (15) can then be approximated and written without integrals:

(22) $$\left\{\begin{array}{c} {\tilde{p}}_{u}\left(\Phi ,t\right)=p\left(\Phi -\mathrm{\omega t}\right)\cdot e^{-\mathrm{t\beta}\left(\Phi \right)},\;\\ {\tilde{m}}_{u}\left(\Phi ,t\right)=\frac{\beta \left(\Phi \right)p\left(\Phi -\mathrm{\omega t}\right)}{\gamma \left(\Phi \right)-\beta \left(\Phi \right)}\cdot e^{-\mathrm{t\beta}\left(\Phi \right)}+\left(m\left(\Phi -\mathrm{\omega t}\right)-\frac{\beta \left(\Phi \right)p\left(\Phi -\mathrm{\omega t}\right)}{\gamma \left(\Phi \right)-\beta \left(\Phi \right)}\right)\cdot e^{-\mathrm{t\gamma}\left(\Phi \right)},\;\\ {\tilde{p}}_{l}\left(\Phi ,t\right)=\frac{\alpha \left(\Phi \right)}{\beta \left(\Phi \right)}\cdot \left(1-e^{-\mathrm{t\beta}\left(\Phi \right)}\right),\;\\ {\tilde{m}}_{l}\left(\Phi ,t\right)=\frac{\alpha \left(\Phi \right)}{\gamma \left(\Phi \right)}-\frac{\alpha \left(\Phi \right)}{\gamma \left(\Phi \right)-\beta \left(\Phi \right)}\cdot e^{-\mathrm{t\beta}\left(\Phi \right)}+\frac{\alpha \left(\Phi \right)\beta \left(\Phi \right)}{\gamma \left(\Phi \right)\left(\gamma \left(\Phi \right)-\beta \left(\Phi \right)\right)}\cdot e^{-\mathrm{t\gamma}\left(\Phi \right)}.\; \end{array}\right.$$

During the experiment, we consider the short‐fixed labeling time $t^{*}=0.25h\ll 19.33h$. We measure $p_{u}\left(\Phi ,t^{*}\right)$, $m_{u}\left(\Phi ,t^{*}\right)$, $p_{l}\left(\Phi ,t^{*}\right)$, and $m_{l}\left(\Phi ,t^{*}\right)$ for all $\Phi \in \left[0,2\pi \right]$, and thus implicitly $p\left(\Phi -\omega t^{*}\right)$ and $m\left(\Phi -\omega t^{*}\right)$. Hence, we can solve the approximated solutions for $\alpha \left(\Phi \right)$ and $\beta \left(\Phi \right)$ directly:

(23) $$\left\{\begin{array}{c} \beta \left(\Phi \right)=-\frac{1}{t}\log\left(\frac{{\tilde{p}}_{u}\left(\Phi ,t^{*}\right)}{p\left(\Phi -\omega t^{*}\right)}\right),\;\\ \alpha \left(\Phi \right)=\frac{p\left(\Phi -\omega t^{*}\right)\cdot \beta \left(\Phi \right)\cdot {\tilde{p}}_{l}\left(\Phi ,t^{*}\right)}{p\left(\Phi -\omega t^{*}\right)-{\tilde{p}}_{u}\left(\Phi ,t^{*}\right)}.\; \end{array}\right.$$

A general solution for $\gamma \left(\Phi \right)$ involves solving the quadratic equation

(24) $$\begin{array}{c} 0=\gamma^{2}\left(\Phi \right)\\ -\beta \left(\varphi \right)\cdot \left(\frac{{\tilde{p}}_{l}\left(\Phi ,t^{*}\right)}{{\tilde{m}}_{l}\left(\Phi ,t^{*}\right)}+\frac{p\left(\Phi -\omega t^{*}\right)}{m\left(\Phi -\omega t^{*}\right)}+1\right)\cdot \gamma \left(\Phi \right)\\ +\beta^{2}\left(\Phi \right)\cdot \frac{{\tilde{p}}_{l}\left(\Phi ,t^{*}\right)}{{\tilde{m}}_{l}\left(\Phi ,t^{*}\right)}\cdot \frac{p\left(\Phi -\omega t^{*}\right)}{m\left(\Phi -\omega t^{*}\right)}\cdot \left(\frac{m\left(\Phi -\omega t^{*}\right)-{\tilde{m}}_{u}\left(\Phi ,t^{*}\right)}{p\left(\Phi -\omega t^{*}\right)-{\tilde{p}}_{u}\left(\Phi ,t^{*}\right)}\right). \end{array}$$

Its solution is a basic application of the quadratic solution formula:

(25) $$\begin{array}{c} \gamma_{1,2}\left(\Phi \right)=\frac{\beta \left(\Phi \right)}{2}\cdot \left(\frac{{\tilde{p}}_{l}\left(\Phi ,t^{*}\right)}{{\tilde{m}}_{l}\left(\Phi ,t^{*}\right)}+\frac{p\left(\Phi -\omega t^{*}\right)}{m\left(\Phi -\omega t^{*}\right)}+1\right)\\ \pm \frac{\beta \left(\Phi \right)}{2}\;\sqrt{{\left(\frac{{\tilde{p}}_{l}\left(\Phi ,t^{*}\right)}{{\tilde{m}}_{l}\left(\Phi ,t^{*}\right)}+\frac{p\left(\Phi -\omega t^{*}\right)}{m\left(\Phi -\omega t^{*}\right)}+1\right)}^{2}-4\frac{{\tilde{p}}_{l}\left(\Phi ,t^{*}\right)}{{\tilde{m}}_{l}\left(\Phi ,t^{*}\right)}\frac{p\left(\Phi -\omega t^{*}\right)}{m\left(\Phi -\omega t^{*}\right)}\left(\frac{m\left(\Phi -\omega t^{*}\right)-{\tilde{m}}_{u}\left(\Phi ,t^{*}\right)}{p\left(\Phi -\omega t^{*}\right)-{\tilde{p}}_{u}\left(\Phi ,t^{*}\right)}+1\right)} \end{array}$$

We have to again consider the issue of noise in scRNA‐seq data when applying these analytic solutions to currently available experimental data. We observe that the solutions for $\alpha \left(\Phi \right)$ and $\gamma \left(\Phi \right)$ in Equations (23), (25) depend on the splicing rate $\beta \left(\Phi \right)$. The solution for $\beta \left(\Phi \right)$ itself consists of the logarithm of the fraction between unlabeled precursors $p_{u}$ at the time of measurement Φ and the total precursors p at the time of labeling initiation $\left(\Phi -\mathrm{\omega t}\right)$. While the mature gene counts in RNA‐seq data are known to be subjected to large amounts of both technical and biological noise, the detection rate for precursors is even an order of magnitude lower. We therefore expect $\beta \left(\Phi \right)$ to be extremely sensitive given the formula in Equation (23). Due to the dependencies, any noise will be propagated to $\alpha \left(\Phi \right)$ and $\gamma \left(\Phi \right)$.

Steady‐state estimations of the splicing rate β also do not yield reliable results. In preliminary data from bulk RNA‐seq involving metabolic labeling, we found a huge dynamic range for the β‐estimates, spanning many orders of magnitude. Since there is no ground truth data, it is difficult to estimate the reliability of such data but due to the low data coverage, we are careful to trust such estimates. While our model might be useful for future experimental data with reduced noise levels, we now incorporate additional simplifications into the model in order to actually estimate a transcription rate $\alpha \left(\Phi \right)$ and a degradation rate $\gamma \left(\Phi \right)$ from currently available experimental data.

Since we introduce simplifications to our model in the next section, we from now on refer to the approximated rate parameters in Equations (23), (25) presented in this section as the estimates of the full model. The approximated rate parameters from the next section are then be referred to as the estimates of the simplified model.

#### Rate estimation for a simplified model

The splicing of transcripts (typically minutes) acts on a different time scale than the degradation (typically hours; Alpert *et al*, 2017). We therefore assume that $\gamma \left(\Phi \right)\ll$ $\beta \left(\Phi \right)$ and can also conclude $\frac{e^{-\mathrm{t\beta}\left(\Phi \right)}}{\beta \left(\Phi \right)}\ll$ $\frac{e^{-\mathrm{t\gamma}\left(\Phi \right)}}{\gamma \left(\Phi \right)}$. The approximation $e^{-\mathrm{t\beta}\left(\Phi \right)}\ll e^{-\mathrm{t\gamma}\left(\Phi \right)}$ introduces a larger error than the previous two simplifications but we find it reasonable in our setting as it severely simplifies the solution for ${\tilde{m}}_{u}\left(\Phi ,t\right)$. Incorporating these simplifications, we obtain:

(26) $$\left\{\begin{array}{c} {\overset{ˇ}{p}}_{u}\left(\Phi ,t\right)=p\left(\Phi -\mathrm{\omega t}\right)\cdot e^{-\mathrm{t\beta}\left(\Phi \right)},\;\\ {\overset{ˇ}{p}}_{u}\left(\Phi ,t\right)=\left(m\left(\Phi -\mathrm{\omega t}\right)+p\left(\Phi -\mathrm{\omega t}\right)\right)\cdot e^{-\mathrm{t\gamma}\left(\Phi \right)},\;\\ {\overset{ˇ}{p}}_{l}\left(\Phi ,t\right)=\frac{\alpha \left(\Phi \right)}{\beta \left(\Phi \right)}\cdot \left(1-e^{-\mathrm{t\beta}\left(\Phi \right)}\right),\;\\ {\overset{ˇ}{m}}_{l}\left(\Phi ,t\right)=\frac{\alpha \left(\Phi \right)}{\gamma \left(\Phi \right)}\cdot \left(1-e^{-\mathrm{t\gamma}\left(\Phi \right)}\right).\; \end{array}\right.$$

We note that the solutions concerning the mature RNAs are very similar to the solutions of the precursors, with the splicing rate $\beta \left(\Phi \right)$ and the degradation rate $\gamma \left(\Phi \right)$ interchanged. This is due to the fact that with our basic simplification that splicing is much faster than degradation, we are close to removing precursors from our system of equations and considering the system

(27) $$\left\{\begin{array}{c} \frac{dm_{u}\left(\tilde{\Phi }\left(t\right),t\right)}{dt}\;=\;-\gamma \left(\tilde{\Phi }\left(t\right)\right)\;m_{u}\left(\tilde{\Phi }\left(t\right),t\right),\;\\ \frac{dm_{l}\left(\tilde{\Phi }\left(t\right),t\right)}{dt}=\alpha \left(\tilde{\Phi }\left(t\right)\right)-\gamma \left(\tilde{\Phi }\left(t\right)\right)\;m_{l}\left(\tilde{\Phi }\left(t\right),t\right).\; \end{array}\right.$$

Since we find in our experimental data that the fraction of reads associated with precursors is on average less than 2% (Appendix Fig S4B), we do drop the precursors from the calculation of ${\tilde{m}}_{u}\left(\Phi ,t\right)$ and obtain the final approximations

(28) $$\left\{\begin{array}{c} {\bar{m}}_{u}\left(\Phi ,t\right)=m\left(\Phi -\mathrm{\omega t}\right)\cdot e^{-\mathrm{t\gamma}\left(\Phi \right)},\;\\ {\bar{m}}_{l}\left(\Phi ,t\right)=\frac{\alpha \left(\Phi \right)}{\gamma \left(\Phi \right)}\cdot \left(1-e^{-\mathrm{t\gamma}\left(\Phi \right)}\right).\; \end{array}\right.$$

We remark that starting from the simplified model (27) without precursors altogether leads to the exact same approximated solutions now mentioned and is a convenient verification for the validity of our complex solution (15) and the subsequent simplification steps. The approximations (28) can be solved for $\alpha \left(\Phi \right)$ and $\gamma \left(\Phi \right)$:

(29) $$\left\{\begin{array}{c} \gamma \left(\Phi \right)=-\frac{1}{t}\log\left(\frac{{\bar{m}}_{u}\left(\Phi ,t\right)}{m\left(\Phi -\mathrm{\omega t}\right)}\right),\;\\ \alpha \left(\Phi \right)=-\frac{1}{t}\cdot \frac{m\left(\Phi -\mathrm{\omega t}\right)\cdot {\bar{m}}_{l}\left(\Phi ,t\right)}{m\left(\Phi -\mathrm{\omega t}\right)-{\bar{m}}_{u}\left(\Phi ,t\right)}\cdot \log\left(\frac{{\bar{m}}_{u}\left(\Phi ,t\right)}{m\left(\Phi -\mathrm{\omega t}\right)}\right). \end{array}\right.$$

We have so far always done approximations on the side of the RNA concentration levels and utilized the true parameters $\alpha \left(\Phi \right)$ and $\gamma \left(\Phi \right)$. However, we do not know ${\bar{m}}_{u}$ or ${\bar{m}}_{l}$ but we have observations for $m_{u}$ and $m_{l}$ and want to take advantage of these values. Hence, we utilize the structure of (29) and define approximations $\hat{\alpha }\left(\Phi \right)$, $\hat{\gamma }\left(\Phi \right)$:

(30) $$\left\{\begin{array}{c} \hat{\gamma }\left(\Phi \right)=-\frac{1}{t}\log\left(\frac{m_{u}\left(\Phi ,t\right)}{m\left(\Phi -\mathrm{\omega t}\right)}\right),\;\\ \hat{\alpha }\left(\Phi \right)=-\frac{1}{t}\cdot \frac{m\left(\Phi -\mathrm{\omega t}\right)\cdot m_{l}\left(\Phi ,t\right)}{m\left(\Phi -\mathrm{\omega t}\right)-m_{u}\left(\Phi ,t\right)}\cdot \log\left(\frac{m_{u}\left(\Phi ,t\right)}{m\left(\Phi -\mathrm{\omega t}\right)}\right). \end{array}\right.$$

The approximations in (30) are the formulas implemented to obtain the results from the main text.

In order to get predictions from the calculated parameters, we utilize the exact solution formula to the simplified model (27) where precursors are removed. This leads us to the following prediction formulas:

(31) $$\left\{\begin{array}{c} {\hat{m}}_{u}\left(\Phi ,t\right)=m\left(\Phi -\mathrm{\omega t}\right)\cdot e^{-\frac{1}{\omega }\int_{\Phi -\mathrm{\omega t}}^{\Phi }\hat{\gamma }\left(\phi \right)\mathrm{d\phi}},\;\\ {\hat{m}}_{l}\left(\Phi ,t\right)=\frac{1}{\omega }\int_{\Phi -\mathrm{\omega t}}^{\Phi }\left[\hat{\alpha }\left(\phi \right)\cdot e^{-\frac{1}{\omega }\int_{\phi }^{\Phi }\hat{\gamma }\left(\bar{\phi }\right)d\bar{\phi }}\right]\mathrm{d\phi}. \end{array}\right.$$

We will later compare the differences between the observation $m_{u}$ and the prediction ${\hat{m}}_{u}$, as well as between $m_{l}$ and ${\hat{m}}_{l}$ and between their sums $m=m_{u}+m_{l}$ and $\hat{m}≔{\hat{m}}_{u}+{\hat{m}}_{l}$.

#### Simulating synthetic data

In experimental data the task is typically to calculate rate parameters from observed gene expression time courses. While the previously derived formulas enable us to calculate estimates for these rates, one drawback is that the ground truth is unknown. We therefore, as a proof‐of‐concept, show that given a ground truth for $\alpha \left(\Phi \right)$, $\beta \left(\Phi \right)$, and $\gamma \left(\Phi \right)$, which generate certain $p_{u}$, $m_{u}$, $p_{l}$, $m_{l}$, p, and m with the help of the formulas (15), we get reasonable rate parameters with our formulas for the full model from Equations (23), (25) and for the simplified model from Equation (30).

One issue in the formulas (17) is that we still need to choose initial values $p\left(\Phi_{0}\right)$ and $m\left(\Phi_{0}\right)$. This could for example be done via the steady‐state solutions $p\left(0\right)=\frac{\alpha \left(0\right)}{\beta \left(0\right)}$ and $m\left(0\right)=\frac{\alpha \left(0\right)}{\gamma \left(0\right)}$. However, after generating a time course over one cell cycle, we often observe that $p\left(0\right)\neq p\left(2\pi \right)$ and $m\left(0\right)\neq m\left(2\pi \right)$. Therefore, we continuously generate time courses for $p\left(\Phi \right)$ and $m\left(\Phi \right)$ over multiple periods (multiple cell cycles). We notice that the simulation converges after very few (fewer than 10) cycles such that $\left|p\left(\Phi \right)-p\left(\Phi +T\right)\right|<\varepsilon_{p}$ and $\left|m\left(\Phi \right)-m\left(\Phi +T\right)\right|<\varepsilon_{m}$. Hence, after our convergence criteria are met, we obtain the corresponding time courses $p\left(\Phi \right)$ and $m\left(\Phi \right)$ to a given input $\alpha \left(\Phi \right)$, $\beta \left(\Phi \right)$, $\gamma \left(\Phi \right)$. During the simulation of $p_{u}$, $m_{u}$, we then choose initial values $p\left(\Phi -\mathrm{\omega t}\right)$ and $m\left(\Phi -\mathrm{\omega t}\right)$ (see system (15)) from these converged time courses making them close to the true initial values and non‐arbitrary.

As a proof‐of‐concept for our model, we choose arbitrary time courses for $\alpha \left(\Phi \right)$, $\beta \left(\Phi \right)$, $\gamma \left(\Phi \right)$. With the help of the solution formulas from system (15), we then generate time courses for the different mRNA types given the dynamic parameters. Afterward, we plug these time courses into the formulas (23), (25) and then compare the resulting estimate for the full model to the ground truth. One example resulting from this strategy is depicted in Appendix Fig S5. We observe that the estimates for the rate parameters from our approximations (23), (25) of the full model recapitulate the ground truth very well.

We now choose a specific shape (such as a simple sine curve) that the time course $m\left(\Phi \right)$ should attain. We can then get different sets of parameters $\alpha \left(\Phi \right)$ and $\gamma \left(\Phi \right)$ that generate this time course. First, we set $\beta \left(\Phi \right)=\beta$ constant for the remainder of this section and assume that the parameter is known. Then, we consider the following three cases:

- Case I where γ is a constant and known parameter,
- Case II where α is a constant and known parameter,
- Case III where γ is a non‐constant but known parameter.

*Case I*: We know from (16)

(32) $$m^{\prime }\left(\;\Phi \right)=\frac{1}{\omega }\mathrm{\beta p}\left(\Phi \right)-\frac{1}{\omega }\mathrm{\gamma m}\left(\Phi \right)\Leftrightarrow p\left(\Phi \right)=\frac{\gamma }{\beta }m\left(\Phi \right)+\omega \frac{1}{\beta }m^{\prime }\left(\Phi \right).$$

We also have in (16)

(33) $$\begin{array}{c} p^{\prime }\left(\;\Phi \right)=\frac{1}{\omega }\alpha \left(\Phi \right)-\frac{1}{\omega }\mathrm{\beta p}\left(\Phi \right)\\ \Leftrightarrow \alpha \left(\Phi \right)=\omega p^{\prime }\left(\;\Phi \right)+\mathrm{\beta p}\left(\Phi \right)\;\\ \Rightarrow \alpha \left(\Phi \right)=\mathrm{\gamma m}\left(\Phi \right)+\omega \left(1+\frac{\gamma }{\beta }\right)m^{\prime }\left(\Phi \right)+\omega^{2}\frac{1}{\beta }m^{\prime \prime }\left(\Phi \right). \end{array}$$

Hence, choosing this alpha will generate the desired time course $m\left(\Phi \right)$.

*Case II*: When α and β are constant, we can find a simple solution for $p\left(\Phi \right)$ from (16):

(34) $$p^{\prime }\left(\;\Phi \right)=\frac{1}{\omega }\alpha -\frac{1}{\omega }\mathrm{\gamma p}\left(\Phi \right)\Rightarrow p\left(\Phi \right)=\frac{\alpha }{\beta }+\left(p_{0}-\frac{\alpha }{\beta }\right)e^{-\frac{1}{\omega }\beta \Phi }.$$

Coupled with the ODE for the total mature counts in (16), we obtain:

(35) $$\begin{array}{c} m^{\prime }\left(\;\Phi \right)=\frac{1}{\omega }\mathrm{\beta p}\left(\Phi \right)-\frac{1}{\omega }\gamma \left(\Phi \right)m\left(\Phi \right)\\ \Leftrightarrow \gamma \left(\Phi \right)=\beta \frac{p\left(\Phi \right)}{m\left(\Phi \right)}-\omega \frac{m^{\prime }\left(\Phi \right)}{m\left(\Phi \right)}\;\\ \Rightarrow \gamma \left(\Phi \right)=\frac{\alpha }{m}+\left(\beta \frac{p_{0}}{m}-\frac{\alpha }{m}\right)e^{-\frac{1}{\omega }\beta \Phi }-\omega \frac{m^{\prime }\left(\Phi \right)}{m\left(\Phi \right)}. \end{array}$$

For an appropriately chosen $p_{0}$, the calculated $\gamma \left(\Phi \right)$ will then generate the desired $m\left(\Phi \right)$.

*Case III*: We can extend case I and instead of choosing constant γ, we can choose any differentiable $\gamma \left(\Phi \right)$. Then from (16)

(36) $$m^{\prime }\left(\;\Phi \right)=\frac{1}{\omega }\mathrm{\beta p}\left(\Phi \right)-\frac{1}{\omega }\gamma \left(\Phi \right)m\left(\Phi \right)\Leftrightarrow p\left(\Phi \right)=\frac{1}{\beta }\gamma \left(\Phi \right)m\left(\Phi \right)+\omega \frac{1}{\beta }m^{\prime }\left(\Phi \right).$$

Analogously to previous considerations, we obtain

(37) $$\alpha \left(\Phi \right)=\left(\gamma \left(\Phi \right)+\omega \frac{1}{\beta }\gamma^{\prime }\left(\Phi \right)\right)m\left(\Phi \right)+\omega \left(1+\frac{1}{\beta }\gamma \left(\Phi \right)\right)m^{\prime }\left(\Phi \right)+\omega^{2}\frac{1}{\beta }m^{\prime \prime }\left(\Phi \right).$$

We now conclude that if we utilize the same $m\left(\Phi \right)$ in all three of these cases, we will have produced the same expression profile $m\left(\Phi \right),$ which is generated by three different types of regulation. We can show that our calculated parameter solutions from the previous sections can recapitulate these differences when we only provide the input $p_{u}$, $m_{u}$, $p_{l}$, $m_{l}$, p, and m (Appendix Fig S6A).

#### SLAM‐Drop‐seq data: Peak identification for gene expression and kinetic rates

To investigate the variability of gene expression and kinetic rates over the cell cycle, we implemented the peaking calling method to identify peak patterns of the profiles over cell cycle time. Since we smoothed the profiles by penalized splines, we can investigate their derivative in order to calculate the locations of local extrema. We defined the global fold change (*fc*) and the global difference (*diff*) of a profile as:

(38) $$\mathrm{fc}\left(\text{global}\right)=\frac{\max\left(\text{global}\right)}{\min\left(\text{global}\right)}$$

(39) $$\text{diff}\left(\text{global}\right)=\max\left(\text{global}\right)-\min\left(\text{global}\right)$$

If *fc* (*global*) of the profile was larger or equal to 1.5, it was considered as a peaking gene or peaking rate. For these peaking genes and peaking rates, we then checked all the remaining local maximums except the global maximum. If the difference between the local maximum and the larger value of the nearby local minimum was at least 1/4 of the *diff* (*global*), the local maximum was identified as a peak. The total number of peaks was counted by summing up all identified peaks in each profile. Genes with a single expression peak were identified as cycling genes.

#### Deviation calculation

To check how close the prediction (*pred*; gene expression that was calculated from calculated transcription and degradation rates) to the observation (*obs*; gene expression that was quantified from the sequencing data) is. We defined the mean absolute deviation (*dev*) from prediction to observation over all cells for each gene using the following equation where m is the cell number:

(40) $$\mathrm{dev}=\frac{1}{m}\cdot \sum_{i=1}^{m}\frac{\left|{\text{pred}}_{i}-{\text{obsv}}_{i}\right|}{{\text{obsv}}_{i}}$$

To identify well‐predicted genes that showed close prediction to observation, we checked the deviation values for all mature RNA types (labeled, unlabeled, and the total) and took the maximum value as the deviation for each gene.

To check how much prediction changed when transcription or degradation rate was set to a constant value, we calculated the deviation (*dev*_*c*_) from predictions that were calculated from a constant transcription or a constant degradation rate (*pred* (*constant rate*)) to the original predictions that were calculated from the dynamic model (*pred*) using the following equation where m is the number of cells across the cell cycle:

(41) $${\mathrm{dev}}_{c}=\frac{1}{m}\cdot \sum_{i=1}^{m}\frac{\left|\text{pred}{\left(\text{constant rate}\right)}_{i}-{\text{pred}}_{i}\right|}{{\text{pred}}_{i}}$$

The maximum of ${\mathrm{dev}}_{c}$ for all mature RNA types (labeled, unlabeled and the total) was taken as the deviation for each gene.

#### Comparison of scEU‐seq cell cycle–related data to SLAM‐Drop‐seq data

The gene expression and kinetic rates of RPE1‐FUCCI cells obtained using the scEU‐seq method are taken from the Supplementary Table S1 (Dataset from: Battich *et al*, 2020). The cell cycle procession time sorted gene expression (z‐score normalized), transcription rate and degradation rates (normalized to mean rate and log transformed) data are directly taken from the Supplementary Table S1 (Battich *et al*, 2020). To compare the kinetic rates and gene expression profiles between SLAM‐Drop‐seq and scEU‐seq, we took the intersection of cell cycle variable genes between the two datasets. The cell cycle time adjacent HEK293 cells from SLAM‐Drop‐seq were grouped into 301 bins to match the number of bins in the scEU‐seq data. Gene expression values and the kinetic rates were averaged in each bin for SLAM‐Drop‐seq data and normalized using the same normalization methods used in scEU‐seq data. The 98 common cell cycle variable genes between scEU‐seq and SLAM‐Drop‐seq data were compared using heatmaps (Fig EV2A–C).

#### Comparison of mRNA kinetic rates calculated from sci‐fate data to SLAM‐Drop‐seq data

The 4sU labeled dexamethasone (DEX) treated control (DEX 0 h) A549 samples from sci‐fate method (Cao *et al*, 2020) were used in this study. The A549 cells were labeled with 4sU for 2 h followed by IAA alkylation, which led to T‐>C transitions in the sequencing reads. Similar to SLAM‐Drop‐seq, both new and old RNAs can be identified in single cells simultaneously by identifying labeled RNAs based on T‐>C transitions in the reads.

The demultiplexed sci‐fate raw sequencing data of A549 cells were obtained directly from the authors (Cao *et al*, 2020). To obtain gene expression count matrices from the sequencing reads, the open‐source sci‐fate computational pipeline (https://github.com/JunyueC/sci-fate_analysis) for generating full expression and new expression gene counts were used. Same as the sci‐fate methods, the public ENCODE (ENCODE Project Consortium, 2004) A549 bulk RNA‐seq datasets (sample name: ENCFF542FVG, ENCFF538ZTA, ENCFF214JEZ, ENCFF629LOL, ENCFF149CJD, ENCFF006WNO, ENCFF828WTU, ENCFF380VGD) were used for SNP calling in generating gene expression matrix for new RNAs. In the end, gene counts for every gene in every cell were obtained for both total and new RNAs. We then grouped intronic and exonic reads and generated four RNA count matrices (i.e., labeled_mature, unlabeled_mature, labeled_precursor and unlbaled_precusor).

To sort the cells along the cell cycle, Reveilo was applied to the total (i.e., the sum of new and old) RNA count data of A549 cells. Prior to the cell cycle sorting, Seurat (Macosko *et al*, 2015) was used to cluster the cells and a sub cluster of cells that were not represented by any cell cycle marker genes were removed from the cell population. The remaining cells were ordered and assigned with cell cycle pseudo times using Revelio.

The classified gene expression count matrices (i.e., labeled_mature, unlabeled_mature, labeled_precursor and unlbaled_precusor) and the obtained cell cycle pseudo times were used to calculate transcription rates and degradation rates using Eskrate. Due to the sparseness of precursor counts in single cells, we also applied the simplified kinetic model to calculate the transcription and degradation rates. The calculated kinetic rates and the observed mRNA expression levels in A549 cells were then compared to the SLAM‐Drop‐seq data for the 140 shared cell cycle variable genes. To compare them gene‐by‐gene using heatmaps, the adjacent HEK293 cells were grouped to have equal number of cells as the sci‐fate data along the cell cycle (Fig EV2D–F). RNA expression levels and kinetic rates were normalized as data shown in Fig EV2A–D.

#### Mean RNA half‐lives calculation

RNA half‐lives were determined by RNA decay rates. To calculate the mean RNA half‐lives, we averaged the calculated time‐dependent RNA degradation rates over cells for each gene and then calculated the mean RNA half‐lives $t_{1/2}$ from the mean degradation rates ($\bar{\gamma }$) with the following equation:

(42) $$t_{1/2}=\frac{\ln\left(2\right)}{\bar{\gamma }}$$

#### Downsampling analysis to identify valid genes for rate estimation

Dropout event is one of the biggest challenges in scRNA‐seq data analysis as it leads to a large proportion of zeros in the gene expression matrix. The cell cycle time‐dependent transcription and degradation rates are dependent on the temporal profiles of labeled, unlabeled, and total mature RNAs (Equation (25) in Section 2.7.10). Thus, we need to consider the dropout rates of all three types of RNAs along the cell cycle progression time. Since scRNA‐seq data are noisy, the normalized gene expression data were smoothed before plugging them into the simplified analytical solutions to calculate the kinetic rates. To know which genes are valid for the rate estimation given the dropout rates, we need to check if the smoothed profiles are valid given the dropout rates. Since the dropout rate of the labeled mature RNAs are the highest, we downsampled the total mature RNA gene expression matrix of a list of selected genes to higher dropout rates that can represent the dropout rates of labeled mature RNAs. The selected genes composed both positive controls genes (i.e., known cell cycle marker genes, Whitfield *et al*, 2002) and negative control genes (i.e., known housekeeping genes for HEK293 cells, Hounkpe *et al*, 2021). They were down sampled to a series of fractions (i.e., 0.1, 0.2, 0.3, 0.4, 0.5, 0.6, 0.7, 0.8, 0.9, 1) using ‘DropletUtils’ (Griffiths *et al*, 2018; Lun *et al*, 2019). The downsampling was iterated 100 times for every gene at each downsampling fraction. Afterward, downsampled gene expression data was normalized and smoothed. Based on the fold changes in the smoothed profiles along the cell cycle, these genes were classified as marker genes if at least 95% of the iterative profiles showed a minimum fold change of 1.5. Otherwise, they were classified as housekeeping genes.

We binned genes to different groups based on their original fold changes (i.e., 0.5, 0.6, 0.7, 0.8, 0.9 and 1) in gene expression of the total mature RNAs. We counted the number of true positives, true negatives, false positives, and false negatives following the rules that shown in the following table:

**Table:** 

		*Original gene expression profiles*	
		Marker	Housekeeping
*Downsampled gene expression profiles*	Marker	True Positive (TP)	False Positive (FP)
	Housekeeping	False Negative (FN)	True Negative (TN)

(43) $$\text{True Positive Rate}\;\left(\mathrm{TPR}\right)=\frac{\mathrm{TP}}{\left(\mathrm{TP}+\mathrm{FN}\right)}$$

The confusion matrices were generated for marker genes in different groups based on their minimal fold changes. The true‐positive rates (Equation 43) were calculated and plotted against the dropout rate cutoffs (Appendix Fig S7D). Genes were expected to be valid for rate estimation if it had at least 95% true positives given its dropout rates.

## Author contributions

**Markus Landthaler:** Conceptualization; funding acquisition; project administration; writing – review and editing. **Haiyue Liu:** Software; formal analysis; investigation; methodology; writing – original draft; writing – review and editing. **Roberto Arsiè:** Methodology; writing – original draft; writing – review and editing. **Daniel Schwabe:** Software; investigation; methodology; writing – review and editing. **Marcel Schilling:** Formal analysis; methodology; writing – review and editing. **Igor Minia:** Methodology. **Jonathan Alles:** Methodology. **Anastasiya Boltengagen:** Methodology. **Christine Kocks:** Methodology. **Martin Falcke:** Formal analysis; supervision; writing – review and editing. **Nir Friedman:** Formal analysis. **Nikolaus Rajewsky:** Conceptualization; funding acquisition; project administration; writing – review and editing.

## Disclosure and competing interests statement

The authors declare that they have no conflicts of interest. NR is an editorial advisory board member. This has no bearing on the editorial consideration of this article for publication.

## Supporting information



Appendix

Expanded View Figures PDF

Dataset EV1

PDF+

## Data Availability

Single cell RNA sequencing: Gene Expression Omnibus GSE197667 (https://www.ncbi.nlm.nih.gov/geo/query/acc.cgi?acc=GSE197667). Visualization of cycling gene expression and kinetic rates: MDC (https://shiny.mdc-berlin.de/slam_drop_seq/). Data processing code: GitHub (https://github.com/rajewsky-lab/SLAM-Drop-seq). Eskrate R package: GitHub (https://github.com/rajewsky-lab/Eskrate).
