# Supplementary material for: SLAM‐Drop‐seq reveals mRNA kinetic rates throughout the cell cycle
Source: Mol Syst Biol. 2023 Aug 28;19(10):e11427. doi: 10.15252/msb.202211427 (PMC10568207; doi:10.15252/msb.202211427)
Supplement: Supplementary file 1 — Appendix [file MSB-19-e11427-s003.pdf]

APPENDIX

Table of content

Appendix Figure S1 2

Appendix Figure S2 3

Appendix Figure S3 4

Appendix Figure S4 5

Appendix Figure S5 6

Appendix Figure S6. 8

Appendix Figure S7 10

Appendix Figure S8 11

Appendix Figure S9 12

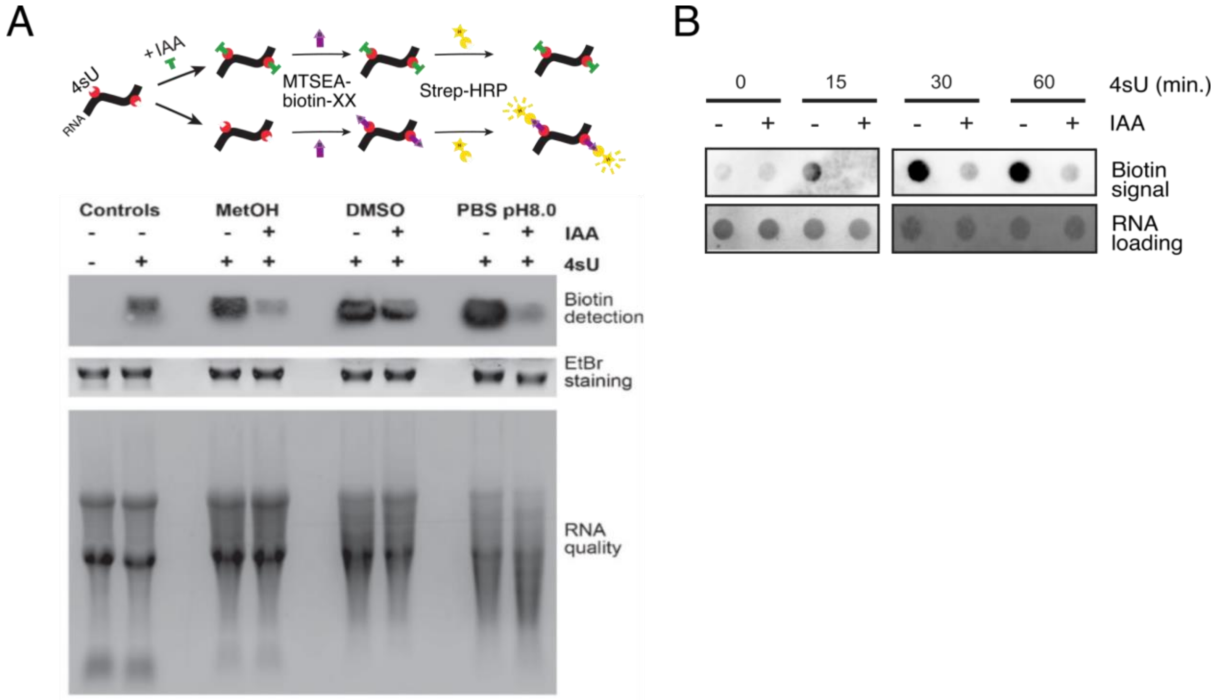

Appendix Figure S1. Fixation and IAA-alkylation of 4sU-labeled cells lead to a specific and time-dependent accumulation of T->C conversions.

(A) RNA blot showing efficiencies of 4sU-labeled RNA alkylation by IAA in different buffers. Upper panel: scheme of ‘biotinylation blocking assay’, which detects the efficiency of 4sU incorporation and IAA-alkylation. Briefly, the alkylation reagent MTS-biotin-XX is incubated with 4sU containing RNA which was or wasn’t previously exposed to IAA. If IAA alkylation occurred efficiently, MTS-biotin-XX cannot form covalent bonds with free thiol groups on the RNA. Lower panel: after 4sU incubation and fixation, cells were resuspended in the indicated solution (methanol, DMSO, PBS and with/without IAA), RNA was extracted and a biotinylation blocking assay performed (see upper scheme). Ethidium bromide staining for loading control and RNA gel for RNA quality checking. Note how in methanol the alkylation is efficient and the RNA is not degraded (low molecular weight RNAs still visible) (B) 4sU-labeled RNAs are efficiently alkylated by IAA following the SLAM-Drop-seq protocol. Dot blot representation of biotinylation blocking assay of HEK293 following the SLAM-Drop-seq protocol with different times of 4sU incubation. The decrease of biotin signal intensity for IAA incubated samples represents an efficient alkylation of IAA of 4sU labeled RNA. Staining of total RNA blotted with methylene blue as a loading control.

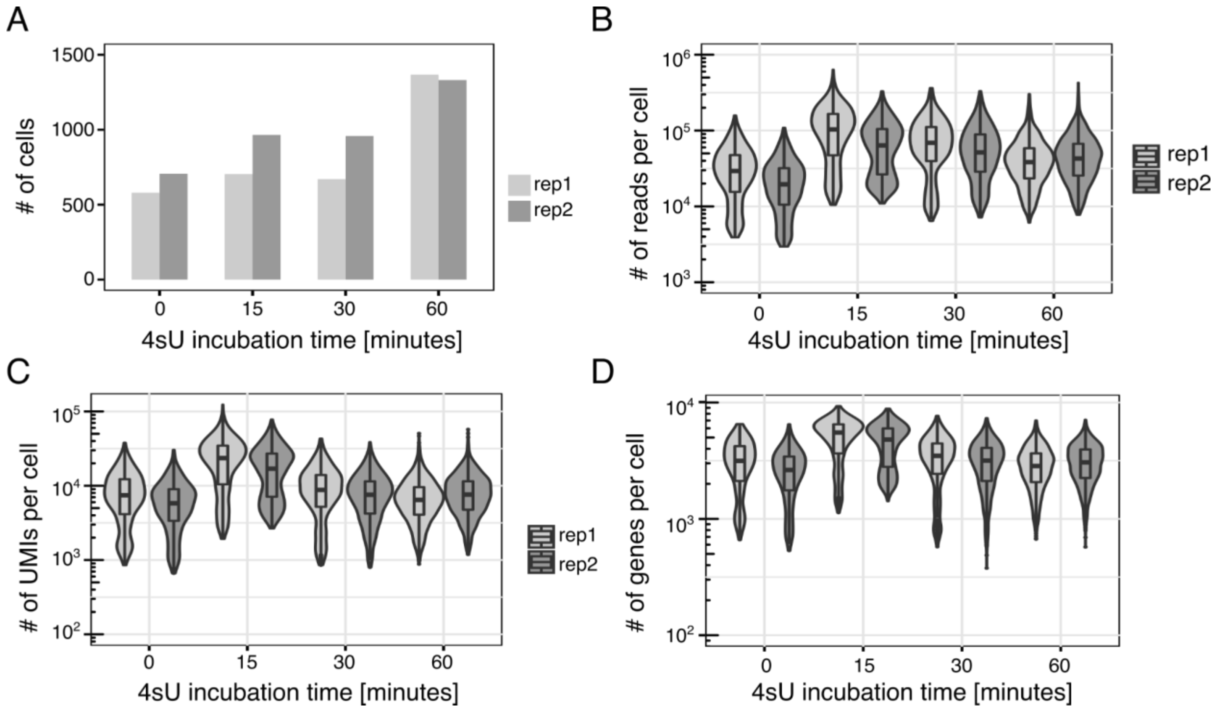

Appendix Figure S2. The sequencing statistics of SLAM-Drop-seq data.

(A) Number of cells detected in each sequencing library. Cells expressing less than 200 genes were excluded from the data. (B) The distribution of the sequencing depths in each sample is shown by the number of sequenced reads per cell. (C) The distribution of the number of UMIs (transcripts) detected per cell in each sample. (D) The distribution of the number of genes detected per cell in each sample.

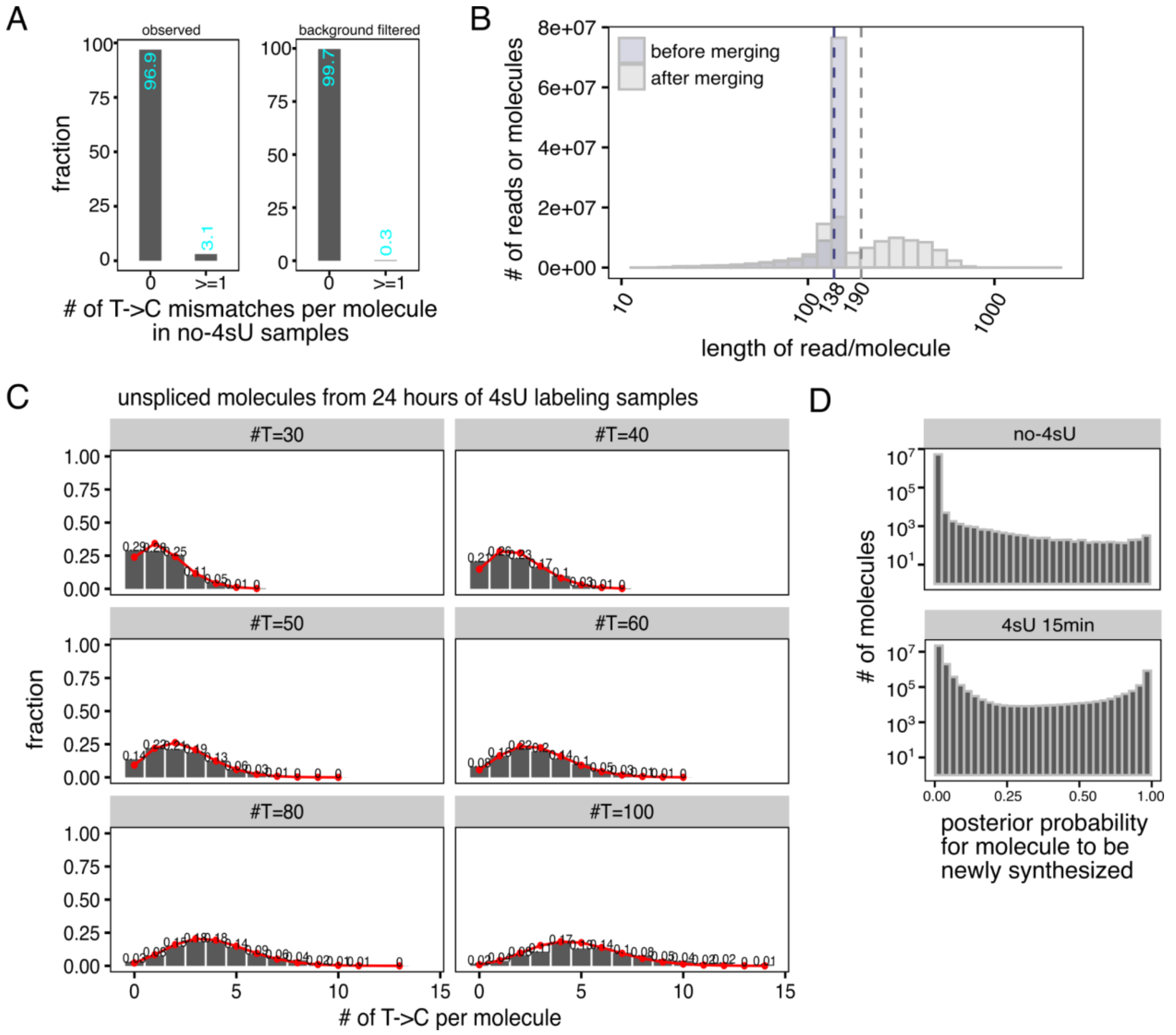

Appendix Figure S3. Identify newly synthesized molecules by merging reads from the same UMI and applying a Bayesian model.

(A) Filtering for SNPs and sequencing errors lower experimental background noise. (B) Length distributions of the sequenced molecules after merging reads. Data are from the 15 minutes 4sU labeled samples. Mean lengths are indicated by dashed lines. (C) Distributions of the number of T->C conversions per molecule of all unspliced molecules in 24 hours 4sU labeled samples resemble the Poisson distribution. Bar plots show the number of T->C conversions per molecule in molecules with a certain number of Ts. The red lines indicate the density plot of Poisson distribution with parameter λ (λ = 4sU incorporation rate multiplying the number of Ts). The mean T->C conversion rate calculated from 24 hours 4sU labeled samples was taken as the proxy of the 4sU incorporation rate (see Methods). (D) The distribution of posterior probability for merged molecules to be newly synthesized in no-4sU samples and 15 minutes 4sU labeled samples.

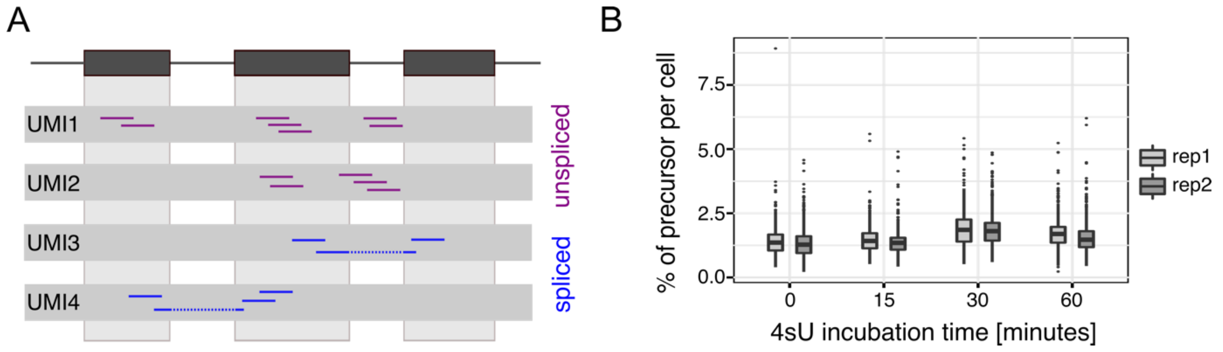

Appendix Figure S4. Splice status for each transcript is reliably identified by merging reads from the same UMI.

(A) A schematic illustration of the logic to assign splice status to the sequenced reads considering the UMI information. The drawing on top illustrates the gene annotation, with grey bars representing exons, linked by introns. The sketches under it show the reads identified by sequencing, assigned to different UMIs. The molecule is defined as unspliced if there is at least one read from it aligned to the intronic region. The molecule is defined as spliced if all reads from it map to the exonic regions. (B) The fractions of precursor (*i.e.*, unspliced) molecules of all SLAM-Drop-seq samples are constantly low (< 2%). The fraction of precursors per cell was calculated by dividing the number of precursor molecules (labeled) to the total number of molecules observed in each cell.

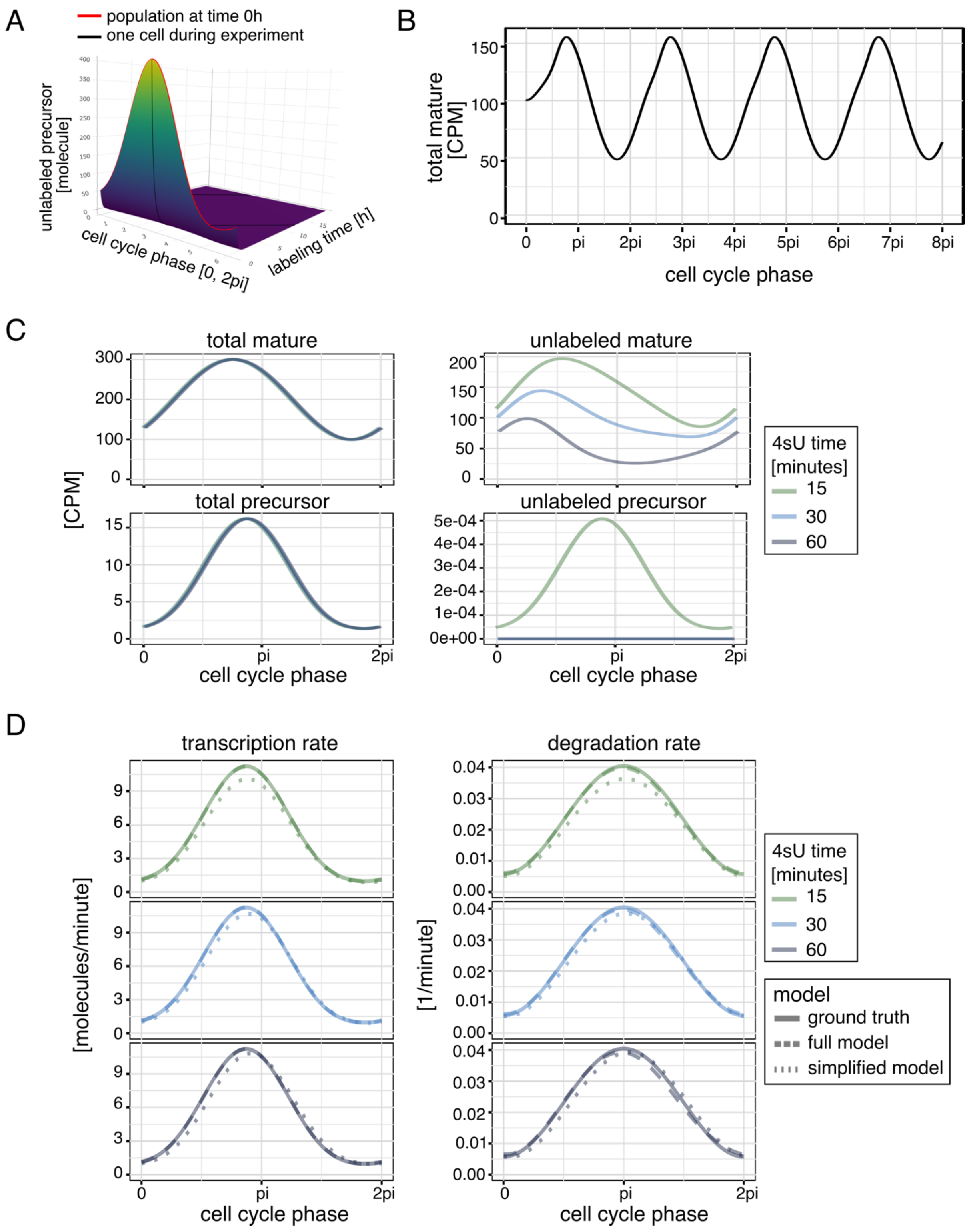

Appendix Figure S5. The calculated kinetic rates recapitulate the ground truths in synthetic data

(A) Schematic representation of the expression changes of unlabeled precursor molecules over the experimental time. (B) Illustration of the simulated gene expression profiles of one cycling gene over

the time duration of multiple cycles (cell cycle time: [0, 2pi]). The expression levels of the simulated cycling gene converge at the beginning and the end of the cell cycle. (C) Expression profiles of four different RNA types of synthetic data with given kinetic rates and total amount of RNAs (see Methods). Colors correspond to 4sU labeling times. Data for different 4sU labeling times were simulated for the same kinetic rates and total RNAs. (D) The calculated transcription and degradation rates for the simulated genes shown in (C) recapitulate the ground truths for both the full model and the simplified model.

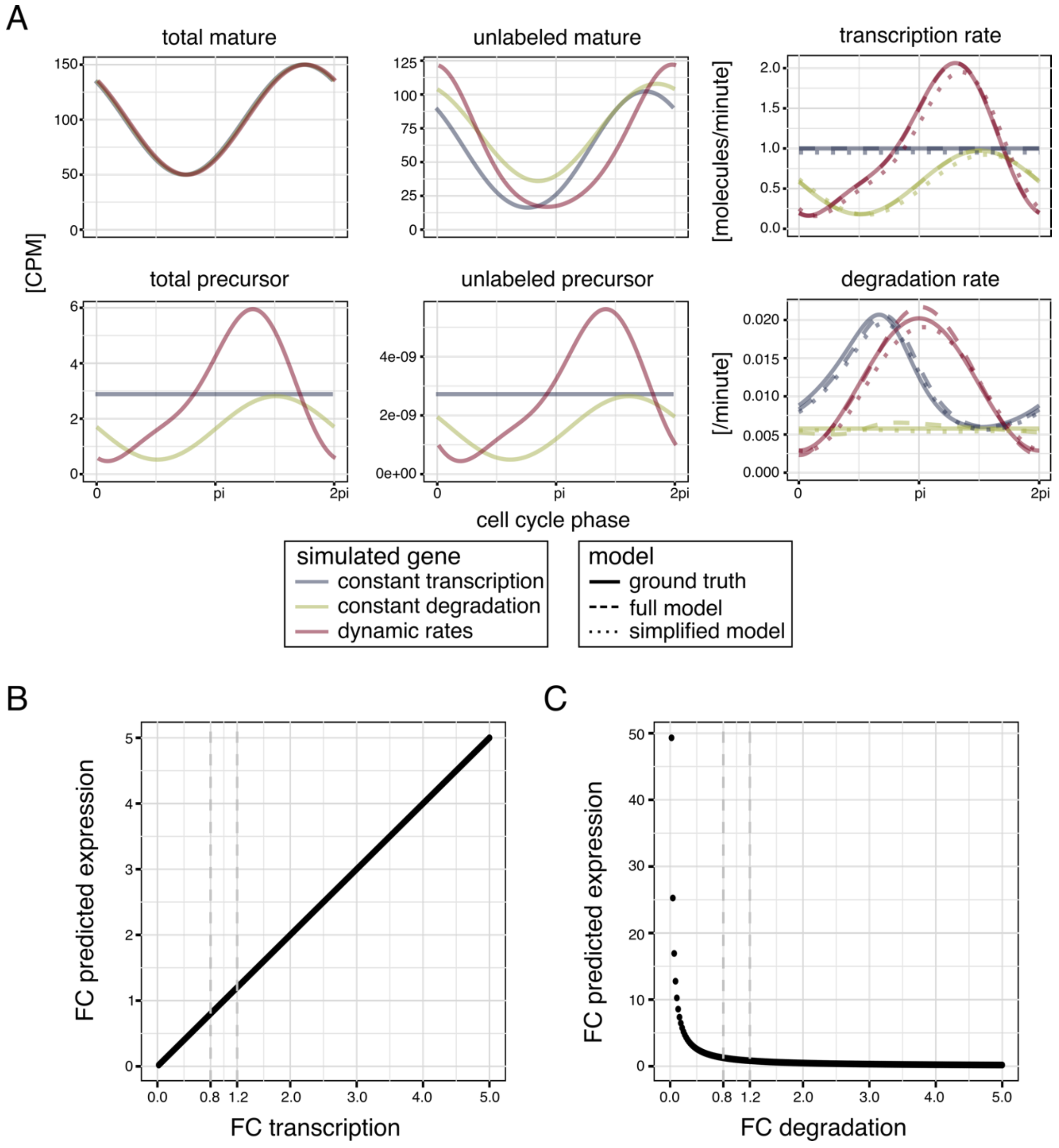

Appendix Figure S6. The analytical solutions to the kinetic model are capable of identifying different modes of kinetic rates.

(A) The calculated kinetic rates recapitulate the ground truths in simulated data when the simulated genes are synthesized under different kinetic regulation modes (*i.e.*, dynamic model, constant transcription and constant degradation). Leftmost and middle panels: gene expression profiles of representative simulated genes along the cell cycle. The ground truths of total mature RNA levels in the simulated genes are the same which are resulted from different kinetic rates. Rightmost panel: The calculated transcription and degradation rates recapitulate the ground truths in both full and the simplified models. (B) Prediction changes linearly upon transcription change in simulation data. The

plot shows the changes of the predicted gene expression (*i.e.*, prediction) upon transcription change in simulated data. Prediction changes linearly upon transcription change in simulation data. (C) The distribution of prediction changes against degradation rate changes in simulation data. Prediction change is almost linearly correlated to degradation change when the fold change of degradation is small (*e.g.*, 0.8-1.2).

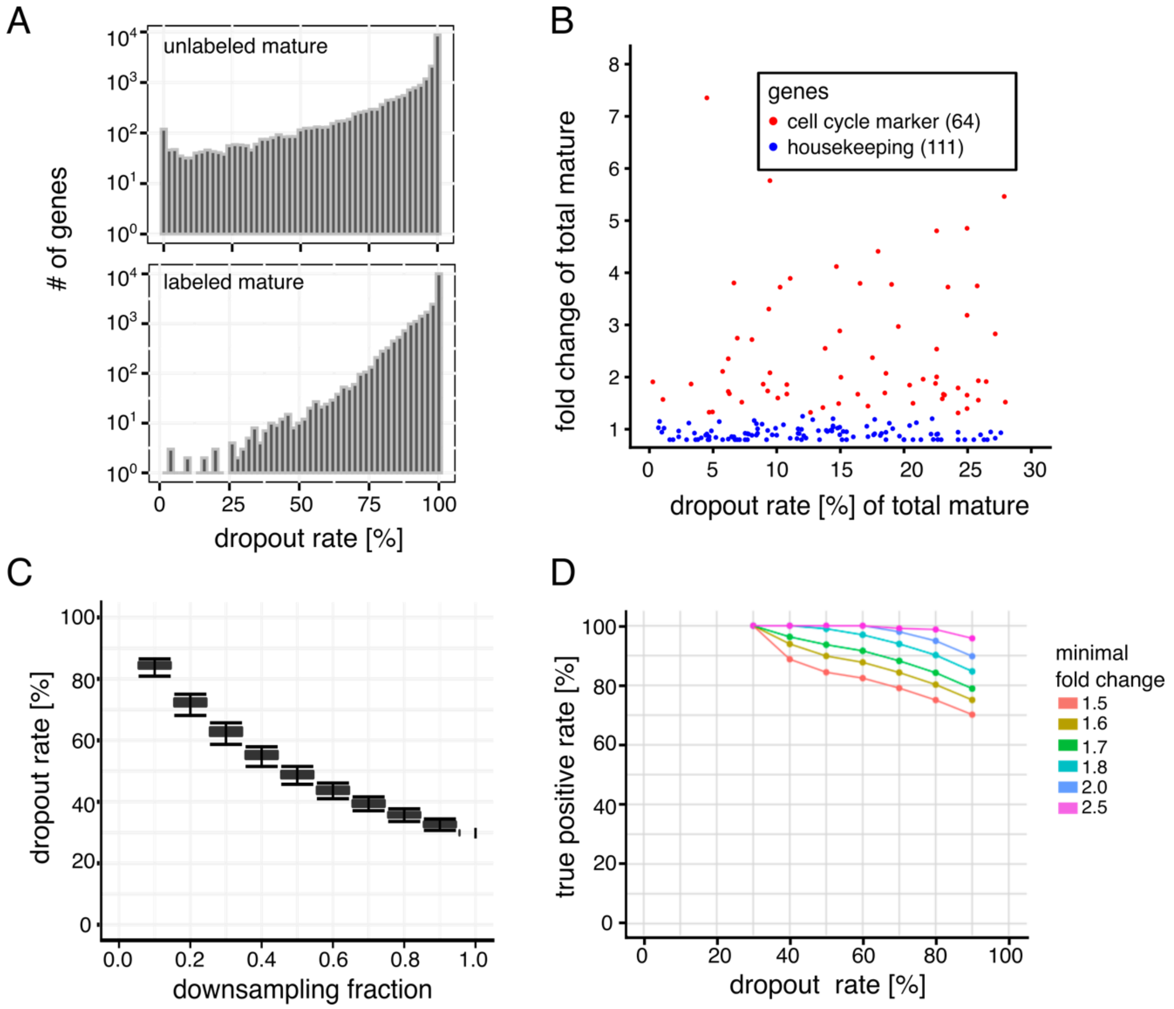

Appendix Figure S7. Downsampling analysis for identification of thresholds for dropout rates.

(A) Distribution of dropout rates of labeled and unlabeled RNAs. Data are from the 60 minutes 4sU labeled cells. Number of genes: 20,981. (B) Distributions of fold changes of gene expression (i.e., ratio between maximum and minimum values of gene expression profile along the cell cycle) against dropout rates of the total mature RNAs (15 minutes 4sU labeled samples). Colors indicate the selected well-known cell cycle marker genes and housekeeping genes. (C) Higher dropout rates are obtained by downsampling for genes shown in (B). (D) Distributions of true positive rates against dropout rates. Genes were defined as true positives if the well-known cycling genes (i.e., cell cycle marker genes) kept the classification as cycling genes after down-sampling. False negatives were defined for cases when house-keeping genes were identified as house-keeping genes after downsampling (see Methods). Genes were binned into different groups based on the minimal fold changes in expression as shown in the legend. True positive rate is the ratio of true positives to the sum of true positives and false negatives.

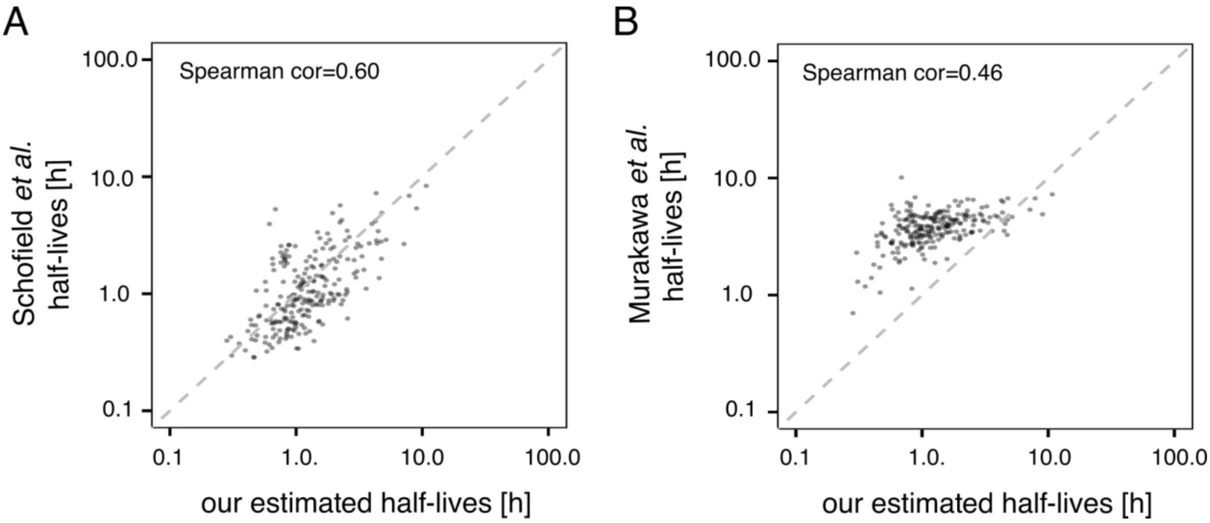

Appendix Figure S8. RNA half-life estimates correlate positively with published data.

(A-B) Mean half-lives calculated in our study are positively correlated to half-lives reported in published data (Murakawa *et al.*, 2015; Schofield *et al.*, 2018). Only the common genes between the well-predicted genes in our data and the published data are shown (n = 230). The mean half-life in our data was calculated from mean degradation rates over all cells for each gene (see Methods).

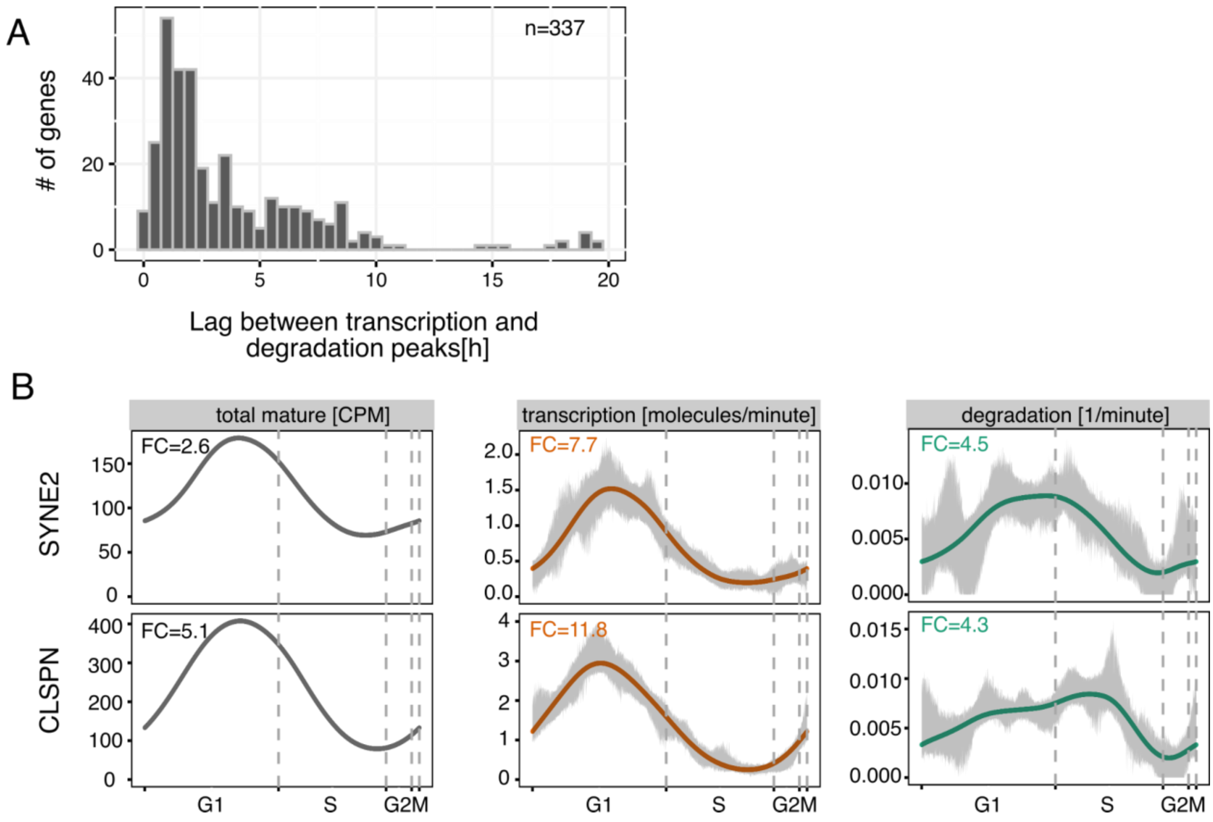

Appendix Figure S9. Kinetic rates of cycling genes are gene specific.

(A) Distributions of the time durations (lags) between the peaks (*i.e.*, the maximums) of transcription and degradation rates. Shown are the 337 cycling genes that are identified as regulated by both dynamic transcription and dynamic degradation rates. (B) Example profiles of genes that show similar expression but different kinetic rates. Fold change (FC) is calculated by dividing the maximum value to the minimum value of the profile along the cell cycle.
