# Supplementary material for: SLAM‐Drop‐seq reveals mRNA kinetic rates throughout the cell cycle
Source: Mol Syst Biol. 2023 Aug 28;19(10):e11427. doi: 10.15252/msb.202211427 (PMC10568207; doi:10.15252/msb.202211427)
Supplement: Supplementary file 2 — Expanded View Figures PDF [file MSB-19-e11427-s001.pdf]

Expanded View Figures

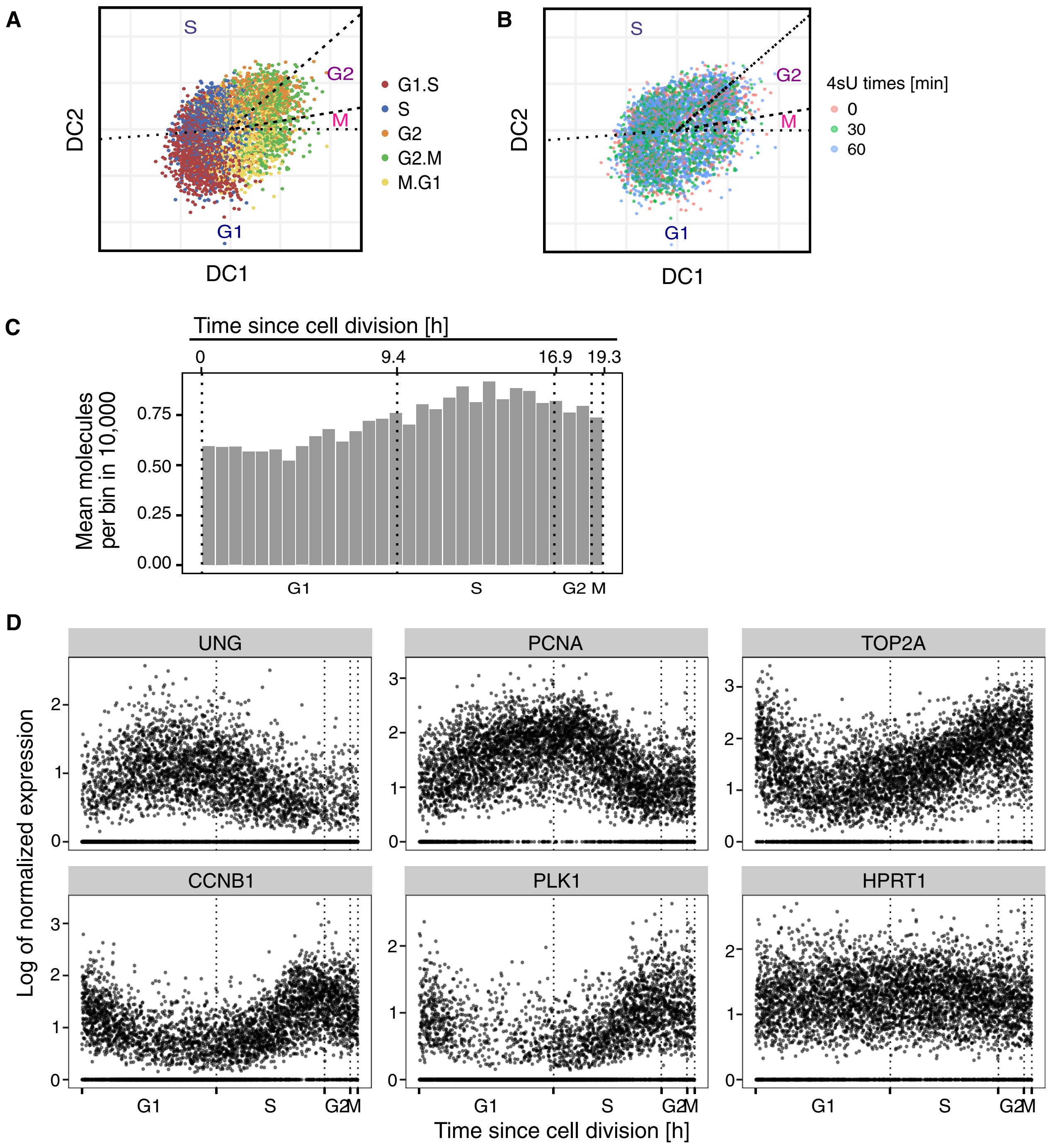

Figure EV1.

 © 2023 The Authors

Figure EV1. Asynchronous cells are sorted to a continuous cell cycle time using gene expression of single cells.

A The two-dimensional representation of the cells after dimensional reduction using Revelio (also see Fig 2A). Cells are pooled from batch 1 (i.e., 0, 30 and 60 min 4sU samples). Colors correspond to the cell cycle phase identities assigned using known cell cycle marker genes.
B Cells from different 4sU labeling times are randomly distributed within the cell clouds on the DC plot.
C The number of mean molecules per bin gradually increases along the cell cycle progression. Neighboring cells were grouped into 30 bins and the mean number of UMIs was used for the bar plots. The cells are the same ones as shown in (A) and (B).
D Representative gene expression profiles along the cell cycle (G1/S markers: UNG, PCNA; G2/M markers: CDK1, TOP2A, PKL1; House-keeping gene: HPRT1). The cells are the same ones as shown in (D). Gene expression levels were normalized by dividing the raw counts by the total RNA contents per cell and then scaled with 2×10^4.

Figure EV2. Time-dependent RNA expression, synthesis and degradation rates comparison between SLAM-Drop-seq and scEU-seq or sci-fate.

A Heatmaps of normalized z-score for observed total mature RNA expression (obs. exp.) for 98 cell cycle variable genes shared in both scEU-seq and SLAM-Drop-seq datasets. The scEU-seq gene expression values of RPE1-FUCCI cells are taken from sci-fate paper Supplementary Table S1 (Battich *et al*, 2020). Adjacent SLAM-Drop-seq cells were grouped to match the 301 bins of the scEU-seq data. Genes on the rows are ordered based on the positions of the maximum values of mean expression levels of the two datasets along the cell cycle. Binned cells (301) on the columns are ordered based on the assigned cell cycle pseudotimes.
B Heatmap representations of the transcription rates of genes shown in (A). scEU-seq transcription rates are taken from Supplementary Table 1 (Battich *et al*, 2020). The SLAM-Drop-seq transcription rates were normalized to the mean and log transformed as the scEU-seq data. Genes and cells are ordered in the same way as in (A).
C The normalized degradation rates are shown for the same genes and cells as shown in (A) and (B). scEU-seq degradation rates are taken from Supplementary Table S1 (Battich *et al*, 2020). The SLAM-Drop-seq degradation rates were normalized to the mean and log transformed.
D Heatmaps of normalized z-score for observed total mature RNA expression profiles for 140 overlapping cell cycle variable genes shared in both sci-fate and SLAM-Drop-seq datasets. Raw sci-fate sequencing data of A549 cells (Cao *et al*, 2020) were processed by following the sci-fate method (see details in Materials and Methods). Genes on the rows are ordered based on the maximum values of the mean across both data sets. Expression values for adjacent cells along the cell cycle are averaged to have the same number of columns between the two datasets.
E Heatmap representations of normalized transcription rates (normalized to the mean and then log transformed) of genes shown in (D). Genes and cells are in the same orders as in D.
F Heatmap representations of normalized degradation rates (normalized to the mean and then log transformed) of genes shown in (D). Genes and cells are in the same orders as in (D).

© 2023 The Authors

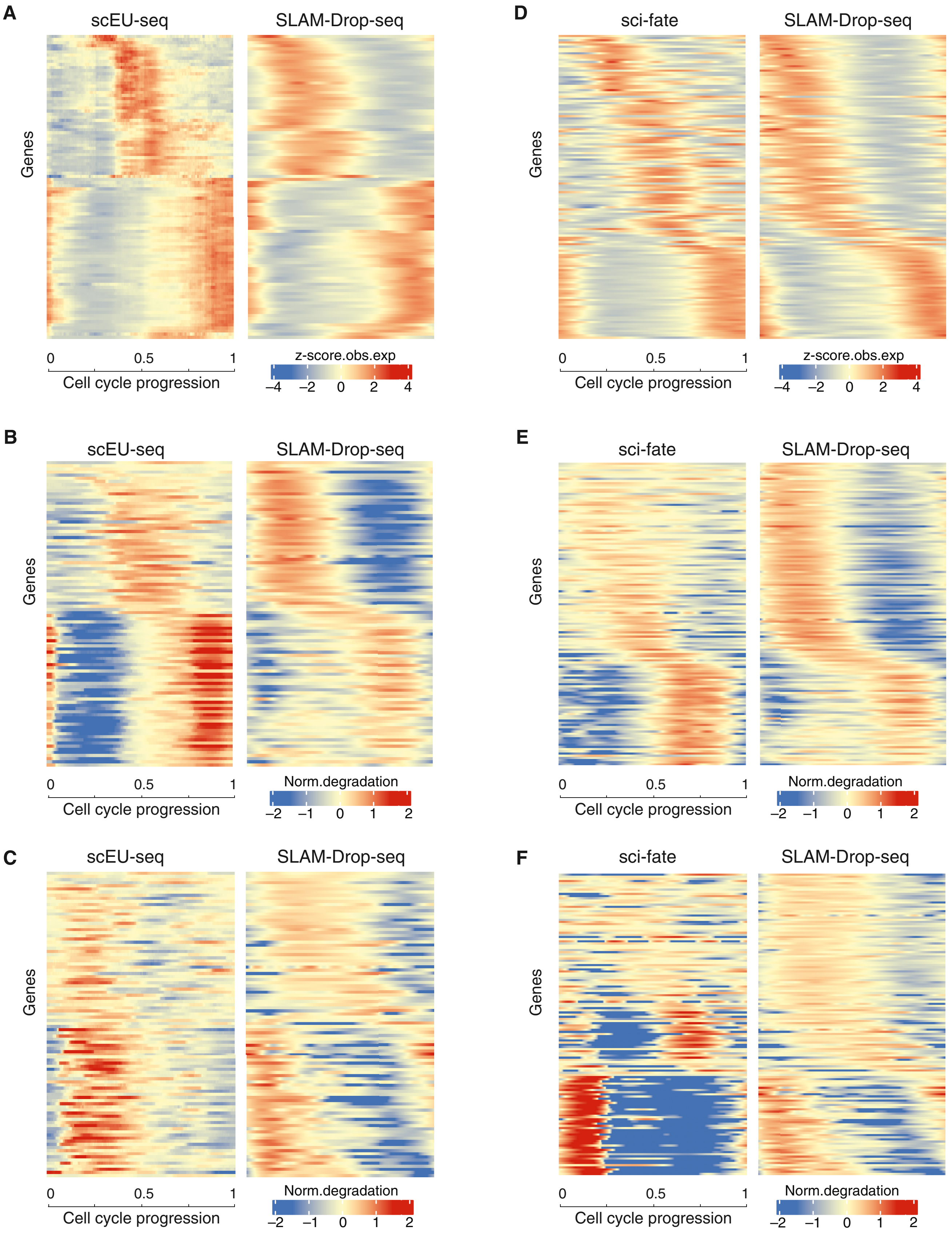

Figure EV2.

© 2023 The Authors
