# Supplementary material for: SLAM‐Drop‐seq reveals mRNA kinetic rates throughout the cell cycle
Source: Mol Syst Biol. 2023 Aug 28;19(10):e11427. doi: 10.15252/msb.202211427 (PMC10568207; doi:10.15252/msb.202211427)
Supplement: Supplementary file 5 [file MSB-19-e11427-s002.pdf]

molecular systems biology

SLAM-Drop-seq reveals mRNA kinetic rates throughout the cell cycle

Haiyue Liu, Roberto Arsiè, Daniel Schwabe, Marcel Schilling, Igor Minia, Jonathan Alles, Anastasiya Boltengagen, Christine Kocks, Martin Falcke, Nir Friedman, Markus Landthaler, and Nikolaus Rajewsky
DOI: 10.15252/msb.202211427

Corresponding author(s): Markus Landthaler (markus.landthaler@mdc-berlin.de) , Nikolaus Rajewsky (rajewsky@mdc-berlin.de)

Review Timeline:

Submission Date:	31st Oct 22
Editorial Decision:	13th Dec 22
Revision Received:	17th May 23
Editorial Decision:	5th Jul 23
Revision Received:	24th Jul 23
Accepted:	4th Aug 23

Editor: Jingyi Hou/Maria Polychronidou

Transaction Report:

(Note: With the exception of the correction of typographical or spelling errors that could be a source of ambiguity, letters and reports are not edited. Depending on transfer agreements, referee reports obtained elsewhere may or may not be included in this compilation. Referee reports are anonymous unless the Referee chooses to sign their reports.)

13th Dec 2022

Manuscript Number: MSB-2022-11427
Title: SLAM-Drop-seq reveals mRNA kinetic rates throughout the cell cycle
Author: Markus Landthaler
Haiyue Liu
Roberto Arsiè
Daniel Schwabe
Marcel Schilling
Igor Minia
Jonathan Alles
Anastasiya Boltengagen
Christine Kocks
Martin Falcke
Nir Friedman
Nikolaus Rajewsky

Dear Markus,

Thank you for submitting your work to Molecular Systems Biology. I would like to apologize for the slow process, which was due to the late arrival of reviewers' reports. We have now heard back from two of the three reviewers who agreed to evaluate your manuscript. Unfortunately, after a series of reminders, we did not manage to obtain a report from Reviewer #2. In the interest of time, and since the recommendations of the other two reviewers are quite similar, I prefer to make a decision now rather than further delay the process. If we receive the comments from Reviewer #2, we will send them to you, and you can address the issues raised by Reviewer #2 together with those raised by the other two reviewers. You will see from the comments below that Reviewers #1 and #3 find the manuscript potentially interesting. They raise, however, several important points, which we would ask you to address in a revision of this work.

I think the reviewers' recommendations are relatively straightforward, so there is no need to reiterate their comments. Importantly, Reviewer #3 pointed out that the overall biological insight provided remained relatively modest and mentioned that it would be important to compare the current results to those in the Battich paper in more detail and to demonstrate the generality of the presented findings (in HEK293 cells) for human cell cycle biology. All other issues raised by the reviewers need to be satisfactorily addressed as well. As you may already know, our editorial policy allows in principle a single round of major revision, and it is therefore essential to provide responses to the reviewers' comments that are as complete as possible. Please feel free to contact me in case you would like to discuss in further detail any of the issues raised by the reviewers.

On a more editorial level, we would ask you to address the following issues:

- Please provide a .docx formatted version of the manuscript text (including legends for main figures, EV figures and tables). Please make sure that the changes are highlighted to be clearly visible.

- Please provide individual production quality figure files as .eps, .tif, .jpg (one file per figure).

-Please provide a .docx formatted letter INCLUDING the reviewers' reports and your detailed point-by-point responses to their comments. As part of the EMBO Press transparent editorial process, the point-by-point response is part of the Review Process File (RPF), which will be published alongside your paper.

-Please note that all corresponding authors are required to supply an ORCID ID for their name upon submission of a revised manuscript.

-We replaced Supplementary Information with Expanded View (EV) Figures and Tables that are collapsible/expandable online (see examples in http://msb.embopress.org/content/11/6/812). A maximum of 5 EV Figures can be typeset. EV Figures should be cited as 'Figure EV1, Figure EV2" etc... in the text and their respective legends should be included in the main text after the legends of regular figures.

Additional Tables/Datasets should be labeled and referred to as Table EV1, Dataset EV1, etc. Legends have to be provided in a separate tab in case of .xls files. Alternatively, the legend can be supplied as a separate text file (README) and zipped together with the Table/Dataset file.

For the figures and tables that you do NOT wish to display as Expanded View figures, they should be bundled together with their legends in a single PDF file called *Appendix*, which should start with a short Table of Content. Each legend should be below

the corresponding Figure/Table in the Appendix. Appendix figures and tables should be referred to in the main text as: "Appendix Figure S1, Appendix Figure S2, Appendix Table S1" etc. See detailed instructions regarding expanded view here: https://www.embopress.org/page/journal/17444292/authorguide#expandedview.

-Before submitting your revision, primary datasets (and computer code, where appropriate) produced in this study need to be deposited in an appropriate public database (seehttp://msb.embopress.org/authorguide - dataavailability https://www.embopress.org/page/journal/17444292/authorguide#dataavailability).

Please remember to provide a reviewer password if the datasets are not yet public.

The accession numbers and database should be listed in a formal "Data Availability" section (placed after Materials & Method) that follows the model below (see also https://www.embopress.org/page/journal/17444292/authorguide#dataavailability). Please note that the Data Availability Section is restricted to new primary data that are part of this study.

Data availability

The datasets (and computer code) produced in this study are available in the following databases:

- RNA-Seq data: Gene Expression Omnibus GSE46843 (https://www.ncbi.nlm.nih.gov/geo/query/acc.cgi?acc=GSE46843)
- [data type]: [name of the resource] [accession number/identifier/doi] ([URL or identifiers.org/DATABASE:ACCESSION])

*** Note - All links should resolve to a page where the data can be accessed. ***

-At EMBO Press we ask authors to provide source data for the main figures. Our source data coordinator will contact you to discuss which figure panels we would need source data for and will also provide you with helpful tips on how to upload and organize the files.

- Our journal encourages inclusion of *data citations in the reference list* to directly cite datasets that were re-used and obtained from public databases. Data citations in the article text are distinct from normal bibliographical citations and should directly link to the database records from which the data can be accessed. In the main text, data citations are formatted as follows: "Data ref: Smith et al, 2001". In the Reference list, data citations must be labeled with "[DATASET]". A data reference must provide the database name, accession number/identifiers and a resolvable link to the landing page from which the data can be accessed at the end of the reference. Further instructions are available at .

- We updated our journal's competing interests policy in January 2022 and request authors to consider both actual and perceived competing interests. Please review the policy https://www.embopress.org/competing-interests and update your competing interests if necessary.

Please use the heading "Disclosure statement and competing interests".

- All Materials and Methods need to be described in the main text. Since the study presents a new approach, we would kindly ask you to use 'Structured Methods', our new Materials and Methods format. According to this format, the Material and Methods section should include a Reagents and Tools Table (listing key reagents, experimental models, software and relevant equipment and including their sources and relevant identifiers) followed by a Methods and Protocols section in which we encourage the authors to describe their methods using a step-by-step protocol format with bullet points, to facilitate the adoption of the methodologies across labs. More information on how to adhere to this format as well as downloadable templates (.doc or .xls) for the Reagents and Tools Table can be found in our author guidelines: < https://www.embopress.org/page/journal/17444292/authorguide#researcharticleguide>. An example of a Method paper with Structured Methods can be found here: .

-Regarding data quantification:
Please ensure to specify the name of the statistical test used to generate error bars and P values, the number (n) of independent experiments (please specify technical or biological replicates) underlying each data point and the test used to calculate p-values in each figure legend. Discussion of statistical methodology can be reported in the materials and methods section, but figure legends should contain a basic description of n, P and the test applied.
Graphs must include a description of the bars and the error bars (s.d., s.e.m.).
Please also include scale bars in all microscopy images.

- Please provide a "standfirst text" summarizing the study in one or two sentences (approximately 250 characters, including space), three to four "bullet points" highlighting the main findings and a "synopsis image" (550px width and 400-600 px height, PNG format) to highlight the paper on our homepage.
Here are a couple of examples:
https://www.embopress.org/doi/10.15252/msb.20199356
https://www.embopress.org/doi/10.15252/msb.20209475

https://www.embopress.org/doi/10.15252/msb.209495

When you resubmit your manuscript, please download our CHECKLIST (https://www.embopress.org/pb-assets/embo-site/EMBO%20Press%20Author%20Checklist-1642513524327.xlsx) and include the completed form in your submission. *Please note* that the Author Checklist will be published alongside the paper as part of the transparent process (https://www.embopress.org/page/journal/17444292/authorguide#transparentprocess).

If you feel you can satisfactorily deal with these points and those listed by the referees, you may wish to submit a revised version of your manuscript. Please attach a covering letter giving details of the way in which you have handled each of the points raised by the referees. A revised manuscript will be once again subject to review and you probably understand that we can give you no guarantee at this stage that the eventual outcome will be favorable.

I look forward to receiving the revised manuscript soon.

Sincerely,
Jingyi

Jingyi Hou, PhD
Scientific Editor
Molecular Systems Biology

We realize that it is difficult to revise to a specific deadline. In the interest of protecting the conceptual advance provided by the work, we recommend a revision within 3 months (13th Mar 2023). Please discuss the revision progress ahead of this time with the editor if you require more time to complete the revisions. Use the link below to submit your revision:

https://msb.msubmit.net/cgi-bin/main.plex

IMPORTANT: When you send your revision, we will require the following items:
1. the manuscript text in LaTeX, RTF or MS Word format
2. a letter with a detailed description of the changes made in response to the referees. Please specify clearly the exact places in the text (pages and paragraphs) where each change has been made in response to each specific comment given
3. three to four 'bullet points' highlighting the main findings of your study
4. a short 'blurb' text summarizing in two sentences the study (max. 250 characters)
5. a 'thumbnail image' (550px width and max 400px height, Illustrator, PowerPoint or jpeg format), which can be used as 'visual title' for the synopsis section of your paper.
6. Please include an author contributions statement after the Acknowledgements section (see https://www.embopress.org/page/journal/17444292/authorguide)
7. Please complete the CHECKLIST available at (https://bit.ly/EMBOPressAuthorChecklist).
Please note that the Author Checklist will be published alongside the paper as part of the transparent process (https://www.embopress.org/page/journal/17444292/authorguide#transparentprocess).
8. When assembling figures, please refer to our figure preparation guideline in order to ensure proper formatting and readability in print as well as on screen:
https://bit.ly/EMBOPressFigurePreparationGuideline
See also figure legend guidelines: https://www.embopress.org/page/journal/17444292/authorguide#figureformat
9. Please note that corresponding authors are required to supply an ORCID ID for their name upon submission of a revised manuscript (EMBO Press signed a joint statement to encourage ORCID adoption).
(https://www.embopress.org/page/journal/17444292/authorguide#editorialprocess)
Currently, our records indicate that the ORCID for your account is 0000-0002-1075-8734.

Please click the link below to modify this ORCID:
Link Not Available

10. At EMBO Press we ask authors to provide source data for the main manuscript figures. Our source data coordinator will contact you to discuss which figure panels we would need source data for and will also provide you with helpful tips on how to upload and organize the files.

The system will prompt you to fill in your funding and payment information. This will allow Wiley to send you a quote for the article processing charge (APC) in case of acceptance. This quote takes into account any reduction or fee waivers that you may be eligible for. Authors do not need to pay any fees before their manuscript is accepted and transferred to the publisher.

EMBO Press participates in many Publish and Read agreements that allow authors to publish Open Access with reduced/no publication charges. Check your eligibility: https://authorservices.wiley.com/author-resources/Journal-Authors/open-access/affiliation-policies-payments/index.html

As a matter of course, please make sure that you have correctly followed the instructions for authors as given on the submission website.

*** PLEASE NOTE *** As part of the EMBO Press transparent editorial process initiative (see our Editorial at https://dx.doi.org/10.1038/msb.2010.72), Molecular Systems Biology publishes online a Review Process File with each accepted manuscripts. This file will be published in conjunction with your paper and will include the anonymous referee reports, your point-by-point response and all pertinent correspondence relating to the manuscript. If you do NOT want this File to be published, please inform the editorial office at msb@embo.org within 14 days upon receipt of the present letter.

Reviewer #1:

Integrating temporal dimension in single-cell RNA-seq (scRNA-seq) data is a major challenge to uncovering fast and dynamic biological processes. Recently, a set of technologies integrating RNA metabolic labeling, nucleoside conversion, and scRNA-seq have emerged adding RNA kinetics dimension to scRNA-seq (reviewed by Erhard et al Nat Rev Met Primers 2022). Here, building on RNA metabolic labeling, Liu et al. are proposing a refined experimental scSLAM-seq workflow based on microfluidic droplets (SLAM-Drop-seq), and 4sU- based RNA metabolic labeling, and the authors developed a companion analytical tool coined Eskrate to calculate the synthesis as well as the degradation rates of captured genes.

To demonstrate the applicability of their workflow, the authors have investigated gene expression regulation over the cell cycle in HEK293 cells. After restraining the analysis to a set of genes known to be involved in cell cycle progression, transcription and degradation rates have been estimated over time for a total of 399 genes. Further analysis has revealed that gene expression, in terms of transcript abundance, is dynamically regulated by variations of both transcription and degradation rates for the vast majority of these genes. Only a small gene fraction appears to be regulated by modulation of only one of these two kinetic rates.

This publication comes timely as scSLAM-seq approaches are developing fast in many labs. The use of the cell cycle as a biological model to develop the method is very elegant and the authors achieve an impressive temporal resolution down to a few minutes. This is a first to my knowledge and the dataset generated will be used by many labs. Yet, I have major comments.

Experimentally, 'SLAM-Drop-seq' builds on an in-house drop-seq that delivers an average of 2800 genes per HEK cell. HEK cells are a cell line that contains many RNA molecules and 2800 genes sound relatively low. Is it due to the low performance of the Dolomite system compared to the performance of a 10x machine? Another hypothesis: does the fixation with methanol leads to cellular leakage? It is also common to have higher ambient RNA levels from methanol-fixed cells likely because of transcript leakage. In that case, loss of RNA would lead to over-estimation of degradation rates. The authors should demonstrate if significant RNA leakage has happened or not in their dataset.

The authors have concatenated reads originating from the "same UMI" to call 'old' and 'new' RNA molecules. I am surprised to see that the "merged molecules" marginally increase in the size of the original reads from 138 bp to 190 bp. To quantify the old and new RNA, I am not sure to understand how it is different from the GRAND SLAM methods (Erhard et al. PMID: 29949974).

The authors should rule out the toxicity of 4sU (Fig 1B). With such a short 4sU labeling time, detectable transcriptional toxicity is more likely to affect transcripts with short half-lives. Could the authors show if transcripts with short estimated half-lives are downregulated in 4sU-treated cells (for instance with a scatterplot)?

The authors distinguish 'synthesized' (new RNA), 'pre-existing' (old RNA), 'precursor' (unspliced), and 'mature' (spliced). Can the authors add the 5'>3' coverage of the reads? If there is a bias, what are the consequences for the 'precursor' quantification? I propose this be added to the discussion.

Supp. Fig 5C is very important I feel as it gives the 'real-time'. Is it possible to bring this panel in the main figures? How exactly the real-time has been calculated here?

Often we use RNA metabolic labeling with 2 or 3 hours of labeling time. How does labeling time influence Eskrate? Determining precisely the kinetic rates from RNA metabolic labeling experiments is dependent on the time of labeling as the optimal duration for any RNA species is a link to its turnover (PMID: 31390362; PMID: 35459101). Is that the reason why the authors found their fittings to be more accurate with 60min labeling (Fig S6D)?

To demonstrate the applicability to other models, the authors could try to apply Eskrate on the sci-fate dataset (PMID: 32284584). They can restrict the analysis to the unstimulated cells of the dataset and investigate the RNA kinetics over the cell

cycle in A549 cells. This will show if the biological conclusions are transposable to another cell line and if the workflow applies to longer labeling times (2 hours). In addition, the sci-fate dataset has a higher percentage of intronic reads. This might be a good opportunity for the authors to run Eskrate with the full model, including processing rates.

The authors have tried different 4sU labeling times (15, 30, and 60min) but have restrained their analysis for kinetic rate calculation to the cells treated for 60min. The reason is clearly explained by the authors. Nevertheless, the authors report on 5 to 10% new RNA after 15 and 30min labeling (Fig 1E). To my knowledge, this is the first time that I see such short times for a SLAM-seq-based approach. Do the authors think that their estimation of newly synthesized RNA is still accurate with 15min labeling (beyond the accuracy of kinetic rate estimation)? This is important information for the community to design future experiments

Reviewer #3:

This paper introduces a useful methodology SLAM-Drop, a method that allows the analysis of nascent and mature transcripts from single cells. The advantage of SLAM-seq over other methods such as scEU-seq is that no extra purifications are needed. The paper also introduces an R package Eskrate to optimally analyze SLAM-Drop data. The protocols and methods are clearly described.

As a test they then analyze mRNA expression levels during the mammalian cell cycle, showing that mRNA degradation is an important contribution of the cyclic expression profiles. The conclusions on the cell cycle RNA profiles are similar to those reached by Battich Science 2020 using scEU-seq, however, the Battich analysis went deeper. Overall the biological analysis is fairly succinct as it seems the emphasis is on the methodology, both experimental and computational. Given the extensive data that were generated, and elaborate modeling that was developed, it appears that these were not exploited to their full potential (Figures 3 and 4).

Major points:

1. Some results seem qualitatively different from those of Battich (RPE cells). For example here, there is a majority of genes with transcription starting in early G1 (Fig 3B), which in Battich there are no such genes, there is always a significant gap time after mitosis without transcription.

2. More generally, since the Battich paper is a reference, it seems essential to compare the findings in more detail (gene by gene), and investigate whether the differences are of technical origin (sequencing or analysis), or have a biological significance.

3. There is a claim in the Discussion that the rates inferred here are better resolved temporally (less than one minute) that other approaches, but this is not shown in the paper and also not exploited to illustrate some interesting biology.

4. "Fig S5C: The number of mean molecules per bin gradually increases along the cell cycle progression and roughly halves after the cell division." It appears the data go from about 0.6 to 0.8, which is significantly less than a factor of 2. This could be due to either inaccurate cell cycle phase assignment or an issue with the experimental approach.

5. From a cell cycle perspective, the insights are fairly limited. The finding that mRNA degradation plasma significant was shown in Battich 2020. It is valuable to confirm this, but it hold be augmented with more explicit comparisons.

6. Cell-cycle biology: one histone transcript is being discussed, but this is confirmatory rather than novel. Also, since histone genes are not polyadenylated, this may be a very atypical gene. Can it be measured reliably?

7. How typical are genetically unstable HEK293 cells for human cell cycle biology? It would be useful to have a comparative angle to know how general the findings are.

Minor:
Labeling times of 0 15, 30 and 60 minutes are described. Are all used in the subsequent analyses and models?

Authors' Response to Reviewers

We thank the reviewers for their constructive feedback and thoughtful remarks. Addressing the different comments helped us improve the quality and clarity of the submitted manuscript. We are encouraged that the reviewers found the approaches we developed novel and relevant for the scientific community. Following their comments and suggestions, we have conducted a series of comparisons and analyses to address their concerns.

As suggested, we added comparisons of HEK293 cell cycle time-dependent kinetic rates to those calculated from two other human cell lines (A549 and RPE1-FUCCI cells). To this end, the published sci-fate (Cao *et al.,* 2020) and scEU-seq (Battich *et al.,* 2020) datasets were used. We compared the observed mRNA levels and their transcription and degradation rates along the cell cycle for common cell cycle variable genes (Fig. EV2 A-C). The comparison of kinetic rates of cell cycle regulated genes showed an overall similar time-dependent pattern across the different human cell lines analyzed. These results also showed that the computational method we developed (Eskrate) is capable of calculating time-resolved mRNA kinetic rates from different metabolically labeled scRNA-seq datasets.

We corrected some unclear statements regarding the time resolution and the doubling of RNA molecules during the cell cycle. Furthermore, we clarified the 3'-end sequencing bias of our approach and discussed its implication.

Below, we provide a point-by-point response (in blue) to the reviewers' comments and suggestions. We hope the reviewers find that the major comments are fully addressed and can recommend our manuscript for publication.

Reviewer #1

Integrating temporal dimension in single-cell RNA-seq (scRNA-seq) data is a major challenge to uncovering fast and dynamic biological processes. Recently, a set of technologies integrating RNA metabolic labeling, nucleoside conversion, and scRNA-seq have emerged adding RNA kinetics dimension to scRNA-seq (reviewed by Erhard et al Nat Rev Met Primers 2022). Here, building on RNA metabolic labeling, Liu et al. are proposing a refined experimental scSLAM-seq workflow based on microfluidic droplets (SLAM-Drop-seq), and 4sU- based RNA metabolic labeling, and the authors developed a companion analytical tool coined Eskrate to calculate the synthesis as well as the degradation rates of captured genes.

To demonstrate the applicability of their workflow, the authors have investigated gene expression regulation over the cell cycle in HEK293 cells. After restraining the analysis toa set of genes known to be involved in cell cycle progression, transcription and degradation rates have been estimated over time for a total of 399 genes. Further analysis has revealed that gene expression, in terms of transcript abundance, is dynamically regulated by variations of both transcription and degradation rates for the vast majority of these genes. Only a small gene fraction appears to be regulated by modulation of only one of these two kinetic rates.

This publication comes timely as scSLAM-seq approaches are developing fast in many labs. The use of the cell cycle as a biological model to develop the method is very elegant and the authors achieve an impressive temporal resolution down to a few minutes. This is a first to my knowledge and the dataset generated will be used by many labs. Yet, I have major comments.

1. Experimentally, 'SLAM-Drop-seq' builds on an in-house drop-seq that delivers an average of 2800 genes per HEK cell. HEK cells are a cell line that contains many RNA molecules and 2800 genes sound relatively low. Is it due to the low performance of the Dolomite system compared to the performance of a 10x machine? Another hypothesis: does the fixation with methanol lead to cellular leakage? It is also common to have higher ambient RNA levels from methanol-fixed cells likely because of transcript leakage. In that case, loss of RNA would lead to over-estimation of degradation rates. The authors should demonstrate if significant RNA leakage has happened or not in their dataset.

We thank the reviewer for pointing out those possible reasons for low capture rates and the concern of influences on the kinetic rates. First of all, 2800 was the median number of detected genes per cell in the no-4sU sample, which was the lowest across all the SLAM-Drop-seq libraries. Since the no-4sU sample was used as control, we sequenced 1/3 as deep as the other samples in the same batch. Thus, it was expected to have a low number of genes. Second, the median number of genes per cell in other libraries were ranging from 2934 to 5071. This is not relatively low in terms of the Dolomite Nadia system since it is known it has lower capture efficiency with respect to the more performative (and more expensive) 10x Genomics instrument (Yamawaki *et al*, 2021).

The methanol fixation might induce some degree of RNA leakage, but the relative concentrations in our approach are the same used in the many published papers and regarded as the “standard” for single cell library preparation (Alles *et al*, 2017). A recent publication demonstrated how methanol fixation has only minor effects on single cell sequencing approaches such as Drop-seq (Wang *et al*, 2021). In our analysis, the cells used for rate calculation were selected to have a sufficient number of reads based on the so-called ‘knee plot’ (as shown below, data from one sample) described in (Macosko *et al*, 2015). The knee plots (see below plot) showed clear inflection points which indicates the cells that are amplified from a STAMP separate from cells amplified from empty beads that contain ambient RNAs. Furthermore, the mitochondrial RNA contents in the sequenced single cells are low (more than 98% cells have less than 5% mtRNAs), indicating the cells are not damaged and cytoplasmic RNAs are preserved. Thus, we think the leakage effect of methanol fixation is negligible for the analysis.

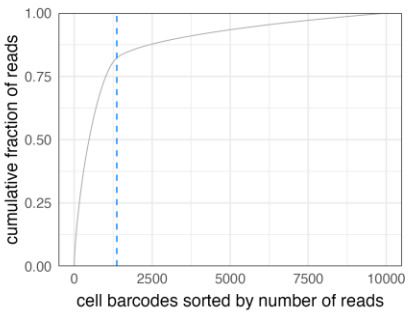

2. The authors have concatenated reads originating from the "same UMI" to call 'old' and 'new' RNA molecules. I am surprised to see that the "merged molecules" marginally increase in the size of the original reads from 138 bp to 190 bp. To quantify the old and new RNA, I am not sure to understand how it is different from the GRAND SLAM methods (Erhard et al. PMID: 29949974).

We were able to concatenate different reads from the same UMI due to the fact that two rounds of PCR amplifications were conducted during library preparation. The first round of PCR amplifies the cDNA libraries, followed by fragmentation and a second round of PCR amplification of the fragments. The sizes of the fragments were controlled to be around 600-1000 base pairs, resulting in limited diversity of the different reads from the same UMI. Nonetheless, the median increase of 38% in read length was sufficient in our case to accurately quantify the newly synthesized and pre-existing RNA molecules. Before merging, the distribution of T->C conversions per read was zero-inflated, which made it hard to distinguish new molecules from old ones. After merging, we observed the probabilities for a molecule to be new were either 0 or 1-inflated (Appendix Fig. S3D), making the identification of new molecules more reliable.

The GRAND-SLAM approach (Jürges *et al*, 2018) evaluates 4sU-labeled and unlabeled transcripts using a Bayesian framework to estimate uncertainties, but in the context of cell population (*i.e.* not at the single cell level, as in our approach). We applied a Bayesian framework to identify new molecules, based on the observation of T->C conversions. We differ from the GRAND-SLAM method in how we estimated the 4sU incorporation rate. In GRAND-SLAM, 4sU incorporation rates and the ratio of new to total transcripts is calculated using the EM-algorithm; whereas we approximated 4sU incorporation rate using a Poisson distribution, based on the data from a long 4sU labeling experiment (24 hours, Fig. S3C).

3. The authors should rule out the toxicity of 4sU (Fig 1B). With such a short 4sU labeling time, detectable transcriptional toxicity is more likely to affect transcripts with short half-lives. Could the authors show if transcripts with short estimated half-lives are downregulated in 4sU-treated cells (for instance with a scatterplot)?

We thank the reviewer for the thoughtful suggestions. The toxicity of 4sU on maturation of rRNA and splicing of pre-mRNA has been shown for labeling times much longer than the one used in our approach (>12 hours) (Burger *et al*, 2013; Altieri & Hertel, 2021). Previous experiments (Hafner *et al*, 2009) with higher concentration and longer time of 4sU incubation (1mM for 12 hours) with respect to the one used in our method, did not affect the transcriptional output of HEK293 cells. Nonetheless, we conducted quality control analysis and showed that the percentage of newly synthesized molecules increases proportionally with the timing of incubation with 4sU (Figure 1E), excluding any major effect of the nucleotide analog on the cellular (Hafner *et al*, 2009) transcriptional output.

4. The authors distinguish 'synthesized' (new RNA), 'pre-existing' (old RNA), 'precursor' (unspliced), and 'mature' (spliced). Can the authors add the 5'>3' coverage of the reads? If there is a bias, what are the consequences for the 'precursor' quantification? I propose this be added to the discussion.

We thank the reviewer's suggestion. The SLAM-Drop-seq approach is based (as many single cell methods) on capture of polyadenylated RNAs and sequencing of the region close-by, resulting in 3' bias of the sequenced reads. To more efficiently quantify the precursor and the 4sU incorporation, we computationally merged reads with the same UMI. This could potentially extend the coverage at the UMI level to the gene body. However, we are aware of the 3' bias of the reads coverage as shown below. We have added discussions on the potential effects of the coverage bias to the kinetic rates.

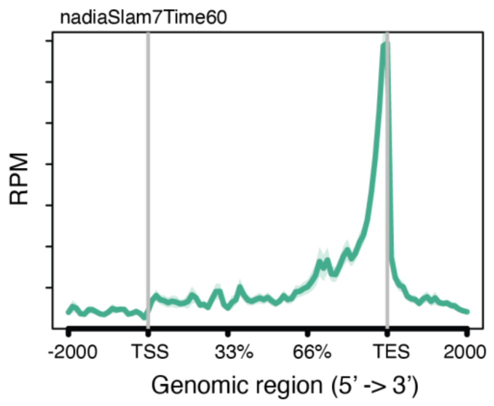

5. Supp. Fig 5C is very important I feel as it gives the 'real-time'. Is it possible to bring this panel in the main figures? How exactly the real-time has been calculated here?

We thank the reviewer for appreciating the importance of assigning the single cells with cell cycle times. The cell cycle time is important for the kinetic rate analysis. However, it is not novel. We applied the published method called Revelio (Schwabe *et al*, 2020) and assigned the cell cycle time following the main procedures described in the paper. After the 2D cell cycle representation was achieved using PCA-based method (Fig EV1 B), the cell division time was identified based on the assumption that molecule number roughly halves after the cell division. The real cell cycle time was assigned to each cell based on its order relative to the cell division time and the assumption that the cells were evenly distributed along the cell cycle process (See Material and Methods). This has been described in the Revelio method (Schwabe *et al*, 2020) as well. Nonetheless, we acknowledged the comment of the reviewer and shifted the figure to the Expanded View section (Figure EV2).

6. Often we use RNA metabolic labeling with 2 or 3 hours of labeling time. How does labeling time influence Eskrate? Determining precisely the kinetic rates from RNA metabolic labeling experiments is dependent on the time of labeling as the optimal duration for any RNA species is a link to its turnover (PMID: 31390362; PMID: 35459101). Is that the reason why the authors found their fittings to be more accurate with 60min labeling (Fig S6D)?

We thank the reviewer for bringing up this important point. Indeed, the duration of RNA metabolic labeling can affect the accuracy of kinetic rate estimation. The optimal labeling time for any given RNA species is linked to its turnover rate as previously discussed (Uvarovskii *et al*, 2019; Hersch *et al*, 2022). However, the optimal labeling time may vary depending on specific genes. Therefore, finding a 'sweet spot' between shorter and longer 4sU labeling times is crucial to obtain accurate kinetic rate estimation.

The mean half-life of RNA molecules in HEK293 cells is known to be around 5 hours (Schwanhäusser *et al*, 2011). The cell cycle-regulated genes generally have shorter half-lives (median around 1.1 hour in our calculation). When using the simplified kinetic rate model, we found that 60 minutes 4sU labeled samples resulted in more accurate calculations of the RNA kinetic rates using simulations (Fig S6D). Since the gene expression profiles were simulated based on real RNA half-lives in the cell cycle, the 60 minutes is more likely to be the 'sweet spot' compared to the others (15 minutes and 30 minutes) for the cell cycle gene kinetic rates calculation.

7. To demonstrate the applicability to other models, the authors could try to apply Eskrate on the sci-fate dataset (PMID: 32284584). They can restrict the analysis to the unstimulated cells of the dataset and investigate the RNA kinetics over the cell cycle in A549 cells. This will show if the biological conclusions are transposable to another cell line and if the workflow applies to longer labeling times (2 hours). In addition, the sci-fate dataset has a higher percentage of intronic reads. This might be a good opportunity for the authors to run Eskrate with the full model, including processing rates.

We thank the review for this suggestion. The sci-fate dataset is a good application for our method. We were able to calculate RNA transcription and degradation rates from the A549 cells using the method we implemented. The comparison of common cell cycle variable genes between sci-fate data and SLAM-drop-seq data has shown very high similar patterns of the transcription and degradation rates between both datasets. This comparison has been added to our results (Fig. EV2 D-F).
For the splicing rate, unfortunately the precursor fraction per cell for the cell cycle variable genes are still very sparse (see below plot). We thus are not confident to infer the splicing rate using the full model.

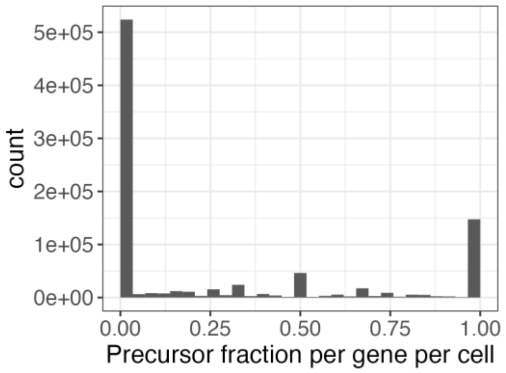

8. The authors have tried different 4sU labeling times (15, 30, and 60min) but have restrained their analysis for kinetic rate calculation to the cells treated for 60min. The reason is clearly explained by the authors. Nevertheless, the authors report on 5 to 10% new RNA after 15 and 30min labeling (Fig 1E). To my knowledge, this is the first time that I see such short times for a SLAM-seq-based approach. Do the authors think that their estimation of newly synthesized RNA is still accurate with 15min labeling (beyond the accuracy of kinetic rate estimation)? This is important information for the community to design future experiments

This is a very helpful comment to consider. For the SLAM-seq-based approach which applies nucleotides conversions, we showed that the newly synthesized molecules roughly linearly increased along 4sU labeling times (Fig. 1E), indicating that we captured newly synthesized RNAs in short labeling times (i.e., 15 minutes and 30 minutes) as efficiently as longer labeling times (60 minutes).

Reviewer #3

This paper introduces a useful methodology SLAM-Drop-seq, a method that allows the analysis of nascent and mature transcripts from single cells. The advantage of SLAM-Drop-seq over other methods such as scEU-seq is that no extra purifications are needed. The paper also introduces an R package Eskrate to optimally analyze SLAM-Drop data. The protocols and methods are clearly described.

As a test they then analyze mRNA expression levels during the mammalian cell cycle,showing that mRNA degradation is an important contribution of the cyclic expression profiles. The conclusions on the cell cycle RNA profiles are similar to those reached by Battich Science 2020 using scEU-seq, however, the Battich analysis went deeper. Overall the biological analysis is fairly succinct as it seems the emphasis is on the methodology, both experimental and computational. Given the extensive data that were generated, and elaborate modeling that was developed, it appears that these were not exploited to their full potential (Figures 3 and 4).

Major points:

1. Some results seem qualitatively different from those of Battich (RPE cells). For example here, there is a majority of genes with transcription starting in early G1 (Fig 3B), which in Battich there are no such genes, there is always a significant gap time after mitosis without transcription.

We thank the reviewer for the comment. The differences observed between our study and the Battich paper can be attributed, in part, to differences in the experimental approaches, cell lines used, and computational analyses. The use of the FUCCI reporter and FACS-sorting based on its fluorescence in the Battich paper introduces variations that cannot be easily accounted for. Additionally, the RPE1 cells used in Battich paper have distinct characteristics from the HEK293 cell line used in our study. Thus, differences in the results are expected.

Furthermore, the absence of transcriptional activity in the beginning of G1 in the Battich paper may be a result of their experimental design. The scEU-seq technique estimates cell cycle time using the FUCCI system, which does not emit fluorescence at the beginning of G1. This increases the noise and unreliability in accurately estimating cell cycle time.
We were not surprised to find genes transcribed in the early G1 phase, despite the belief that RNA synthesis shuts down after cell division. Recent research has described how some transcription factors remain bound to DNA throughout the M-to-G1 transition and drive gene expression soon after cell division. This phenomenon, known as 'transcription factors bookmarking,' could explain our findings, but further research is necessary to support this hypothesis.

2. More generally, since the Battich paper is a reference, it seems essential to compare the findings in more detail (gene by gene), and investigate whether the differences are of technical origin (sequencing or analysis), or have a biological significance.

We thank the reviewer for the important suggestion. We have added the Battich paper results as a reference to our manuscript. The comparison of common variable cell cycle genes between scEU and our data has been added and can be viewed in Fig. EV2 A-C now. We have also added a paragraph in the results describing the findings and the possible reasons for the observed differences.

3. There is a claim in the Discussion that the rates inferred here are better resolved temporally (less than one minute) that other approaches, but this is not shown in the paper and also not exploited to illustrate some interesting biology.

We appreciate the comment from the reviewer and acknowledge the unclear claim in the discussion. Our study achieved high time resolution by utilizing prior knowledge of the median cell cycle time in HEK293 cells (Cheng & Solomon, 2008) and assigning each single cell to a specific time point based on its gene expression profile. For the kinetic rates shown in the manuscript, we sorted approximately 1800 single cells along a whole cell cycle of 19.33 hours (1159.8 minutes), resulting in a time duration of less than one minute between two adjacent cells. Therefore, we were able to theoretically obtain RNA kinetic rates for all single cells along the sorted cell cycle, which justifies our claim of high cell cycle time resolution. In practice, we smoothed gene expression profiles along the cell cycle and implied assumption (*i.e.*, kinetic rate changes in a short cell cycle time interval could be neglected) to solve the kinetic rate mathematical framework. Therefore, differences in kinetic rates of adjacent cells in a short time interval could represent smoothed values that neglect cell-to-cell variability. The claim is more accurate on the theoretical view. We have updated our discussion to clarify this point.

4. "Fig S5C: The number of mean molecules per bin gradually increases along the cell cycle progression and roughly halves after the cell division." It appears the data go from about 0.6 to 0.8, which is significantly less than a factor of 2. This could be due to either inaccurate cell cycle phase assignment or an issue with the experimental approach.

We thank the reviewer for pointing out this mistake. The caption of Figure S5C (now updated to Figure EV1 C) has been corrected, because it was wrongly describing the displayed figure. In a previous experiment, we did reproduce the doubling of total RNA molecules along the cell cycle as described in (Schwabe *et al*, 2020), but couldn't use the generated data for other reasons. In the new batch of the experiment, the total molecules increase towards the M phase (Fig EV1 C), but the increasing factor is less than 2, as correctly described by reviewer #3. This could be due to the fact that the capture rate (*i.e.*, number of UMIs and genes per cell) is not relatively high in terms of scRNA-seq data. Schwabe et al. has shown that the UMI doubling pattern was less clear in HEK293 and 3T3 cells data compared to deeper Hela cell data.

5. From a cell cycle perspective, the insights are fairly limited. The finding that mRNA degradation plasma significant was shown in Battich 2020. It is valuable to confirm this, but it hold be augmented with more explicit comparisons.

We thank the reviewer for the comment and suggestion. While some of our findings may overlap with those of the Battich et al. 2020 paper, there are significant differences between the two studies. One major difference is the identity of genes with variable expression across the cell cycle. Additionally, the proportion of transcripts across different modes of gene expression regulation is distinct in our dataset. Lastly, we observed that gene expression for a fraction of cycling genes peaks at the beginning of the G1 phase, which contrasts with the findings reported in the Battich et al. 2020 paper. A detailed gene-by-gene comparison of mRNA levels, transcription and degradation rates has been added in Figure EV2 A-C.

6. Cell-cycle biology: one histone transcript is being discussed, but this is confirmatory rather than novel. Also, since histone genes are not polyadenylated, this may be a very atypical gene. Can it be measured reliably?

We chose the HIST1H4C histone gene to validate our analysis. HIST1H4C has well-annotated regulation of its kinetic rates during the cell cycle of human cells (Harris *et al*, 1991).

While the presence of the non-polyadenylated transcript of HIST1H4C in our poly(A) single-cell library may be atypical for histone genes, its high abundance in proliferating cells and the presence of a stretch of 20 nucleotides with 18 adenosine in its 3'-end increase the likelihood of capturing the histone transcript by poly(dT)-containing beads. The high CPM counts and low dropout rate of the HIST1H4C gene (Fig. 3C) indicates a high level of reliability in the associated estimates and measurements.

7. How typical are genetically unstable HEK293 cells for human cell cycle biology? It would be useful to have a comparative angle to know how general the findings are.

With respect to other human cells, HEK293 exhibit a similar pattern of the cell cycle, with distinct phases of G1, S, G2 and M. However, due to their different genetics, they may exhibit variation in the duration of these phases, their checkpoint regulation and gene expression. We are aware that HEK293 cells may not perfectly represent all aspects of human cell cycle biology, but they have already been successfully used to study human cell cycle regulation (with findings that have been correlated to homologous genes in different organism(Davidson *et al*, 2009)) and its implication in cancer (Liu *et al*, 2020) . HEK293 cells are also an uncomplicated biological model to develop new experimental approaches such as SLAM-Drop-seq. Moreover, we chose HEK293 cells because they are fast-cycling cells, and in order to study the cell cycle profiles of synthesis and degradation for hundreds of transcripts at the single cell level.

Minor:

Labeling times of 0 15, 30 and 60 minutes are described. Are all used in the subsequent analyses and models?

We thank the reviewer for the comment. All the RNA kinetic rates results shown in the manuscript were from the 60 minutes 4sU-labeled sample. This is mainly due to two reasons: (1) longer labeling time results in lower dropouts for nascent RNAs which give us the chance to explore more genes; (2) the 60 minutes labeling samples are more accurately calculated using our simplified mathematical framework (Fig S6D).

BIBLIOGRAPHY

Alles J, Karaiskos N, Praktiknjo SD, Grosswendt S, Wahle P, Ruffault P-L, Ayoub S, Schreyer L, Boltengagen A, Birchmeier C, *et al* (2017) Cell fixation and preservation for droplet-based single-cell transcriptomics. *BMC Biol* 15: 44

Altieri JAC & Hertel KJ (2021) The influence of 4-thiouridine labeling on pre-mRNA splicing outcomes. *Plos One* 16: e0257503

Burger K, Mühl B, Kellner M, Rohrmoser M, Gruber-Eber A, Windhager L, Friedel CC, Dölken L & Eick D (2013) 4-thiouridine inhibits rRNA synthesis and causes a nucleolar stress response. *Rna Biol* 10: 1623–1630

Cheng A & Solomon MJ (2008) Speedy/Ringo C regulates S and G $_2$ phase progression in human cells. *Cell Cycle* 7: 3037–3047

Davidson G, Shen J, Huang Y-L, Su Y, Karaulanov E, Bartscherer K, Hassler C, Stannek P, Boutros M & Niehrs C (2009) Cell Cycle Control of Wnt Receptor Activation. *Dev Cell* 17: 788–799

Hafner M, Landthaler M, Burger L, Khorshid M, Hausser J, Berninger P, Rothballer A, Jr. M, Jungkamp A-C, Munschauer M, *et al* (2009) Transcriptome-wide Identification of RNA-Binding Protein and MicroRNA Target Sites by PAR-CLIP. *Cell* 141

Harris ME, Böhni R, Schneiderman MH, Ramamurthy L, Schümperli D & Marzluff WF (1991) Regulation of histone mRNA in the unperturbed cell cycle: evidence suggesting control at two posttranscriptional steps. *Mol Cell Biol* 11: 2416–2424

Hersch M, Biasini A, Marques AC & Bergmann S (2022) Estimating RNA dynamics using one time point for one sample in a single-pulse metabolic labeling experiment. *Bmc Bioinformatics* 23: 147

Jürges C, Dölken L & Erhard F (2018) Dissecting newly transcribed and old RNA using GRAND-SLAM. *Bioinformatics* 34: i218–i226

Liu H, Dilger JP & Lin J (2020) The Role of Transient Receptor Potential Melastatin 7 (TRPM7) in Cell Viability: A Potential Target to Suppress Breast Cancer Cell Cycle. *Cancers* 12: 131

Macosko EZ, Basu A, Satija R, Nemesh J, Shekhar K, Goldman M, Tirosh I, Bialas AR, Kamitaki N, Martersteck EM, *et al* (2015) Highly Parallel Genome-wide Expression Profiling of Individual Cells Using Nanoliter Droplets. *Cell* 161: 1202–1214

Schwabe D, Formichetti S, Junker JP, Falcke M & Rajewsky N (2020) The transcriptome dynamics of single cells during the cell cycle. *Mol Syst Biol* 16: e9946

Schwanhäusser B, Busse D, Li N, Dittmar G, Schuchhardt J, Wolf J, Chen W & Selbach M (2011) Global quantification of mammalian gene expression control. *Nature* 473

Uvarovskii A, Vries ISN & Dieterich C (2019) On the optimal design of metabolic RNA labeling experiments. *Plos Comput Biol* 15: e1007252

Wang X, Yu L & Wu AR (2021) The effect of methanol fixation on single-cell RNA sequencing data. *Bmc Genomics* 22: 420

Yamawaki TM, Lu DR, Ellwanger DC, Bhatt D, Manzanillo P, Arias V, Zhou H, Yoon OK, Homann O, Wang S, *et al* (2021) Systematic comparison of high-throughput single-cell RNA-seq methods for immune cell profiling. *Bmc Genomics* 22: 66

5th Jul 2023

Manuscript Number: MSB-2022-11427R
Title: SLAM-Drop-seq reveals mRNA kinetic rates throughout the cell cycle

Dear Dr. Landthaler,

Thank you for sending us your revised manuscript. My colleague Jingyi Hou is currently on parental leave and I have taken over your manuscript. We have now heard back from the two reviewers who were asked to evaluate your revised study. As you will see below, the reviewers are satisfied with the performed revisions and support publication. I am glad to inform you that we can accept your manuscript for publication, pending some editorial issues listed below.

- Our data editors have noted some missing information in the figure legends, please see the attached .doc file. Please make all requested text changes using the attached file and *keeping the "track changes" mode* so that we can easily access the edits made.

- Please include 5 keywords.

- The callout for Fig. 1C should be after the callout for Fig. 1B in the main text.

- Please include callouts to Fig. EV1A-B, EV1D.

- There are callouts for Fig. EV1E and Appendix Fig S6D, but no such panels exist, please check.

- Appendix Table S1 should be renamed to Dataset EV1. The Dataset description should be provided as a separate sheet in Excel file. Please make sure that a callout to Dataset EV1 is included in the text.

Please resubmit your revised manuscript online, with a cover letter listing amendments and responses to each point raised by the referees. Please resubmit the paper **within one month** and ideally as soon as possible. If we do not receive the revised manuscript within this time period, the file might be closed and any subsequent resubmission would be treated as a new manuscript. Please use the Manuscript Number (above) in all correspondence.

Click on the link below to submit your revised paper.

https://msb.msubmit.net/cgi-bin/main.plex

As a matter of course, please make sure that you have correctly followed the instructions for authors as given on the submission website.

Thank you for submitting this paper to Molecular Systems Biology.

Yours sincerely,

Maria Polychronidou, PhD
Senior Editor
Molecular Systems Biology

If you do choose to resubmit, please click on the link below to submit the revision online before 4th Aug 2023.

https://msb.msubmit.net/cgi-bin/main.plex

IMPORTANT:
Please note that corresponding authors are required to supply an ORCID ID for their name upon submission of a revised manuscript (EMBO Press signed a joint statement to encourage ORCID adoption).
(https://www.embopress.org/page/journal/17444292/authorguide#editorialprocess)
Currently, our records indicate that the ORCID for your account is 0000-0002-1075-8734.

Please click the link below to modify this ORCID:
Link Not Available

The system will prompt you to fill in your funding and payment information. This will allow Wiley to send you a quote for the article processing charge (APC) in case of acceptance. This quote takes into account any reduction or fee waivers that you may be eligible for. Authors do not need to pay any fees before their manuscript is accepted and transferred to the publisher.

As a matter of course, please make sure that you have correctly followed the instructions for authors as given on the submission website.

*** PLEASE NOTE *** As part of the EMBO Press transparent editorial process initiative (see our Editorial at https://dx.doi.org/10.1038/msb.2010.72 , Molecular Systems Biology will publish online a Review Process File to accompany accepted manuscripts. When preparing your letter of response, please be aware that in the event of acceptance, your cover letter/point-by-point document will be included as part of this File, which will be available to the scientific community. More information about this initiative is available in our Instructions to Authors. If you have any questions about this initiative, please contact the editorial office (msb@embo.org).

Reviewer #1:

The authors have nicely explained the limits of their method and integrated new datasets. The authors have addressed all my concerns and I have no further comments.

Reviewer #3:

The authors have done an excellent revision job. All my points are addressed satisfactorily. I was confused though about the figure number in the rebuttal letter (EV vs Supp figures). Unless I missed it I could not find EV figures among the files available on the MSB website. So please check that everything is ok.

4th Aug 2023

Manuscript number: MSB-2022-11427RR
Title: SLAM-Drop-seq reveals mRNA kinetic rates throughout the cell cycle

Dear Dr Landthaler,

Thank you again for sending us your revised manuscript. We are now satisfied with the modifications made and I am pleased to inform you that your paper has been accepted for publication.

*** PLEASE NOTE *** As part of the EMBO Publications transparent editorial process initiative (see our Editorial at https://dx.doi.org/10.103/msb.2010.72), Molecular Systems Biology publishes online a Review Process File with each accepted manuscripts. This file will be published in conjunction with your paper and will include the anonymous referee reports, your point- by-point response and all pertinent correspondence relating to the manuscript. If you do NOT want this File to be published, please inform the editorial office at msb@embo.org within 14 days upon receipt of the present letter.

LICENSE AND PAYMENT:
All articles published in Molecular Systems Biology are fully open access: immediately and freely available to read, download and share.

Molecular Systems Biology charges an article processing charge (APC) to cover the publication costs. You, as the corresponding author for this manuscript, should have already received a quote with the article processing fee separately. Please let us know in case this quote has not been received.

Once your article is at Wiley for editorial production, you will receive an email from Wiley's Author Services system, which will ask you to log in and will present you with the publication license form for completion. Within the same system the publication fee can be paid by credit card, an invoice or pro forma can be requested.

Payment of the publication charge and the signed Open Access Agreement form must be received before the article can be published online.

Upon acceptance it is mandatory for you to return the completed payment form. Failure to send back the form may result in a delay in the publication of your paper.

Molecular Systems Biology articles are published under the Creative Commons licence CC BY, which facilitates the sharing of scientific information by reducing legal barriers, while mandating attribution of the source in accordance to standard scholarly practice.

Proofs will be forwarded to you within the next 2-3 weeks.

Thank you very much for submitting your work to Molecular Systems Biology.

Kind regards,

Maria

Maria Polychronidou, PhD
Senior Editor
Molecular Systems Biology

EMBO Press Author Checklist

Corresponding Author Name: Nikolaus Rajewsky, Markus Landthaler
Journal Submitted to: Molecular Systems Biology
Manuscript Number: MSB-2022-11427

USEFUL LINKS FOR COMPLETING THIS FORM
The EMBO Journal - Author Guidelines
EMBO Reports - Author Guidelines
Molecular Systems Biology - Author Guidelines
EMBO Molecular Medicine - Author Guidelines

Reporting Checklist for Life Science Articles (updated January 2022)
This checklist is adapted from Materials Design Analysis Reporting (MDAR) Checklist for Authors. MDAR establishes a minimum set of requirements in transparent reporting in the life sciences (see Statement of Task: 10.31222/osf.io/9sm4x). Please follow the journal's guidelines in preparing your manuscript.
Please note that a copy of this checklist will be published alongside your article.

Abridged guidelines for figures
1. Data
The data shown in figures should satisfy the following conditions:
- the data were obtained and processed according to the field's best practice and are presented to reflect the results of the experiments in an accurate and unbiased manner.
- ideally, figure panels should include only measurements that are directly comparable to each other and obtained with the same assay.
- plots include clearly labeled error bars for independent experiments and sample sizes. Unless justified, error bars should not be shown for technical replicates.
- if n<5, the individual data points from each experiment should be plotted. Any statistical test employed should be justified.
- Source Data should be included to report the data underlying figures according to the guidelines set out in the authorship guidelines on Data Presentation.

2. Captions
Each figure caption should contain the following information, for each panel where they are relevant:
- a specification of the experimental system investigated (eg cell line, species name).
- the assay(s) and method(s) used to carry out the reported observations and measurements.
- an explicit mention of the biological and chemical entity(ies) that are being measured.
- an explicit mention of the biological and chemical entity(ies) that are altered/varied/perturbed in a controlled manner.
- the exact sample size (n) for each experimental group/condition, given as a number, not a range;
- a description of the sample collection allowing the reader to understand whether the samples represent technical or biological replicates (including how many animals, litters, cultures, etc.).
- a statement of how many times the experiment shown was independently replicated in the laboratory.
- definitions of statistical methods and measures:
  - common tests, such as t-test (please specify whether paired vs. unpaired), simple χ2 tests, Wilcoxon and Mann-Whitney tests, can be unambiguously identified by name only, but more complex techniques should be described in the methods section;
  - are tests one-sided or two-sided?
  - are there adjustments for multiple comparisons?
  - exact statistical test results, e.g., P values = x but not P values < x;
  - definition of 'center values' as median or average;
  - definition of error bars as s.d. or s.e.m.

Please complete ALL of the questions below.
Select "Not Applicable" only when the requested information is not relevant for your study.

Materials

Newly Created Materials	Information included in the manuscript?	In which section is the information available? (Reagents and Tools Table, Materials and Methods, Figures, Data Availability Section)
New materials and reagents need to be available; do any restrictions apply?	Not Applicable	

Antibodies	Information included in the manuscript?	In which section is the information available? (Reagents and Tools Table, Materials and Methods, Figures, Data Availability Section)
For **antibodies** provide the following information:<br>- Commercial antibodies: RRID (if possible) or supplier name, catalogue number and or/clone number<br>- Non-commercial: RRID or citation	Not Applicable	

DNA and RNA sequences	Information included in the manuscript?	In which section is the information available? (Reagents and Tools Table, Materials and Methods, Figures, Data Availability Section)
**Short novel DNA or RNA including primers, probes**: provide the sequences.	Yes	Materials and Methods

Cell materials	Information included in the manuscript?	In which section is the information available? (Reagents and Tools Table, Materials and Methods, Figures, Data Availability Section)
**Cell lines:** Provide species information, strain. Provide accession number in repository **OR** supplier name, catalog number, clone number, and/**OR** RRID.	Yes	Invitrogen™<br>Flp-In™ T-REx™ 293 Cell Line; Catalog number: R78007
**Primary cultures:** Provide species, strain, sex of origin, genetic modification status.	Not Applicable	
Report if the cell lines were recently **authenticated** (e.g., by STR profiling) and tested for mycoplasma contamination.	Yes	Materials and Methods

Experimental animals	Information included in the manuscript?	In which section is the information available? (Reagents and Tools Table, Materials and Methods, Figures, Data Availability Section)
**Laboratory animals or Model organisms:** Provide species, strain, sex, age, genetic modification status. Provide accession number in repository **OR** supplier name, catalog number, clone number, **OR** RRID.	Not Applicable	
**Animal observed in or captured from the field:** Provide species, sex, and age where possible.	Not Applicable	
Please detail **housing and husbandry conditions**.	Not Applicable	

Plants and microbes	Information included in the manuscript?	In which section is the information available? (Reagents and Tools Table, Materials and Methods, Figures, Data Availability Section)
**Plants:** provide species and strain, ecotype and cultivar where relevant, unique accession number if available, and source (including location for collected wild specimens).	Not Applicable	
**Microbes:** provide species and strain, unique accession number if available, and source.	Not Applicable	

Human research participants	Information included in the manuscript?	In which section is the information available? (Reagents and Tools Table, Materials and Methods, Figures, Data Availability Section)
If collected and within the bounds of privacy constraints report on age, sex and gender or ethnicity for all study participants.	Not Applicable	

Core facilities	Information included in the manuscript?	In which section is the information available? (Reagents and Tools Table, Materials and Methods, Figures, Data Availability Section)
If your work benefited from core facilities, was their service mentioned in the acknowledgments section?	Yes	Acknowledgments

Design

Study protocol	Information included in the manuscript?	In which section is the information available? (Reagents and Tools Table, Materials and Methods, Figures, Data Availability Section)
If study protocol has been **pre-registered, provide DOI in the manuscript**. For clinical trials, provide the trial registration number **OR** cite DOI.	Not Applicable	
Report the **clinical trial registration number** (at ClinicalTrials.gov or equivalent), where applicable.	Not Applicable	

Laboratory protocol	Information included in the manuscript?	In which section is the information available? (Reagents and Tools Table, Materials and Methods, Figures, Data Availability Section)
Provide DOI OR other citation details if **external detailed step-by-step protocols** are available.	Not Applicable	

Experimental study design and statistics	Information included in the manuscript?	In which section is the information available? (Reagents and Tools Table, Materials and Methods, Figures, Data Availability Section)
Include a statement about **sample size** estimate even if no statistical methods were used.	Yes	Materials and Methods, Figures
Were any steps taken to minimize the effects of subjective bias when allocating animals/samples to treatment (e.g. **randomization procedure**)? If yes, have they been described?	Not Applicable	
Include a statement about **blinding** even if no blinding was done.	Not Applicable	
Describe **inclusion/exclusion criteria** if samples or animals were excluded from the analysis. Were the criteria pre-established?<br><br>If sample or data points were omitted from analysis, report if this was due to attrition or intentional exclusion and provide justification.	Not Applicable	
For every figure, are **statistical tests** justified as appropriate? Do the data meet the assumptions of the tests (e.g., normal distribution)? Describe any methods used to assess it. Is there an estimate of variation within each group of data? Is the variance similar between the groups that are being statistically compared?	Yes	Materials and Methods, Figures

Sample definition and in-laboratory replication	Information included in the manuscript?	In which section is the information available? (Reagents and Tools Table, Materials and Methods, Figures, Data Availability Section)
In the figure legends: state number of times the experiment was **replicated** in laboratory.	Yes	Figures
In the figure legends: define whether data describe **technical or biological replicates**.	Yes	Figures

Ethics

Ethics	Information included in the manuscript?	In which section is the information available? (Reagents and Tools Table, Materials and Methods, Figures, Data Availability Section)
Studies involving **human participants**: State details of **authority granting ethics approval** (IRB or equivalent committee(s), provide reference number for approval.	Not Applicable	
Studies involving **human participants**: Include a statement confirming that **informed consent** was obtained from all subjects and that the experiments conformed to the principles set out in the WMA Declaration of Helsinki and the Department of Health and Human Services Belmont Report.	Not Applicable	
Studies involving **human participants:** For publication of **patient photos**, include a statement confirming that consent to publish was obtained.	Not Applicable	
Studies involving experimental **animals**: State details of **authority granting ethics approva**l (IRB or equivalent committee(s), provide reference number for approval. Include a statement of compliance with ethical regulations.	Not Applicable	
Studies involving **specimen and field samples:** State if relevant **permits** obtained, provide details of authority approving study; if none were required, explain why.	Not Applicable	

Dual Use Research of Concern (DURC)	Information included in the manuscript?	In which section is the information available? (Reagents and Tools Table, Materials and Methods, Figures, Data Availability Section)
Could your study fall under dual use research restrictions? Please check biosecurity documents and list of **select agents and toxins** (CDC): https://www.selectagents.gov/sat/list.htm	Not Applicable	
If you used a select agent, is the security level of the lab appropriate and reported in the manuscript?	Not Applicable	
If a study is subject to dual use research of concern regulations, is the name of the **authority granting approval and reference number** for the regulatory approval provided in the manuscript?	Not Applicable	

Reporting

The MDAR framework recommends adoption of discipline-specific guidelines, established and endorsed through community initiatives. Journals have their own policy about requiring specific guidelines and recommendations to complement MDAR.

Adherence to community standards	Information included in the manuscript?	In which section is the information available? (Reagents and Tools Table, Materials and Methods, Figures, Data Availability Section)
State if relevant guidelines or checklists (e.g., **ICMJE, MIBBI, ARRIVE, PRISMA**) have been followed or provided.	Not Applicable	
For **tumor marker prognostic studies,** we recommend that you follow the **REMARK** reporting guidelines (see link list at top right). See author guidelines, under 'Reporting Guidelines'. Please confirm you have followed these guidelines.	Not Applicable	
For **phase II and III randomized controlled trials**, please refer to the **CONSORT** flow diagram (see link list at top right) and submit the CONSORT checklist (see link list at top right) with your submission. See author guidelines, under 'Reporting Guidelines'. Please confirm you have submitted this list.	Not Applicable	

Data Availability

Data availability	Information included in the manuscript?	In which section is the information available? (Reagents and Tools Table, Materials and Methods, Figures, Data Availability Section)
Have **primary datasets** been deposited according to the journal's guidelines (see 'Data Deposition' section) and the respective accession numbers provided in the Data Availability Section?	Yes	Data Availability
Were **human clinical and genomic datasets** deposited in a public access-controlled repository in accordance to ethical obligations to the patients and to the applicable consent agreement?	Not Applicable	
Are **computational models** that are central and integral to a study available without restrictions in a machine-readable form? Were the relevant accession numbers or links provided?	Yes	Data Availability
If publicly available data were reused, provide the respective **data citations in the reference list**.	Yes	
